# Supplementary material for: Discussing surgical innovation with patients: a qualitative study of surgeons’ and governance representatives’ views
Source: BMJ Open. 2020 Nov 6;10(11):e035251. doi: 10.1136/bmjopen-2019-035251 (PMC7651722; doi:10.1136/bmjopen-2019-035251)
Supplement: Supplementary data [file bmjopen-2019-035251supp002.pdf]

Topic guide for interviews with governance representatives

Introduction:
- Thank for taking time
- Not medical professional, want to understand process of implementing new procedures and devices
- No right or wrong answers, can stop the interview at any time
- Take verbal consent

Can you tell me about yourself?
- What is your current position and role?
- Particular areas of interest/expertise?
- How did you become involved in [committee]? When? How found it? What you expected?
- Can you tell me about the committee? What is the role of the committee?
    - Who involved? How often meet?

As you know, we are looking at innovation in surgery. Can you talk me through how you would define innovation? *(Newness; degree of change, level of risk, impact?)*

Can you talk me through how applications are processed, from submissions to decision-making?
- What is the process for introducing surgical innovation in [site]?
- Do you think there are similarities/difference between innovation and research? In what ways?
- How many applications do you receive each year?
- What information is provided? *(What is compulsory?)*
- How are they considered?
    - Potential benefit? Safety (and how quantified?) Evidence? Cost? Patient acceptability?
- Why would an application be declined? Example? Agreement between committee members?
- What happens to those procedures that are declined? Is there an option to appeal?

What's your understanding of how these procedures are integrated into practice?
- What happens once a technique/device is approved by the committee?
- If approved, is there any monitoring? How? When?
- What if there is a problem with a novel procedure?
- Is there any guidance for communicating about novel procedures?
- What do you think patients are told about the novel component? What should they be told - What aspects important to communicate?
- What is included on patient info sheets provided

Your views on the committee
- How do you feel about being involved in the decision-making process?
- Are there any particularly challenging aspects?
- What are the strengths of the committee?
- Do you think it could be improved in anyway?

Ending the interview
- Is there anything else that I haven't mentioned?
- Anyone else you recommend I could contact?

- Before we finish (if not covered) – role (registrar/consultant), years in your profession, highest qualification
