# Supplementary material for: Discussing surgical innovation with patients: a qualitative study of surgeons’ and governance representatives’ views
Source: BMJ Open. 2020 Nov 6;10(11):e035251. doi: 10.1136/bmjopen-2019-035251 (PMC7651722; doi:10.1136/bmjopen-2019-035251)
Supplement: Supplementary data [file bmjopen-2019-035251supp003.pdf]

Topic guide for interviews with surgeons

Introduction:

- Thank you for taking time
- Not medical professional, want to understand perspectives of innovation
- No right or wrong answers, can stop the interview at any time
- Take verbal consent

Can you tell me about yourself?

- What is your current position and role?
- Can you describe the range /type(s) of surgical procedures you undertake?
- Particular areas of interest/expertise?

As you know, we are looking at innovation in surgery. Can you talk me through how you would define innovation? *(Newness; degree of change, level of risk, impact?)*

Have you conceptualised a new procedure/device/modification of a procedure?

- How did it come about? When?
- Would you regard this as an innovation or a modification?
- Why do you feel it's needed?
- Who is suitable for the procedure? *(How determined?)*
- What treatment options are currently available to them? What happens if patients don't get the procedure? (Are they disadvantaged in any way?)
- What do you hope will be the outcomes from the new procedure? How estimated?

Do you think patients should be informed about new procedures/devices?

- What does 'informed consent' mean to you?
- Is there any guidance for communicating novel procedure?
- What should they be told? Extent that procedure is novel? Uncertainty? Risks? Harms?
- Is informing patients about a new procedure challenging in anyway?
- How do you think patients will respond to the procedure?
- How much of a modification would you need to do to tell a patient something was new?
- When do you stop telling them it is new?

Looking ahead

- Do you think any subgroups of patients would be more suited to the procedure? Are there any that you would feel less comfortable with having the procedure?
- Do you have any concerns about the procedure? Risks to patients? Costs?
- Do you have any plans to disseminate the results? How? All results (good and bad)? Where? When? Who's responsible for this?

Ending the interview

- Is there anything else that I haven't mentioned?
- Anyone else you recommend I could contact?
- Thank for time, summarise key points.
