# Supplementary material for: Discussing surgical innovation with patients: a qualitative study of surgeons’ and governance representatives’ views
Source: BMJ Open. 2020 Nov 6;10(11):e035251. doi: 10.1136/bmjopen-2019-035251 (PMC7651722; doi:10.1136/bmjopen-2019-035251)
Supplement: Author's manuscript [file bmjopen-2019-035251.draft_revisions.pdf]

BMJ Open

BMJ Open is committed to open peer review. As part of this commitment we make the peer review history of every article we publish publicly available.

When an article is published we post the peer reviewers' comments and the authors' responses online. We also post the versions of the paper that were used during peer review. These are the versions that the peer review comments apply to.

The versions of the paper that follow are the versions that were submitted during the peer review process. They are not the versions of record or the final published versions. They should not be cited or distributed as the published version of this manuscript.

BMJ Open is an open access journal and the full, final, typeset and author-corrected version of record of the manuscript is available on our site with no access controls, subscription charges or pay-per-view fees (http://bmjopen.bmj.com).

If you have any questions on BMJ Open's open peer review process please email info.bmjopen@bmj.com

BMJ Open

Informed consent in the context of surgical innovation: A qualitative study of stakeholders' views

Journal:	*BMJ Open*
Manuscript ID	bmjopen-2019-035251
Article Type:	Original research
Date Submitted by the Author:	24-Oct-2019
Complete List of Authors:	Zahra, Jesmond; University of Bristol, National Institute for Health Research Bristol Biomedical Research Centre Surgical Innovation Theme, Bristol Centre for Surgical Research, Bristol Medical School: Population Health Sciences, University of Bristol<br>Paramasivan, Sangeetha ; University of Bristol, National Institute for Health Research Bristol Biomedical Research Centre Surgical Innovation Theme, Bristol Centre for Surgical Research, Bristol Medical School: Population Health Sciences, University of Bristol<br>Blencowe, Natalie; University of Bristol, National Institute for Health Research Bristol Biomedical Research Centre Surgical Innovation Theme, Bristol Centre for Surgical Research, Bristol Medical School: Population Health Sciences, University of Bristol; University Hospitals Bristol NHS Foundation Trust, Division of Surgery<br>Cousins, Sian; University of Bristol, National Institute for Health Research Bristol Biomedical Research Centre Surgical Innovation Theme, Bristol Centre for Surgical Research, Bristol Medical School: Population Health Sciences, University of Bristol<br>Avery, Kerry; University of Bristol, National Institute for Health Research Bristol Biomedical Research Centre Surgical Innovation Theme, Bristol Centre for Surgical Research, Bristol Medical School: Population Health Sciences, University of Bristol<br>Mathews, Johnny; University of Bristol, National Institute for Health Research Bristol Biomedical Research Centre Surgical Innovation Theme, Bristol Centre for Surgical Research, Bristol Medical School: Population Health Sciences, University of Bristol<br>Main, Barry; University of Bristol, National Institute for Health Research Bristol Biomedical Research Centre Surgical Innovation Theme, Bristol Centre for Surgical Research, Bristol Medical School: Population Health Sciences, University of Bristol; University Hospitals Bristol NHS Foundation Trust<br>McNair, Angus; University of Bristol, National Institute for Health Research Bristol Biomedical Research Centre Surgical Innovation Theme, Bristol Centre for Surgical Research, Bristol Medical School: Population Health Sciences, University of Bristol; North Bristol NHS Trust, GI Surgery<br>Hinchliffe, Robert; University of Bristol, National Institute for Health Research Bristol Biomedical Research Centre Surgical Innovation Theme, Bristol Centre for Surgical Research, Bristol Medical School: Population Health Sciences, University of Bristol; North Bristol NHS Trust, Vascular Services<br>Blazeby, Jane; University of Bristol, National Institute for Health

Research Bristol Biomedical Research Centre Surgical Innovation Theme, Bristol Centre for Surgical Research, Bristol Medical School: Population Health Sciences, University of Bristol; University Hospitals Bristol NHS Foundation Trust
Elliott, Daisy; University of Bristol, National Institute for Health Research Bristol Biomedical Research Centre Surgical Innovation Theme, Bristol Centre for Surgical Research, Bristol Medical School: Population Health Sciences, University of Bristol

Keywords: SURGERY, Health policy < HEALTH SERVICES ADMINISTRATION & MANAGEMENT, QUALITATIVE RESEARCH

SCHOLARONE™ Manuscripts

I, the Submitting Author has the right to grant and does grant on behalf of all authors of the Work (as defined in the below author licence), an exclusive licence and/or a non-exclusive licence for contributions from authors who are: i) UK Crown employees; ii) where BMJ has agreed a CC-BY licence shall apply, and/or iii) in accordance with the terms applicable for US Federal Government officers or employees acting as part of their official duties; on a worldwide, perpetual, irrevocable, royalty-free basis to BMJ Publishing Group Ltd ("BMJ") its licensees and where the relevant Journal is co-owned by BMJ to the co-owners of the Journal, to publish the Work in this journal and any other BMJ products and to exploit all rights, as set out in our licence.

The Submitting Author accepts and understands that any supply made under these terms is made by BMJ to the Submitting Author unless you are acting as an employee on behalf of your employer or a postgraduate student of an affiliated institution which is paying any applicable article publishing charge ("APC") for Open Access articles. Where the Submitting Author wishes to make the Work available on an Open Access basis (and intends to pay the relevant APC), the terms of reuse of such Open Access shall be governed by a Creative Commons licence – details of these licences and which Creative Commons licence will apply to this Work are set out in our licence referred to above.

Other than as permitted in any relevant BMJ Author's Self Archiving Policies, I confirm this Work has not been accepted for publication elsewhere, is not being considered for publication elsewhere and does not duplicate material already published. I confirm all authors consent to publication of this Work and authorise the granting of this licence.

Informed consent in the context of surgical innovation: A qualitative study of stakeholders' views

Submission category: Original article

J Zahra[1], S Paramasivan[1], N.S Blencowe[1,3], S Cousins[1], K Avery[1], J Mathews[1,2], B Main [1,3], A McNair [1,2], R Hinchliffe[1,2], J.M Blazeby[1,3], D Elliott[1]

[1]National Institute for Health Research Bristol Biomedical Research Centre Surgical Innovation Theme, Centre for Surgical Research, Population Health Sciences, Bristol Medical School, University of Bristol, 39 Whatley Road, Clifton, Bristol, UK. BS8 2PS;

[2]North Bristol NHS Trust, Bristol, UK. BS10 5NB.

[3]University Hospitals Bristol NHS Foundation Trust, Bristol, UK. BS2 8HW

Running title: Informed consent for innovative surgery

Keywords: consent, surgery, information provision, innovation, qualitative,

Corresponding author details: Daisy Elliott [daisy.elliott@bristol.ac.uk]

Funding statement: This study was supported by the NIHR Biomedical Research Centre at University Hospitals Bristol NHS Foundation Trust and the University of Bristol, the MRC ConDuCT-II (Collaboration and innovation for Difficult and Complex randomised controlled Trials In Invasive procedures) Hub for Trials Methodology Research (MR/K025643/1). The views expressed in this publication are those of the author(s) and not necessarily those of the NHS, the National Institute for Health Research or the Department of Health and Social Care. BM is a NIHR Clinical Lecturer, AM is funded by a Clinician Scientist Fellowship (NIHR-CS-2017-17-010) from the National Institute for Health Research. NB is funded by an MRC Clinician Scientist Award. *Word count*: 4380

For peer review only - http://bmjopen.bmj.com/site/about/guidelines.xhtml

Abstract

Objectives

Little is known about how innovative surgical procedures are introduced and discussed with patients. This qualitative study aimed to explore stakeholders' perspectives on information provision and consent prior to innovative surgical procedures.

Design

Qualitative study involving semi-structured interviewing. Interviews were audio recorded, transcribed and analysed thematically.

Participants

42 interviews were conducted (26 clinicians and 16 governance representatives).

Setting

Surgeons and governance representatives recruited from various surgical specialities and National Health Service Trusts across England, United Kingdom.

Results

Interviewees found it difficult to define surgical innovation, which raises questions as to whether patients were fully informed about the novel components of surgery. If a procedure was deemed innovative, clinicians reported finding it difficult to establish what information is essential and how much detail is sufficient. Furthermore, governance surrounding written and verbal information provision differed between NHS trusts. Generally, clinicians believed patients held a view that 'new' was best and reported that managing these expectations could be difficult, particularly if patient views aligned with their own.

Conclusions

Currently, there is no standardised approach to information provision and consent for innovative surgery. Further research, embedded into clinical and research practice, would provide a more accurate insight into information provision and patient consent for innovative surgical procedures. Such work is necessary in order to develop guidelines and training programmes that would support clinicians to ensure patients are fully informed prior to innovative surgery.

For peer review only - http://bmjopen.bmj.com/site/about/guidelines.xhtml

Summary box – strengths and limitations

- This study addresses an important gap in research exploring informed consent in the context of surgical innovation.
- The qualitative design of the study helped capture important insights into the type of information clinicians and governance representatives feel patient should receive when being offered innovative surgery.
- We cannot conclude from the current study what information is actually communicated during consultations. Further research, embedded into clinical practice, is needed to better understand patient information provision and consent in the context of innovative surgery.

For peer review only

For peer review only - http://bmjopen.bmj.com/site/about/guidelines.xhtml

Introduction

Informed consent is fundamental to patient autonomy and represents a cornerstone of modern healthcare provision. The 2015 Montgomery ruling redefined the standard of informed consent in the United Kingdom (UK) and represented a shift from a paternalistic model of consent to a more patient-centred approach[1 2]. Clinicians are now required to work in partnership with patients and consider their views, goals and circumstances when establishing best treatment[2].

Recently, important questions have been raised surrounding informed consent for innovative surgical procedures[3 4]. An inquest into the death of the first patient in the UK to undergo robotically assisted heart surgery found the patient had not been fully informed about the comparative risk of robotic versus conventional open surgery[4]. In addition, a summary of invited reviews published by the Royal College of Surgeons of England (RCSEng) raised further concerns[3]. The review highlighted issues surrounding surgeons' ability to accurately quantify risk as reported in published literature, whether surgical experience is being sufficiently disclosed and effectively communicated to patients, and the overall quality of patients-clinician discussions[3]. Consistent with these concerns, previous research has shown patients are not always sufficiently informed prior to surgery, and when questioned post-operatively often have a poor knowledge of their operation[5-7]. Recognising these issues, the RCSEng has recently published guidelines on informed consent for new surgical techniques and technologies[8]. These stipulate that patients should be informed of the innovative nature of the procedure; surgeons' experience with the procedure and the learning curve; the risk and benefits – including unknown or unforeseeable risks or outcomes; the evidence or lack thereof; and alternatives to the innovative procedure[8].

While there is a body of research exploring informed consent in early phase oncological studies[9-11], consent in early phase surgical studies or when new or modified surgical treatments are introduced as part of routine clinical practice (e.g. via trust governance processes) has not previously been explored[12]. The limited evidence available suggests clinicians may vary in how they approach informed consent discussions and, compared to patients, place less value on the disclosure of information relating to surgical outcomes and their personal experience performing the procedure[13 14]. Research conducted in Australia indicates healthcare professionals may alter their approach to informed consent when offering patients innovative surgery, however, how this may differ to consent practice for conventional surgery is currently unknown [15]. The scarcity of research in this area means that little is known about information provision and consent for innovative surgeries that are introduced as part of early phase studies or everyday routine practice[16].

For peer review only - http://bmjopen.bmj.com/site/about/guidelines.xhtml

To address this evidence gap, the current study sought to investigate a sub-group of key stakeholders' views of information provision and consent in the context of surgical innovation. To the best of our knowledge this is the first UK based study to explore consent within this context. It is hoped that this study will lead to further research, and ultimately to the development of comprehensive guidelines for information provision and consent for innovative surgery.

Methods

Design

One-to-one in-depth semi-structured interviews were undertaken. Ethical approval was granted by the University of Bristol Faculty of Health Sciences Research Ethics Committee. This study is reported in line with the SRQR guidelines for qualitative research[17].

Sampling and recruitment

Participants were recruited between November 2017 and October 2018 using a combination of purposive and snowball sampling techniques. Governance representatives, defined as those involved in regulating the introduction of new/modified procedures and/or devices at trust or national levels, were identified through websites (e.g. National Health Service (NHS) Trusts websites) and NHS policy documents (e.g. new invasive procedure/devices policies). An initial sample of surgeons were identified by academic clinicians working within the Centre for Surgical Research, University of Bristol, UK. Subsequent surgeons were identified via snowball sampling, in which interviewees recommended others who may be willing to take part. Sampling was regularly reviewed to ensure clinicians from different specialities and geographical locations, with varying experiences of innovation (e.g. minor modifications vs first in human procedures), were recruited. Potential participants were approached via email and up to three invitation emails were sent before contact attempts ceased. In total, 83 potential participants were invited to take part (38 surgeons and 45 governance representatives). Study recruitment ceased when the qualitative team agreed that saturation had been achieved (i.e. no new themes were forthcoming)[18].

Data collection

All participants received a study information leaflet and provided written consent before taking part. Interviews were flexible to allow participants to shape the direction of conversations, however, interview schedules were developed for individual participant groups to ensure core topics of interest were discussed (e.g. 'How would you define innovation?', 'What does informed consent mean to you?'). These were developed iteratively during the study period, in consideration of emerging insights from interviews. An example of an interview schedule is provided in appendix 1.

For peer review only - http://bmjopen.bmj.com/site/about/guidelines.xhtml

With permission, interviews were audio recorded using an encrypted digital dictaphone. All interviews were conducted by one of two trained and experienced qualitative researchers (JZ or DE); JZ is a Senior Research Associate with a background in public health research and DE is a Research Fellow with experience working on surgical trials.

Data analysis

Interview recordings were transcribed and anonymised before being imported into a data management program (NVivo version 11). Following the guidelines stipulated by Braun and Clarke, data was analysed thematically[18]. First, analysts (JZ, DE & SP) read and re-read transcripts before independently coding a sample of five transcripts. When comparing codes, differences were discussed thoroughly until a consensus was met and an initial coding frame was developed. The coding frame was then independently applied to three transcripts by JZ, DE and SP. High consistency between analysts indicated the coding frame appropriately captured the data. JZ then applied the coding frame to the remaining transcripts, adding additional codes if important data were missed by the original coding frame. To form preliminary themes and subthemes, codes were collated into hierarchical groups. These were continually discussed during team meeting to ensure they fully encapsulated the meaning of the data. Finally, a descriptive account of the themes and subthemes, which included representative quotes, was developed.

Results

Participants

In total, 42 participants were recruited into the study; 26 surgeons and 16 governance representatives from across 23 NHS Trusts. Trusts varied in geographical location, type/size (i.e. teaching, specialist, large, multi-centre) and foundation status. Interviews lasted an average of 43 minutes (range 22 – 112 minutes, standard deviation 17 minutes). Governance representatives' roles within trusts varied (e.g. medical director, director for quality improvement, head of governance), however, all played a role in regulating the introduction of new surgical procedures and/or devices. At the time of interview, all but one (retired) surgeon worked as a consultant within the NHS. Interviews were audio recorded expect one where the interviewee requested not to be recorded and therefore written notes were taken. Interviews were conducted over the phone (55%) or face-to-face at participants' place of work or at a university.

Findings related to informed consent are presented under four main themes; *what is innovation?, what should patients be told?, the challenges of obtaining informed consent in the context of*

For peer review only - http://bmjopen.bmj.com/site/about/guidelines.xhtml

innovation, and *managing patient expectations*. All quotations are followed by participants' unique identification number, gender, surgical speciality and trust identification number.

What is innovation?

Clinicians and governance representatives found it difficult to define surgical innovation. While definitions varied, in general stakeholders considered the term 'innovation' to encompass a spectrum of changes to surgical healthcare. Examples ranged from changing patient pathways through to first in human procedures. Consequently, many felt innovation should be viewed as a spectrum, rather than a binary concept:

> *"Well there's a spectrum isn't there, I mean it's difficult to define. So something that is innovative to me is something that is new, it's a new way of looking at it, it's a slightly different paradigm, a different way of thinking. And an adaptation is just a sort of a natural evolution of something that's already pre-existing, I suppose that's how I would define them, but clearly it's a spectrum isn't it?" (Surgeon 18, General surgeon, Male, Trust 3)*

Data suggests some NHS trusts have policies designed to govern the introduction of new and/or modified surgical procedure. Several interviewees drew upon definitions included in trusts policies to define innovation. Notably, in some trusts if a procedure falls within the remit of a policy (e.g. regarded as 'new') then 'special measures' for patient information provision and consent may be implemented:

> *"So, if it's [procedure] new to the trust, that would be an innovation for the hospital…there's a process for approval of patient information at (name of hospital) so it has – which there's a reader's panel and stuff. So, we have two, a two-pronged attack really, the readers panel which is the normal way that it's approved, and then on top of that the [new procedures] committee will look at it." (Governance representative 44, Chair of clinical effectiveness committee, Female, Trust 19)*

While clinicians may not be solely responsible for deciding whether a procedure should be regarded as innovative, their perspectives may influence the type of information they choose to disclose to patients. The quotes below demonstrate the important role individual perspectives can have on how clinicians approach information provision for the same surgical procedure:

> *"Were we aware of what we sold it [micro-lobectomy] to the patient as, we were doing something similar, moving the position of the posts…we explained to the patients and everything else." (Surgeon 19, Thoracic, Male, Trust 1)*

For peer review only - http://bmjopen.bmj.com/site/about/guidelines.xhtml

"For things like micro lobectomy I don't even explain how that's different to endoscopic lobectomy because I think it's far too technically nuanced for a patient to understand… We view micro lobectomy as just a variation of endoscopic lobectomy." (Surgeon 22, Thoracic, Male, Trust 7)

Without a clear understanding of what constitutes innovation, clinicians may rely on their personal judgement when deciding whether to inform patients of changes to procedures/devices:

"I think absolutely they should be informed, I think that's a tricky one actually… when would I inform a patient about a new instrument I was using? So we don't- if- if you're not trialling the instrument to see if it's safe or more effective or less effective, then I don't think they need to be informed." (Surgeon 4, Male, General and upper gastrointestinal, Trust 12)

What should patients be told?

There was a consensus both across and within participant groups that if a procedure is deemed innovative, patients should be provided with additional information extending beyond that given during routine surgical consultations. The potential risks and benefits, alternative treatment options, and the novel status of the procedure were universally regarded as essential information that should be discussed in this context:

"There is an alternative, and this is the new incentive and the potential benefits of it are, blah blah blah. These are the potential downsides. I'm looking for people to do the new procedure on, would you be willing to consider it?" (Surgeon 6, General and upper gastrointestinal, Male, Trust 1)

Clinicians may decide to disclose further information in addition to these core components, however, the volume and content of this information may vary. Information less frequently discussed by clinicians included the evidence base (or lack thereof), operative experience, training, safety precautions implemented in theatre and national guidance.

"We tell them that it's a new procedure and previously we've told them how many we've done prior to this" (Surgeon 5, Thoracic, Male, Trust 5)

"You have to give them options, you have to let them know what the evidence base is." (Surgeon 46, Trauma & Orthopaedic, Male, Trust 22)

Many interviewees felt strongly that patients should be provided with verbal (i.e. discussions during consultation) and written information (i.e. via a patient information leaflet (PIL)) when being offered innovative surgery. Consistent with this, one governance representative discussed a need to prompt clinicians to develop PILs and consider consent processes:

For peer review only - http://bmjopen.bmj.com/site/about/guidelines.xhtml

"We want to see the patient information leaflets or we want to know the consent process." (Governance Representative 41, Associate medical director, Male, Trust 20)

In some trusts, innovative procedures can only be introduced if an appropriate PIL has been developed. This may also need to be reviewed by a committee to ensure appropriate information is included:

"We will insist that there is a patient guide…an information sheet for the patient…It [PIL] will talk about the relative inexperience of the team, saying that this is new to us, but that we're doing it under conditions where we've got an expert coming to help us, we've been to appropriate training, it's been approved by the organisation as something that we think will be safe, but that it is a new way of doing things." (Governance representative 24, Director for Quality Improvement, Male, Trust 9)

Conversely, other trusts have no mandatory requirements for written information. Furthermore, information provision and consent processes may not be a consideration made by governance representatives involved in approving innovative surgical procedures:

"We wouldn't decide about the consent process within that committee, but that's, that you know, that patient would always have to have consent prior to any of the treatments." (Governance representative 39, Medical device officer, Male, Trust 4)

"Obviously in situations in which the technique might be new then that's (PIL) not something I've specifically addressed but probably should do." (Governance Representative 48, Clinical Effectiveness Lead, Male, Trust 24)

Even if PILs are a mandatory requirement, they may not be reviewed to ensure they included appropriate information. If PILs are not reviewed, key information may be omitted:

"I definitely think they should know it's a new procedure. The way we probably tell (patients) is only in the consent process verbally, I don't recall seeing that in any of our written leaflets." (Governance representative 47, Head of governance, Female, Trust 18)

If patients are not provided with written information the reliance on clinicians to deliver appropriate information is likely to be greater, which was a concern for some governance representatives. To address this issue, one interviewee discussed auditing patient consent practice to check key information was disclosed. According to this interviewee, audit results demonstrate that clinicians may not always inform patients that a procedure is new:

For peer review only - http://bmjopen.bmj.com/site/about/guidelines.xhtml

"So we then audit the first case to make sure that the clinician has been up front with the individual about the fact that this is the first time that this particular procedure has been undertaken…if they haven't, there's been a couple of occasions when they haven't, we tell them off and say do better next time and we audit the second case or whatever, so we're confident they've got it right." (Governance representative 29, Director of research, Male, Trust 5)

The challenges of obtaining informed consent in the context of innovation

As previously discussed, clinicians felt a need to deliver additional information during consultations for innovative surgery. However, data suggest that some clinicians may find this challenging:

"it's just trying to get across not only this fact that this is what it is, but the fact that it's newer than something else which as a standard we use. That's quite difficult trying to get that across, and some patients will understand better than others." (Surgeon 42, Neurosurgery, Male, Trust 13)

At a practical level, clinicians reported finding it difficult to establish what information is essential and how much detail is enough. These issues may be further compounded when faced with time constraints, which limit clinicians' ability to have long and/or multiple discussions with patients:

"We don't have much time …I'm finding that I'm over talking far too much information, but I need to make them aware that they're essentially guinea pigs." (Surgeon 23, Colorectal, Female, Trust 2)

Delivering information to a variety of patients who may differ in their ability to understand complex - and potentially very technical - information was a further challenge. To help overcome this, some clinicians felt it was important to simplify technical information and, when possible, provide alterative mediums of information to aid decision making:

"It's [surgical procedure] such a complex thing to be explaining to people so you have to try and bring it down to simplistic terms but at the same time give it in a way that actually they can process the salient points of the information." (Surgeon 46, Trauma & Orthopaedic, Male, Trust 22)

"I think one of the most important things about when you're making this change is that you give people different forms of access, and of course now it's easy. There are YouTube videos of stuff." (Surgeon 43, Orthopaedic, Male, Trust 2)

Clinicians acknowledged that innovative surgery can be associated with uncertainties, including a lack of knowledge surrounding risks, benefits and long-term outcomes. Clinicians acknowledged that discussing uncertainties may conflict with the professional norms within surgery:

For peer review only - http://bmjopen.bmj.com/site/about/guidelines.xhtml

> *"This is what surgeons find hard. You have to say, "I don't know," and we hate that. But actually, we should say it more often. We should say it more often." (Surgeon 4, General and upper gastrointestinal, Male, Trust 12)*

As a result, when faced with uncertainty surrounding an innovative surgery, clinicians may choose to rely on and disclose their personal opinions during patient consultations:

> *"They were told that it was a new technique, that we weren't sure what the long-term outcomes were going to be but, we thought that it was better than traditional surgery." (Surgeon 16, Urology, Male, Trust 2)*

On the other hand, some expressed a desire to remain impartial and stressed the importance of providing balanced information. Several interviewees described using strategies to help ensure they remained as objective as possible during consultations. Examples discussed included adhering to scripted information and only discussing innovative surgery if a patient specifically requested an alternative to conventional approaches:

> *"You can word a new procedure, this is amazing, fantastic, amazing, it's phenomenal and there's no risks to it, and all the patients will say sure, yeah sign me up. Whereas, this is what we're trying to look for, this is the benefits, these are the potential risks, so on and so forth. Then it becomes much more objective." (Surgeon 25, Thoracic, Male, Trust 14)*

Of note however, the language used to describe conventional surgical approaches during interviews highlights the potential for clinicians – albeit unintentionally – to influence treatment choice. For instance, terms such as 'old fashioned' and 'traditional' were used by some when describing how they approached patient information provision:

> *"I'll tell them, "We're going to try and do it like this. Is that okay? Do you have any objection? The alternative is we're going to do it the old-fashioned way, which is make a big cut in your- on your stomach wall" (Surgeon 4, General & Upper Gastrointestinal Surgeon, Male Trust 12)*

Managing patient expectations

Clinicians felt that patients often had preferences for new treatments and expected these to deliver better outcomes. For instance, clinicians with experience of introducing robotically assisted surgery commonly reported that patients conveyed a strong preference for this approach. Consistent with this, one interviewee felt that patient demand for robotically assisted surgery was a driving factor for hospitals to invest in robotics.

For peer review only - http://bmjopen.bmj.com/site/about/guidelines.xhtml

> *"Patients took to it [robotically assisted surgery] very well. I thought they might be a bit nervous about it, you know the robot taking over or going crazy…patients like the idea of technology. They equate the robot to being an advanced, superior treatment." (Surgeon 31, Colorectal, Male, Trust 8)*

> *"Patients wanted to have robotic surgery, and if you didn't have a robot, the patients would just go to a neighbouring hospital that did. So, it's really patient driven, so if you wanted to be a centre you have to have a robot." (Surgeon 16, Urology, Male, Trust 2)*

In general, interviewees felt patients held a misconception that 'new' surgical procedures and technologies are better than those normally provided. Some clinicians reported that patients had actively sought them out in order to receive innovative surgery:

> *"They tend to have sought you out, because they want something different than the standard. So they're kind of are willing to accept uncertainty and risk because they've already sought out that uncertainty and risk, if you know what I mean." (Surgeon 9, Upper gastrointestinal, Male, Trust 3)*

Several clinicians were concerned that patients did not understand that innovative procedures may carry uncertainties in terms of risk and benefit. Managing patients' preferences, particularly if they align with the personal preferences, appeared challenging for some:

> *"When does a patient turn around and say, 'Well I'll have the old one'? And the answer is never, and that's difficult, because they are immediately signing themselves up for an experiment. And how you describe that to patients is really difficult, because you've got all of your intrinsic biases yourself. Okay, go on then." (Surgeon 43, Orthopaedic, Male, Trust 2)*

> *"I don't think I've had anyone say, 'no, thank you'. I think most of them have gone, 'I don't mind being a guinea pig'. They always use that phrase (laughter). Makes me feel a little bit uncomfortable." (Surgeon 36, Breast, Female, Trust 4)*

Discussion

This study provides novel insights into clinicians' and governance representatives' views on information provision and consent for innovative surgical procedure and represents a significant contribution to the limited empirical literature. Results highlight inconsistencies in how NHS trusts govern informed consent practice for innovative surgery. Furthermore, clinicians' interviews suggest a lack of consistency in the provision of verbal and written information prior to innovative surgical treatment. Data indicate a heavy reliance upon clinicians to decide what and how information is verbally communicated to patients within some trusts. This conflicts with the recommendations

For peer review only - http://bmjopen.bmj.com/site/about/guidelines.xhtml

reported with the RCSEng guidelines, which suggest oversight committees should discuss information provision and consent with clinicians prior to approvals being granted [8]. Collectively, our results stress the need for better oversight and regulation of information provision and consent practice in the context of innovative surgery.

Consistent with previous research, our findings suggest clinicians struggle to clearly define surgical innovation[15], which may impact upon their approach to patient information provision and consent. Prior research has shown patients do not always feel fully informed about the different treatment options available to them[19]. It is likely that the added complexity associated with innovation treatments (e.g. unknown risks and benefits, lack of long-term data, limited experience) makes discussing treatments options particularly challenging for clinicians. Study participants recognised the importance of communicating the risks and benefits, alternatives, and novel status of a procedure to patients. However, it was less common for interviewees to identify prior training, experience with procedure, and the evidence base as important components of consent. This variation suggests there is a lack of clarity surrounding the content of informed consent discussions, meaning that some patients are likely to be better informed than others when making treatment decisions. This is a concern given that information provision has been linked to both patient satisfaction and rates of litigation following surgery[20-22].

Aligning with previous research, our data suggests clinicians may not disclose their personal experience of performing a procedure to patients[14]. Few clinicians identified, or reported discussing, surgical experience and/or training as a component of information provision. Surgical experience has previously been shown to be of importance to patients[14]. While we cannot conclude from the current data, the disclosure of this information could lead to conversations regarding the surgical learning curve and the clinicians' position on it. Moreover, these discussions could help emphasise to patients that a procedure is novel and there are potential uncertainties in terms of risks and benefits.

To the best of our knowledge, this study is the first to explore stakeholders' views of informed consent in the context of innovative surgery. An important strength of the study is the sample of both clinicians and governance representatives, recruited from various surgical specialities, trust types and geographical locations. This allowed us to explore informed consent from two different perspectives, whilst additionally providing an important insight into the role trust governance plays in patient information provision and consent. Nonetheless, there are potential limitations to the study that should be acknowledged. We cannot conclude what information is actually communicated to patients during consultations from interview data alone. Future research should

For peer review only - http://bmjopen.bmj.com/site/about/guidelines.xhtml

capture what actually happens in these interactions by audio recording clinician-patient consultations. Such methods have been used successfully in clinical trials to improve the quality of informed consent and recruitment rates[23-25]. Furthermore, studies exploring patients' views, particularly those who are offered or have received innovative surgery, are critical if we are to understand the type of information patients draw upon when making decisions about their care. Such work is currently being conducted by our research group as part of a larger research study exploring innovation in surgery. It is hoped that this research will aid the development of 'core information sets' (minimum sets of information that should be discussed with patients), which will ultimately support clinicians to discuss innovation and help catalyse the discussion of issues that are important to individual patients[26].

In conclusion, the study suggests that information provision for innovative surgery presents a challenge for clinicians. Our findings also highlight variation in how NHS trusts govern information provision and consent practice for innovative surgery, and further inconsistencies between the views of clinicians and governance representatives. When innovations are introduced under the auspices of research, there are governance structures (e.g. research ethics committees) in place to help ensure patient information is appropriate. However, innovation and modifications to surgery often takes place without formal research approval, which raises the question of how well informed patients are prior to surgery. While the recent RCSEng guidelines for introducing new techniques and technologies represents an important step towards improving informed consent, our study suggests a need to develop support and training programmes to ensure that clinicians consistently deliver appropriate and comprehensive information to patients in the context of innovative surgery.

Authors' contributions

JMB conceptualised the idea to study informed consent and information provision in the context of surgical innovation. DE developed the idea for the current study with support from JB, JZ, SP, SC, NB, BM, AM and KA. NB, JM, AM and BM identified potential participants. JZ and DE conducted the interviews and analysed the data, with regular discussion of data interpretation with SP and JB. The NIHR Bristol Biomedical Research Centre Surgical Innovation theme is directed by JMB (theme lead) and RH (deputy theme lead). The manuscript was written by JZ, with critical revision by DE and contributions and final approval by all remaining authors.

For peer review only - http://bmjopen.bmj.com/site/about/guidelines.xhtml

For peer review only

Disclaimer

The views expressed in this publication are those of the authors and not necessarily those of the NHS, the National Institute for Health Research, the MRC or the Department of Health and Social Care.

Competing interests

None declared

References

1. Faden RR, Beauchamp TL. A history and theory of informed consent: Oxford University Press 1986.
2. Chan SW, Tulloch E, Cooper ES, et al. Montgomery and informed consent: where are we now? 2017;357:j2224.
3. Royal College of Surgeons. Learning from invited reviews. 2019
4. Dyer C. Robot assisted surgery is blamed for heart patient's death. 2018;363:k4791. doi: 10.1136/bmj.k4791 %J BMJ
5. Leclercq WKG, Keulers BJ, Scheltinga MRM, et al. A review of surgical informed consent: past, present, and future. A quest to help patients make better decisions. *World journal of surgery* 2010;34(7):1406-15. doi: 10.1007/s00268-010-0542-0 [published Online First: 2010/04/07]
6. Weckbach S, Kocak T, Reichel H, et al. A survey on patients' knowledge and expectations during informed consent for spinal surgery: can we improve the shared decision-making process? *Patient Safety in Surgery* 2016;10(1):15. doi: 10.1186/s13037-016-0103-z
7. Rothberg MB, Sivalingam SK, Ashraf J, et al. Patients' and Cardiologists' Perceptions of the Benefits of Percutaneous Coronary Intervention for Stable Coronary Disease. *Annals of Internal Medicine* 2010;153(5):307-13. doi: 10.7326/0003-4819-153-5-201009070-00005 %J Annals of Internal Medicine
8. Royal College of Surgeons. Surgical innovation, new techniques and technologies 2019.
9. Brown R, Bylund CL, Siminoff LA, et al. Seeking informed consent to phase I cancer clinical trials: identifying oncologists' communication strategies. 2011;20(4):361-68.
10. Malik L, Mejia A. Informed consent for phase I oncology trials: form, substance and signature. *J Clin Med Res* 2014;6(3):205-08. doi: 10.14740/jocmr1803w [published Online First: 2014/03/31]
11. Marshall PA, Magtanong RV, Leek AC, et al. Negotiating decisions during informed consent for pediatric Phase I oncology trials. *J Empir Res Hum Res Ethics* 2012;7(2):51-59. doi: 10.1525/jer.2012.7.2.51
12. Bracken-Roche D, Bell E, Karpowicz L, et al. Disclosure, consent, and the exercise of patient autonomy in surgical innovation: a systematic content analysis of the conceptual literature. *Account Res* 2014;21(6):331-52. doi: 10.1080/08989621.2013.866045
13. Reitsma AM, Moreno JD. Ethical regulations for innovative surgery: the last frontier? *Journal of the American College of Surgeons* 2002;194(6):792-801. doi: https://doi.org/10.1016/S1072-7515(02)01153-5
14. Lee Char SJ, Hills NK, Lo B, et al. Informed consent for innovative surgery: a survey of patients and surgeons. *Surgery* 2013;153(4):473-80. doi: 10.1016/j.surg.2012.08.068 [published Online First: 2012/12/04]
15. Rogers WA, Lotz M, Hutchison K, et al. Identifying surgical innovation: a qualitative study of surgeons' views. *Ann Surg* 2014;259(2):273-8. doi: 10.1097/SLA.0b013e31829ccc5f [published Online First: 2013/06/22]
16. Mastroianni AC. Liability, regulation and policy in surgical innovation: the cutting edge of research and therapy. *Health Matrix Clevel* 2006;16(2):351-442. [published Online First: 2006/09/05]

For peer review only - http://bmjopen.bmj.com/site/about/guidelines.xhtml

17. O'Brien BC, Harris IB, Beckman TJ, et al. Standards for reporting qualitative research: a synthesis of recommendations. *Academic medicine : journal of the Association of American Medical Colleges* 2014;89(9):1245-51. doi: 10.1097/acm.0000000000000388 [published Online First: 2014/07/01]
18. Braun V, Clarke V. Successful qualitative research: A practical guide for beginners: sage 2013.
19. Mitchell S, Gass J, Hanna M. How Well Informed Do Patients Feel about Their Breast Cancer Surgery Options? Findings from a Nationwide Survey of Women after Lumpectomy and/or Mastectomy. *Journal of the American College of Surgeons* 2018;226(2):134-46.e3. doi: 10.1016/j.jamcollsurg.2017.10.022 [published Online First: 2017/12/17]
20. Ford KE, Cooper LRL. Learning from lawsuits: Ten-years of NHS litigation authority claims against 11 surgical specialities in England. *The Surgeon* 2018;16(1):27-35. doi: https://doi.org/10.1016/j.surge.2016.03.009
21. Veerman MM, van der Woude LA, Tellier MA, et al. A decade of litigation regarding surgical informed consent in the Netherlands. *Patient Education and Counseling* 2019;102(2):340-45. doi: https://doi.org/10.1016/j.pec.2018.08.031
22. Hallock JL, Rios R, Handa VL. Patient satisfaction and informed consent for surgery. *American journal of obstetrics and gynecology* 2017;217(2):181.e1-81.e7. doi: 10.1016/j.ajog.2017.03.020 [published Online First: 2017/04/02]
23. Elliott D, Husbands S, Hamdy FC, et al. Understanding and Improving Recruitment to Randomised Controlled Trials: Qualitative Research Approaches. *European urology* 2017;72(5):789-98. doi: 10.1016/j.eururo.2017.04.036 [published Online First: 2017/06/06]
24. Rooshenas L, Paramasivan S, Jepson M, et al. Intensive Triangulation of Qualitative Research and Quantitative Data to Improve Recruitment to Randomized Trials: The Quintet Approach. 2019;29(5):672-79.
25. Donovan JL, Rooshenas L, Jepson M, et al. Optimising recruitment and informed consent in randomised controlled trials: the development and implementation of the Quintet Recruitment Intervention (QRI). *Trials* 2016;17(1):283. doi: 10.1186/s13063-016-1391-4
26. Main BG, McNair AGK, Huxtable R, et al. Core information sets for informed consent to surgical interventions: baseline information of importance to patients and clinicians. *BMC medical ethics* 2017;18(1):29. doi: 10.1186/s12910-017-0188-7

For peer review only

For peer review only - http://bmjopen.bmj.com/site/about/guidelines.xhtml

Topic guide for interviews stakeholders

Introduction:

- Thank you for taking time
- Not medical professional, want to understand perspectives of innovation
- No right or wrong answers, can stop the interview at any time
- Take verbal consent

Can you tell me about yourself?

- What is your current position and role?
- Can you describe the range /type(s) of surgical procedures you undertake?
- Particular areas of interest/expertise?

As you know, we are looking at innovation in surgery. Can you talk me through how you would define innovation? *(Newness; degree of change, level of risk, impact?)*

Have you conceptualised a new procedure/device/modification of a procedure?

- How did it come about? When?
- Would you regard this as an innovation or a modification?
- Why do you feel it's needed?
- Who is suitable for the procedure? *(How determined?)*
- What treatment options are currently available to them? What happens if patients don't get the procedure? (Are they disadvantaged in any way?)
- What do you hope will be the outcomes from the new procedure? How estimated?

Do you think patients should be informed about new procedures/devices?

- What does 'informed consent' mean to you?
- Is there any guidance for communicating novel procedure?
- What should they be told? Extent that procedure is novel? Uncertainty? Risks? Harms?
- Is informing patients about a new procedure challenging in anyway?
- How do you think patients will respond to the procedure?
- How much of a modification would you need to do to tell a patient something was new?
- When do you stop telling them it is new?

Looking ahead

- Do you think any subgroups of patients would be more suited to the procedure? Are there any that you would feel less comfortable with having the procedure?
- Do you have any concerns about the procedure? Risks to patients? Costs?
- Do you have any plans to disseminate the results? How? All results (good and bad)? Where? When? Who's responsible for this?

Ending the interview

- Is there anything else that I haven't mentioned?
- Anyone else you recommend I could contact?
- Thank for time, summarise key points.

For peer review only

Reporting checklist for qualitative study.

Based on the SRQR guidelines.

Instructions to authors

Complete this checklist by entering the page numbers from your manuscript where readers will find each of the items listed below.

Your article may not currently address all the items on the checklist. Please modify your text to include the missing information. If you are certain that an item does not apply, please write "n/a" and provide a short explanation.

Upload your completed checklist as an extra file when you submit to a journal.

In your methods section, say that you used the SRQRreporting guidelines, and cite them as:

O'Brien BC, Harris IB, Beckman TJ, Reed DA, Cook DA. Standards for reporting qualitative research: a synthesis of recommendations. Acad Med. 2014;89(9):1245-1251.

		Reporting Item	Page Number
**Title**			
	#1	Concise description of the nature and topic of the study identifying the study as qualitative or indicating the approach (e.g. ethnography, grounded theory) or data collection methods (e.g. interview, focus group) is recommended	5
**Abstract**			
	#2	Summary of the key elements of the study using the abstract format of the intended publication; typically includes background, purpose, methods, results and conclusions	2
**Introduction**			
Problem formulation	#3	Description and signifcance of the problem / phenomenon studied: review of relevant theory and empirical work; problem statement	4-5
Purpose or research question	#4	Purpose of the study and specific objectives or questions	5

For peer review only

Methods

Qualitative approach and research paradigm	#5	Qualitative approach (e.g. ethnography, grounded theory, case study, phenomenolgy, narrative research) and guiding theory if appropriate; identifying the research paradigm (e.g. postpositivist, constructivist / interpretivist) is also recommended; rationale. The rationale should briefly discuss the justification for choosing that theory, approach, method or technique rather than other options available; the assumptions and limitations implicit in those choices and how those choices influence study conclusions and transferability. As appropriate the rationale for several items might be discussed together.	5
Researcher characteristics and reflexivity	#6	Researchers' characteristics that may influence the research, including personal attributes, qualifications / experience, relationship with participants, assumptions and / or presuppositions; potential or actual interaction between researchers' characteristics and the research questions, approach, methods, results and / or transferability	5-6
Context	#7	Setting / site and salient contextual factors; rationale	5
Sampling strategy	#8	How and why research participants, documents, or events were selected; criteria for deciding when no further sampling was necessary (e.g. sampling saturation); rationale	5
Ethical issues pertaining to human subjects	#9	Documentation of approval by an appropriate ethics review board and participant consent, or explanation for lack thereof; other confidentiality and data security issues	5
Data collection methods	#10	Types of data collected; details of data collection procedures including (as appropriate) start and stop dates of data collection and analysis, iterative process, triangulation of sources / methods, and modification of procedures in response to evolving study findings; rationale	5-6
Data collection instruments and technologies	#11	Description of instruments (e.g. interview guides, questionnaires) and devices (e.g. audio recorders) used for data collection; if / how the instruments(s) changed over the course of the study	5
Units of study	#12	Number and relevant characteristics of participants, documents, or events included in the study; level of participation (could be	5

For peer review only

		reported in results)	
Data processing	#13	Methods for processing data prior to and during analysis, including transcription, data entry, data management and security, verification of data integrity, data coding, and anonymisation / deidentification of excerpts	5
Data analysis	#14	Process by which inferences, themes, etc. were identified and developed, including the researchers involved in data analysis; usually references a specific paradigm or approach; rationale	5-6
Techniques to enhance trustworthiness	#15	Techniques to enhance trustworthiness and credibility of data analysis (e.g. member checking, audit trail, triangulation); rationale	6
**Results/findings**			
Syntheses and interpretation	#16	Main findings (e.g. interpretations, inferences, and themes); might include development of a theory or model, or integration with prior research or theory	6-12
Links to empirical data	#17	Evidence (e.g. quotes, field notes, text excerpts, photographs) to substantiate analytic findings	6-12
**Discussion**			
Intergration with prior work, implications, transferability and contribution(s) to the field	#18	Short summary of main findings; explanation of how findings and conclusions connect to, support, elaborate on, or challenge conclusions of earlier scholarship; discussion of scope of application / generalizability; identification of unique contributions(s) to scholarship in a discipline or field	12-13
Limitations	#19	Trustworthiness and limitations of findings	13
**Other**			
Conflicts of interest	#20	Potential sources of influence of perceived influence on study conduct and conclusions; how these were managed	N/A
Funding	#21	Sources of funding and other support; role of funders in data collection, interpretation and reporting	1

The SRQR checklist is distributed with permission of Wolters Kluwer © 2014 by the Association of American Medical Colleges. This checklist was completed on 21. October 2019 using https://www.goodreports.org/, a tool made by the EQUATOR Network in collaboration with Penelope.ai

For peer review only

BMJ Open

Discussing surgical innovation with patients: A qualitative study of surgeons' and governance representatives' views

Journal:	*BMJ Open*
Manuscript ID	bmjopen-2019-035251.R1
Article Type:	Original research
Date Submitted by the Author:	14-Apr-2020
Complete List of Authors:	Zahra, Jesmond; University of Bristol, National Institute for Health Research Bristol Biomedical Research Centre Surgical Innovation Theme, Bristol Centre for Surgical Research, Bristol Medical School: Population Health Sciences, University of Bristol<br>Paramasivan, Sangeetha ; University of Bristol, National Institute for Health Research Bristol Biomedical Research Centre Surgical Innovation Theme, Bristol Centre for Surgical Research, Bristol Medical School: Population Health Sciences, University of Bristol<br>Blencowe, Natalie; University of Bristol, National Institute for Health Research Bristol Biomedical Research Centre Surgical Innovation Theme, Bristol Centre for Surgical Research, Bristol Medical School: Population Health Sciences, University of Bristol; University Hospitals Bristol NHS Foundation Trust, Division of Surgery<br>Cousins, Sian; University of Bristol, National Institute for Health Research Bristol Biomedical Research Centre Surgical Innovation Theme, Bristol Centre for Surgical Research, Bristol Medical School: Population Health Sciences, University of Bristol<br>Avery, Kerry; University of Bristol, National Institute for Health Research Bristol Biomedical Research Centre Surgical Innovation Theme, Bristol Centre for Surgical Research, Bristol Medical School: Population Health Sciences, University of Bristol<br>Mathews, Johnny; University of Bristol, National Institute for Health Research Bristol Biomedical Research Centre Surgical Innovation Theme, Bristol Centre for Surgical Research, Bristol Medical School: Population Health Sciences, University of Bristol<br>Main, Barry; University of Bristol, National Institute for Health Research Bristol Biomedical Research Centre Surgical Innovation Theme, Bristol Centre for Surgical Research, Bristol Medical School: Population Health Sciences, University of Bristol; University Hospitals Bristol NHS Foundation Trust<br>McNair, Angus; University of Bristol, National Institute for Health Research Bristol Biomedical Research Centre Surgical Innovation Theme, Bristol Centre for Surgical Research, Bristol Medical School: Population Health Sciences, University of Bristol; North Bristol NHS Trust, GI Surgery<br>Hinchliffe, Robert; University of Bristol, National Institute for Health Research Bristol Biomedical Research Centre Surgical Innovation Theme, Bristol Centre for Surgical Research, Bristol Medical School: Population Health Sciences, University of Bristol; North Bristol NHS Trust, Vascular Services<br>Blazeby, Jane; University of Bristol, National Institute for Health

	Research Bristol Biomedical Research Centre Surgical Innovation Theme, Bristol Centre for Surgical Research, Bristol Medical School: Population Health Sciences, University of Bristol; University Hospitals Bristol NHS Foundation Trust<br>Elliott, Daisy; University of Bristol, National Institute for Health Research Bristol Biomedical Research Centre Surgical Innovation Theme, Bristol Centre for Surgical Research, Bristol Medical School: Population Health Sciences, University of Bristol

**Primary Subject Heading**:	Surgery
Secondary Subject Heading:	Communication
Keywords:	SURGERY, Health policy < HEALTH SERVICES ADMINISTRATION & MANAGEMENT, QUALITATIVE RESEARCH

SCHOLARONE™ Manuscripts

I, the Submitting Author has the right to grant and does grant on behalf of all authors of the Work (as defined in the below author licence), an exclusive licence and/or a non-exclusive licence for contributions from authors who are: i) UK Crown employees; ii) where BMJ has agreed a CC-BY licence shall apply, and/or iii) in accordance with the terms applicable for US Federal Government officers or employees acting as part of their official duties; on a worldwide, perpetual, irrevocable, royalty-free basis to BMJ Publishing Group Ltd ("BMJ") its licensees and where the relevant Journal is co-owned by BMJ to the co-owners of the Journal, to publish the Work in this journal and any other BMJ products and to exploit all rights, as set out in our licence.

The Submitting Author accepts and understands that any supply made under these terms is made by BMJ to the Submitting Author unless you are acting as an employee on behalf of your employer or a postgraduate student of an affiliated institution which is paying any applicable article publishing charge ("APC") for Open Access articles. Where the Submitting Author wishes to make the Work available on an Open Access basis (and intends to pay the relevant APC), the terms of reuse of such Open Access shall be governed by a Creative Commons licence – details of these licences and which Creative Commons licence will apply to this Work are set out in our licence referred to above.

Other than as permitted in any relevant BMJ Author's Self Archiving Policies, I confirm this Work has not been accepted for publication elsewhere, is not being considered for publication elsewhere and does not duplicate material already published. I confirm all authors consent to publication of this Work and authorise the granting of this licence.

For peer review only

Discussing surgical innovation with patients: A qualitative study of surgeons' and governance representatives' views

Submission category: Original article

J Zahra[1], S Paramasivan[1], N.S Blencowe[1,3], S Cousins[1], K Avery[1], J Mathews[1,2], B Main [1,3], A McNair [1,2], R Hinchliffe[1,2], J.M Blazeby[1,3], D Elliott[1]

[1]National Institute for Health Research Bristol Biomedical Research Centre Surgical Innovation Theme, Centre for Surgical Research, Population Health Sciences, Bristol Medical School, University of Bristol, 39 Whatley Road, Clifton, Bristol, UK. BS8 2PS;

[2]North Bristol NHS Trust, Bristol, UK. BS10 5NB.

[3]University Hospitals Bristol NHS Foundation Trust, Bristol, UK. BS2 8HW

Running title: Informed consent for innovative surgery

Keywords: consent, surgery, information provision, innovation, qualitative,

Corresponding author details: Daisy Elliott [daisy.elliott@bristol.ac.uk]

Funding statement: This study was supported by the NIHR Biomedical Research Centre at University Hospitals Bristol NHS Foundation Trust and the University of Bristol, the MRC ConDuCT-II (Collaboration and innovation for Difficult and Complex randomised controlled Trials In Invasive procedures) Hub for Trials Methodology Research (MR/K025643/1). The views expressed in this publication are those of the author(s) and not necessarily those of the NHS, the National Institute for Health Research or the Department of Health and Social Care. BM is a NIHR Clinical Lecturer, AM is funded by a Clinician Scientist Fellowship (NIHR-CS-2017-17-010) from the National Institute for Health Research. NB is funded by an MRC Clinician Scientist Award.

Word count: 4380

For peer review only - http://bmjopen.bmj.com/site/about/guidelines.xhtml

Abstract

Objectives

Little is known about how innovative surgical procedures are introduced and discussed with patients. This qualitative study aimed to explore perspectives on information provision and consent prior to innovative surgical procedures.

Design

Qualitative study involving semi-structured interviews. Interviews were audio recorded, transcribed and analysed thematically.

Participants

42 interviews were conducted (26 surgeons and 16 governance representatives).

Setting

Surgeons and governance representatives recruited from various surgical specialities and National Health Service Trusts across England, United Kingdom.

Results

Participants stated that if a procedure was innovative, patients should be provided with additional information extending beyond that given during routine surgical consultations. However, difficulty defining innovation had implications for whether patients were informed about novel components of surgery and how the procedure was introduced (i.e. as part of a research study, trust approval or in routine clinical practice). Furthermore, data suggests surgeons found it difficult to establish what information is essential and how much detail is sufficient, and governance surrounding written and verbal information provision differed between NHS trusts. Generally, surgeons believed patients held a view that 'new' was best and reported that managing these expectations could be difficult, particularly if patient views aligned with their own.

Conclusions

This study highlights the challenges of information provision and obtaining informed consent in the context of innovative surgery, including establishing if and how a procedure is truly innovative, determining the key information to discuss with patients, ensuring information provision is objective and balanced, and managing patient expectations and preferences. This suggests that surgeons may require support and training to discussing novel procedures with patients. Further work should

For peer review only - http://bmjopen.bmj.com/site/about/guidelines.xhtml

capture consultations where new procedures are discussed with patients and patients' views of these information exchanges.

For peer review only

For peer review only - http://bmjopen.bmj.com/site/about/guidelines.xhtml

Summary box – strengths and limitations

- This is the first qualitative study to conduct in-depth, semi structured interviews to understand current practice for information provision and informed consent for innovative surgical procedures in the United Kingdom.
- Interviews with governance representatives enabled us to understand what policies were in place for the introduction of new procedures, whilst surgeons provided insights into how they introduced new procedures and discussed these with patients.
- Purposeful sampling ensured that participants were recruited from a range of geographical locations and trust types, and surgeons were from different specialities with varying experiences of innovation.
- Audio-recording appointments where clinicians discuss innovative surgeries and patients decide whether or not to undergo the procedure would strengthen our understanding of interactions between clinicians and patients.
- Further work should capture patients' views on information provision in this context, so that recommendations can be made to improve transparency and communication.

For peer review only

For peer review only - http://bmjopen.bmj.com/site/about/guidelines.xhtml

Introduction

Informed consent is fundamental to patient autonomy and represents a cornerstone of modern healthcare provision. Until recently, the Bolam ruling meant that a doctor's duty to inform patients prior to surgery was used to judge on whether they had acted in line with the view of a responsible body of medical opinion (1, 2). The 2015 Montgomery ruling(3) redefined the standard of informed consent in the United Kingdom (UK) and represented a shift from a paternalistic model of consent to a more patient-centred approach(4, 5) – meaning that a patient should be told whatever they want to know, not what the doctor thinks they should be told(6).

Recently, important questions have been raised surrounding informed consent for innovative surgical procedures(7, 8). An inquest into the death of the first patient in the UK to undergo robotically assisted heart surgery found the patient had not been fully informed about the comparative risk of robotic versus conventional open surgery(8). In addition, a summary of invited reviews published by the Royal College of Surgeons of England (RCSEng) raised further concerns(7). The review highlighted issues surrounding surgeons' ability to accurately quantify risk as reported in published literature, whether surgical experience is being sufficiently disclosed and effectively communicated to patients, and the overall quality of patients-clinician discussions(7). The RESEng subsequently published guidelines for the development, implementation and dissemination of surgical innovation, which contains guidance on informed consent (9). This suggests, based on findings of a review by Broekman et al (9), that patients should be informed of the innovative nature of the procedure, surgeons' experience with the procedure and the learning curve, the risk and benefits – including unknown or unforeseeable risks or outcomes, the evidence or lack thereof, and alternatives to the innovative procedure.

Reviews and commentaries have highlighted potential issues around informed consent for innovative surgical procedures, including patients' and surgeons' beliefs that new treatments constitute improved treatment options or result in better outcomes (10, 11). However, little is known about current practice. Surveys that have been conducted in the United States suggest that information varies considerably in content and quality. Reitsma and Moreno (2002) reported that of twenty-one surgeons, 75% verbally informed patients about the innovative nature of the procedure but only 33% provided written information (12). Char and colleagues (2013), in a scenario based survey carried out with 85 surgeons and 383 patients, found differences between what surgeons thought patients wanted to know about innovative procedures and what patients reported they wanted to know. For instance, compared with surgeons, patients placed more importance on nearly all types of information, particularly volumes and outcomes (13).

For peer review only - http://bmjopen.bmj.com/site/about/guidelines.xhtml

Whilst a handful of qualitative studies have been conducted exploring how innovative procedures have been introduced (14) (15) (16) (17), none have specifically looked at information provision and informed consent. Moreover, these studies were conducted in Australia and Canada and findings may not be generalisable to other countries and different healthcare systems(18). New invasive procedures may be introduced in the context of formal research studies or via local hospital policies (19). The National Institute for Health and Care Excellence (NICE) recommends that local NHS organisations have appropriate governance structures in place to review, approve and monitor the introduction of new invasive procedures (20). The current study sought to explore surgeons' and governance representatives' views of information provision and informed consent for the introduction of new invasive procedures in the United Kingdom (UK).

Methods

Patient and public involvement

Public and patient involvement was not conducted as part of the current study. However, the current findings have been discussed with a recently established PPI group, which has led to further work which specifically aims to explore patient views on surgical innovation.**Design**

One-to-one in-depth semi-structured interviews were undertaken. Interviews were considered to be the most appropriate method for this study because they provided the opportunity to encourage participants to think carefully about their own experiences (21), enable the interviewer to respond and follow up on issues raised by the interviewee (22), and that some participants may feel intimidated at the prospect of discussing their experiences within a focus group setting(23). Ethical approval was granted by the University of Bristol Faculty of Health Sciences Research Ethics Committee. The study is reported in line with the Standards for Reporting Qualitative Research (SRQR; Additional File 1) (24).

Sampling and recruitment

Participants were recruited between November 2017 and October 2018 using a combination of purposive (25)and snowball sampling techniques (23). Governance representatives (defined as those involved in regulating the introduction of new/modified procedures and/or devices at trust or national levels) were included to understand what processes were in place for the introduction of new procedures (17). Surgeons were recruited to explore experiences of introducing new/modified procedures or devices into clinical practice. As there is no standardised definition for innovation and it is often a continuum from standard practice through to true innovation (14, 26, 27), and because

For peer review only - http://bmjopen.bmj.com/site/about/guidelines.xhtml

surgical innovation is unlikely to involve a single discrete development (28, 29), participants were asked to reflect what the term meant to them and describe procedures that they deemed to be innovative.

Governance representatives were identified through websites (e.g. National Health Service (NHS) Trusts websites) and NHS policy documents (e.g. new invasive procedure/devices policies). An initial sample of surgeons were identified by academic surgeons working within the Centre for Surgical Research, University of Bristol, UK. Subsequent surgeons were identified via snowball sampling(23), in which interviewees recommended others who may be willing to take part. Sampling was regularly reviewed to ensure surgeons from different specialities and geographical locations, with varying experiences of innovation (e.g. minor modifications vs first in human procedures), were recruited to capture a diversity of perspectives (30, 31). For governance representatives, maximum variation was sought in relation to different roles (e.g. new procedures committee members, medical directors), geographical locations, trust types, and different surgical specialities.

Potential participants were approached via email and up to three invitation emails were sent before contact attempts ceased. In total, 83 potential participants were invited to take part (38 surgeons and 45 governance representatives). Study recruitment ceased when the qualitative team agreed that theoretical saturation had been achieved (where no new themes were forthcoming from three consecutive interviews)(32, 33).

Data collection

All participants received a study information leaflet and provided written consent before taking part. Separate topic guides were developed for governance representatives and surgeons. All interview schedules contained core topics of interest (e.g. 'How would you define innovation?', 'What does informed consent mean to you?')(34, 35), although these were flexible to allow participants to shape the direction of conversations(36, 37). Governance representatives were asked how applications for new invasive procedures were processed and how these procedures then integrated into practice, whereas surgeons were asked about their experiences of introducing procedures and what patients had been told. An example of each interview schedule is provided in the supplementary files (Additional files 2 and 3).

The interviewer began the discussion with more general questions to build rapport (38) such as the participants' background and general views of defining innovation. The interviewee's comments were restated and incorporated into further questions to check with the interviewer that they had understood correctly(36). In order to gain more detailed and comprehensive understanding of the

For peer review only - http://bmjopen.bmj.com/site/about/guidelines.xhtml

descriptions given by participants, they were asked to elaborate their comments with explanations and examples (such as, 'Why do you feel this was?')(36), or fillers (such as 'mmm' or 'yeah') were used by the interviewer to show the participant this was something they were interested in and to encourage them to continue (39). Towards the end of the interview, participants were given an opportunity to raise issues that were important to them that had not already been covered (38).

Topic guides were developed iteratively during the study period, in consideration of emerging insights from interviews and better ways of phrasing questions(34, 40). For instance, interviewers (non-clinical academics) used more prompts to encourage the surgeons to describe procedures in a non-technical manner (36). Topic guides were initially very long so were reduced to include more 'mapping' questions that addressed the core components of interest (such as, 'I understand you've conceptualised a new procedure. Can you talk me through this?'). Additional prompts were added from emerging findings (i.e. colleagues' reactions to procedures and written information). With permission, interviews were audio recorded using an encrypted digital dictaphone.

All interviews were audio recorded expect one where the interviewee requested not to be recorded and therefore written notes were taken. All interviews were conducted by one of two trained and experienced qualitative researchers (JZ or DE). JZ is a male Senior Research Associate with a background in public health research. DE is a female Research Fellow with experience working on surgical trials. She is also a member of the QuinteT research group, which uses qualitative research methods to optimise recruitment and informed consent to randomised controlled trials (RCTs). JZ and DE have several years of experience conducting qualitative research, and each has a degree in Psychology and a PhD in health-related research.

Data analysis

Interview recordings were transcribed and de-identified so that names and identifiable information were removed. Thematic analysis was undertaken using the constant comparison technique of grounded theory (41, 42), which involved using the inductive identification of codes from the data to generate new hypotheses about phenomena that are derived or grounded in the data (43). Its central principle is of constant comparison, where new findings are systematically compared with existing data so that similarities and differences can be identified and emerging theories refined through the ongoing assimilation of data (41, 44).

Data were imported into a data management program (NVivo version 11) to organise the data systematically, allow for quick searching and refining of codes and categories, increase the

For peer review only - http://bmjopen.bmj.com/site/about/guidelines.xhtml

transparency of the analysis process and enable team comparisons of coding (32, 45). First, analysts (JZ, DE & SP) read and re-read transcripts before independently coding a sample of five transcripts to begin familiarising themselves with the data (32, 46). Interesting features were coded, whereby a segment of data was assigned a code (40), and on-going potential ideas were noted at every stage of analysis(47). Data coding was compared to ensure consistency and rigour in the findings(48), whereby differences were discussed thoroughly until a consensus was met and an initial coding frame was developed. The coding frame was then independently applied to three transcripts by JZ, DE and SP. High consistency between analysts indicated the coding frame appropriately captured the data. JZ then applied the coding frame to the remaining transcripts, adding additional codes if important data were missed by the original coding frame. To form preliminary themes and subthemes, codes were collated into hierarchical groups(49). Emerging findings were regularly discussed with JMB and NB (both academic surgeons), with reference to the raw transcripts, to ensure they fully encapsulated the meaning of the data(48). Finally, a descriptive account of the themes and subthemes, which included representative quotes, was developed.

Results

Participants

Of the 83 participants that were approached, 42 participants were recruited (those who did not participate were unable to find time for an interview or did not respond to study invites). The final sample included 26 surgeons and 16 governance representatives from across 23 NHS Trusts (see Table 1). Trusts varied in geographical location, type/size (i.e. teaching, specialist, large, multi-centre) and foundation status. Interviews lasted an average of 43 minutes (range 22 – 112 minutes, standard deviation 17 minutes). Governance representatives' roles within trusts varied (e.g. medical director, director for quality improvement, head of governance), however, all played a role in regulating the introduction of new surgical procedures and/or devices. At the time of interview, all but one (retired) surgeon worked as a consultant within the NHS. Interviews were conducted over the phone (55%) or face-to-face at participants' place of work or at a university.

Table 1. Participants recruited

	ID	Position	Site	Gender
Governance representatives (n=16)	2	Chair Clinical Effectiveness Group	Trust 1	Male
	3	Trust NICE manager	Trust 1	Male
	7	Programme Director	NICE	Male
	15	Clinical Effectiveness Group member	Trust 1	Female
	24	Director for Quality Improvement	Trust 9	Male
	29	Director of Research and Informatics	Trust 5	Male
	39	Medical Device Safety officer	Trust 4	Male

For peer review only - http://bmjopen.bmj.com/site/about/guidelines.xhtml

	40	Clinical Effectiveness Coordinator	Trust 17	Male
	41	Associate Medical Director	Trust 20	Male
	44	Chair Clinical Effectiveness Sub Group	Trust 19	Female
	47	Head of Governance and Legal Services	Trust 18	Female
	48	Clinical Effectiveness Lead	Trust 24	Male
	14	Medical director	Trust 10	Male
	17	Associate Medical Director	Trust 15	Male
	26	Interim Medical Director	Trust 6	Male
	37	Medical director	Trust 11	Male
Surgeons (n=26)	1	Professor of Cardiac Surgery	Trust 1	Male
	4	General & Upper Gastrointestinal Surgeon	Trust 12	Male
	5	Thoracic Surgeon	Trust 1	Male
	6	Hepatobiliary, pancreatic & general Surgeon	Trust 1	Male
	8	Ophthalmic Surgeon	Trust 10	Male
	9	Professor of Gastrointestinal Surgery	Trust 3	Male
	11	Hepatobiliary & general Surgeon	Trust 1	Male
	13	Colorectal Surgeon	Trust 1	Male
	16	Urologist	Trust 2	Male
	18	General Surgeon	Trust 3	Male
	19	Thoracic Surgeon	Trust 1	Male
	22	Thoracic Surgeon	Trust 7	Male
	23	Colorectal Surgeon	Trust 2	Female
	25	Thoracic Surgeon	Trust 14	Male
	28	General Thoracic Surgery	Trust 5	Male
	30	Professor of Surgery	International	Male
	31	Colorectal Surgeon	Trust 8	Male
	33	Cardiac Surgeon	Retired	Male
	34	Professor of Urology	Trust 13	Male
	35	Hepatobiliary, pancreatic & general Surgeon	Trust 1	Male
	36	Oncoplastic Breast Surgeon	Trust 4	Female
	42	Neurosurgeon	Trust 13	Male
	43	Orthopaedic Surgeon	Trust 2	Male
	45	Spinal Surgeon	Trust 21	Male
	46	Trauma & Orthopaedic Surgeon	Trust 22	Male
	49	Orthopaedic Surgeon	Trust 2	Male

For peer review only

Analysis

Findings related to informed consent are presented under four main themes: *difficulty defining innovation*, d*iffering views on what – and how - patients should be told, the challenges of discussing uncertainty,* and *managing patient expectations*. All quotations are followed by participants' unique identification number, gender, surgical speciality and trust identification number.

Difficulty defining innovation

For peer review only - http://bmjopen.bmj.com/site/about/guidelines.xhtml

Surgeons and governance representatives found it challenging to define surgical innovation and considered the term ‘innovation’ to encompass a spectrum of changes to surgical healthcare. Examples ranged from changing patient pathways through to first in human procedures. Consequently many felt innovation should be viewed as a continuum, rather than a binary concept:

> *“Well there’s a spectrum isn’t there, I mean it’s difficult to define. So something that is innovative to me is something that is new, it’s a new way of looking at it, it’s a slightly different paradigm, a different way of thinking. And an adaptation is just a sort of a natural evolution of something that’s already pre-existing, I suppose that’s how I would define them, but clearly it’s a spectrum isn’t it?” (Surgeon 18, General surgery, Male, Trust 3)*

There was variation in how the procedure had been introduced in participants’ hospitals. Within this, surgeons’ own experiences included requesting approval from their trust’s new procedure committee, conducting the procedure in the context of a research study or deeming that approval was not necessary as the procedure was a variation of an existing one (from either judgement of the surgeon or confirmation from a committee). Table 2 highlights that how the challenges of defining innovation, alongside the required technical knowledge of the procedure, could cause uncertainty as to what governance was required:

Table 2: Participants' experiences of governance for new procedures

	**Example quote**
**Procedure considered and approved by trust’s new procedure committee**	*“There’s one surgeon in [European country] who described the procedure, and the surgeon in [Asian country] who had done some of these procedures, but not really clear on how they’d done it. I contacted the [European] surgeon. He gave me some pointers, so I discussed it with my colleagues, and of course put it through the governance processes as a novel and a new procedure. And then we did it.” (Surgeon 31, Colorectal, Male, Trust 8)*
**Approval from trust’s new procedure committee deemed unnecessary as felt to be a variation of established procedure**	*“We asked about it to the new procedures committee, but they said well it’s basically [variation of established procedure] isn’t it. The technicalities of it are lost on non-thoracic surgeons really, which is good.” (Surgeon 31, General surgery, Male, Trust 8)* *“There’s a new technique that’s probably done by no more than 9 or 10 surgeons in the country. Again, it’s a new technique, it’s only been reported in the last 3 or 4 years. […] Because it was under the umbrella of [broader procedure], I didn’t have to go through any, so within the hospital there’s a board about new techniques, you have to sort of, clinical effectiveness group, you have to have that approved. But, because this was a variation, we didn’t need that approval.” (Surgeon 22, Male, Orthopedics, Trust 7)*
**Procedure evaluated in the context of a research study**	*“We were looking [procedure], and so that was in the guise of a randomised double-blind trial that we put through Ethics. So that would be innovative.*

For peer review only - http://bmjopen.bmj.com/site/about/guidelines.xhtml

	We've got an Ethics Committee, and as part of that if you were going to be doing something really brand new that would have to be conducted as part of a trial, so, but when there's a blur between doing something slightly different then no I don't think it's the place for that [...] I can't think of anything where they've been involved in, you know, anything that we're doing." (Surgeon 46, Male, Orthopedics, Trust 22)

"So, I'm not convinced that every innovative thing in the trust goes through the committee, because a lot of it depends on the diligence of the people who are introducing new things. So, it's not a bad process but it's just whether it's comprehensive enough, I guess." (Surgeon 1, Male, Neurology, Trust 1)

Although participants initially stated that patients should be informed about new procedures, some drew upon experiences that contradicted this as they reflected upon the complexity of defining innovation:

> *"I think absolutely they should be informed, I think that's a tricky one actually… when would I inform a patient about a new instrument I was using? So we don't- if- if you're not trialling the instrument to see if it's safe or more effective or less effective, then I don't think they need to be informed." (Surgeon 4, Male, General and upper gastrointestinal, Trust 12)*

> *"We kind of created our own version of how you do that operation […] So, it's not particularly been described before by surgeons and it's not the world's most different, but it's got a few little innovative techniques. So, that was an operation we designed that was new […] I don't even explain how that's different to [standard procedure] because I think it's far too technically nuanced for a patient to understand… We view it as just a variation of [standard procedure]." (Surgeon 22, Thoracic, Male, Trust 7)*

Several governance representatives also described instances where they had become aware of patients not being fully informed about new procedures and emphasised the need for improved information provision:

> *"So we then audit the first case to make sure that the clinician has been up front with the individual about the fact that this is the first time that this particular procedure has been undertaken…if they haven't, there's been a couple of occasions when they haven't, we tell them off and say do better next time and we*

For peer review only - http://bmjopen.bmj.com/site/about/guidelines.xhtml

audit the second case or whatever, so we're confident they've got it right." (Governance representative 29, Director of research, Male, Trust 5)

"We have had a couple of near misses … one was a surgeon inviting another surgeon from another hospital to come and show him how to do a procedure, that person did not have an honorary contract with the Trust therefore shouldn't have been operating and nobody knew that that was occurring. And we have had a rep [industry representative] coming into theatres to bring some samples of a new piece of equipment and most certainly patients weren't consented that it was going to be used on them … So I think there is increasing awareness now, certainly when business cases are presented, they do need to follow this process if that's what's going to happen. However there is also, can I put my hand on my heart and say actually the surgeon's not going to invite a rep into theatre tomorrow? I probably couldn't, but I do know that the theatre staff feel more empowered to be able challenge that behaviour, so actually if I knew that somebody was going to use a piece of equipment they've never used before I would probably be asking the question as to why." (Surgeon 9, Upper gastrointestinal, Male, Trust 3)

Differing views on what – and how - patients should be told

There was consensus from all participants that if a procedure was deemed innovative, patients should be provided with additional information extending beyond that given during routine surgical consultations. Within this, the potential risks and benefits, alternative treatment options, and the novel status of the procedure were universally regarded as essential information that should be discussed.

"We tell them that it's a new procedure and previously we've told them how many we've done prior to this" (Surgeon 5, Thoracic, Male, Trust 5)

"You have to give them options, you have to let them know what the evidence base is." (Surgeon 46, Trauma & Orthopaedic, Male, Trust 22)

There was variation in views as to whether other information - such as the evidence base (or lack thereof), operative experience, training, safety precautions implemented in theatre and national guidance - such be disclosed. Taken together, there appeared to be uncertainty at precisely how much to tell patients:

"Sometimes I tell them too much or tell them... I mean, the latest, er, ruling of Montgomery as I understand it is that they need to be told what they would want to know. Bloody hell. But then you've got to judge what they want to know and you've

For peer review only - http://bmjopen.bmj.com/site/about/guidelines.xhtml

got to have some kind of communication with them and reasonably understand what it is they want to know." (Surgeon 31, Colorectal, Male, Trust 8)

"There is a difficult balance I think, and I'm not sure we've got it [consent] right yet actually." (P7)

Many interviewees felt strongly that patients should be provided with verbal (i.e. discussions during consultation) *and* written information (i.e. via a patient information leaflet (PIL)) when being offered innovative surgery. In line with this, most governance representatives reported that innovative procedures could only be introduced if an appropriate PIL had been developed (although there was variation as to whether this was reviewed by the committee).

"We will insist that there is a patient guide…an information sheet for the patient…It [PIL] will talk about the relative inexperience of the team, saying that this is new to us, but that we're doing it under conditions where we've got an expert coming to help us, we've been to appropriate training, it's been approved by the organisation as something that we think will be safe, but that it is a new way of doing things." (Governance representative 24, Director for Quality Improvement, Male, Trust 9)

However, other trusts had no mandatory requirements for written information or did not review written documentation as part of the application to introduce a new procedure:

"Obviously in situations in which the technique might be new then that's (PIL) not something I've specifically addressed but probably should do." (Governance Representative 48, Clinical Effectiveness Lead, Male, Trust 24)

"I definitely think they should know it's a new procedure. The way we probably tell (patients) is only in the consent process verbally, I don't recall seeing that in any of our written leaflets." (Governance representative 47, Head of governance, Female, Trust 18)

The challenges of discussing uncertainty

As previously discussed, surgeons felt a need to deliver additional information during consultations for innovative surgery. However, data suggest that some surgeons may find this challenging:

"it's just trying to get across not only this fact that this is what it is, but the fact that it's newer than something else which as a standard we use. That's quite difficult trying to get that across, and some patients will understand better than others." (Surgeon 42, Neurosurgery, Male, Trust 13)

For peer review only - http://bmjopen.bmj.com/site/about/guidelines.xhtml

At a practical level, surgeons reported finding it difficult to establish what information is essential and how much detail is enough. These issues may be further compounded when faced with time constraints, which limit surgeons' ability to have long and/or multiple discussions with patients:

> *"We don't have much time …I'm finding that I'm over talking far too much information, but I need to make them aware that they're essentially guinea pigs." (Surgeon 23, Colorectal, Female, Trust 2)*

Delivering information to a variety of patients who may differ in their ability to understand complex - and potentially very technical - information was a further challenge. To help overcome this, some surgeons felt it was important to simplify technical information and, when possible, provide alterative mediums of information to aid decision making:

> *"It's [surgical procedure] such a complex thing to be explaining to people so you have to try and bring it down to simplistic terms but at the same time give it in a way that actually they can process the salient points of the information." (Surgeon 46, Trauma & Orthopaedic, Male, Trust 22)*

> *"I think one of the most important things about when you're making this change is that you give people different forms of access, and of course now it's easy. There are YouTube videos of stuff." (Surgeon 43, Orthopaedic, Male, Trust 2)*

Surgeons acknowledged that innovative surgery was associated with uncertainties, including a lack of knowledge surrounding risks, benefits and long-term outcomes. Some felt that discussing uncertainties may conflict with the professional norms within surgery:

> *"This is what surgeons find hard. You have to say, "I don't know," and we hate that. But actually, we should say it more often. We should say it more often." (Surgeon 4, General and upper gastrointestinal, Male, Trust 12)*

As a result, when faced with uncertainty surrounding an innovative surgery, surgeons sometimes appeared to disclose their personal opinions during patient consultations:

> *"They were told that it was a new technique, that we weren't sure what the long-term outcomes were going to be but, we thought that it was better than traditional surgery." (Surgeon 16, Urology, Male, Trust 2)*

On the other hand, some expressed a desire to remain impartial and stressed the importance of providing balanced information. Several interviewees described using strategies to help ensure they remained as objective as possible during consultations. Examples discussed included adhering to scripted information and only discussing innovative surgery if a patient specifically requested an alternative to conventional approaches:

For peer review only - http://bmjopen.bmj.com/site/about/guidelines.xhtml
For peer review only

"You can word a new procedure, this is amazing, fantastic, amazing, it's phenomenal and there's no risks to it, and all the patients will say sure, yeah sign me up. Whereas, this is what we're trying to look for, this is the benefits, these are the potential risks, so on and so forth. Then it becomes much more objective." (Surgeon 25, Thoracic, Male, Trust 14)

Of note however, the language used to describe conventional surgical approaches during interviews highlights the potential for surgeons – albeit unintentionally – to influence treatment choice. For instance, terms such as 'old fashioned' and 'traditional' were used by some when describing how they approached patient information provision:

"I'll tell them, "We're going to try and do it like this. Is that okay? Do you have any objection? The alternative is we're going to do it the old-fashioned way, which is make a big cut in your- on your stomach wall" (Surgeon 4, General & Upper Gastrointestinal Surgeon, Male Trust 12)

Managing patient expectations

Surgeons felt that patients often had preferences for new treatments and expected these to deliver better outcomes. For instance those with experience of introducing robotically assisted surgery commonly reported that patients conveyed a strong preference for this approach. Consistent with this, one interviewee felt that patient demand for robotically assisted surgery was a driving factor for hospitals to invest in robotics.

"Patients took to it [robotically assisted surgery] very well. I thought they might be a bit nervous about it, you know the robot taking over or going crazy…patients like the idea of technology. They equate the robot to being an advanced, superior treatment." (Surgeon 31, Colorectal, Male, Trust 8)

"Patients wanted to have robotic surgery, and if you didn't have a robot, the patients would just go to a neighbouring hospital that did. So, it's really patient driven, so if you wanted to be a centre you have to have a robot." (Surgeon 16, Urology, Male, Trust 2)

In general, interviewees felt patients held a misconception that 'new' surgical procedures and technologies are better than those normally provided. Some surgeons reported that patients had actively sought them out in order to receive innovative surgery:

"They tend to have sought you out, because they want something different than the standard. So they're kind of are willing to accept uncertainty and risk because they've already sought out that uncertainty and risk, if you know what I mean." (Surgeon 9, Upper gastrointestinal, Male, Trust 3)

For peer review only - http://bmjopen.bmj.com/site/about/guidelines.xhtml

Several surgeons were concerned that patients did not understand that innovative procedures may carry uncertainties in terms of risk and benefit. Managing patients' preferences, particularly if they align with the personal preferences, appeared challenging for some:

> *"When does a patient turn around and say, 'Well I'll have the old one'? And the answer is never, and that's difficult, because they are immediately signing themselves up for an experiment. And how you describe that to patients is really difficult, because you've got all of your intrinsic biases yourself. Okay, go on then." (Surgeon 43, Orthopaedic, Male, Trust 2)*

> *"I don't think I've had anyone say, 'no, thank you'. I think most of them have gone, 'I don't mind being a guinea pig'. They always use that phrase (laughter). Makes me feel a little bit uncomfortable." (Surgeon 36, Breast, Female, Trust 4)*

Discussion

This study provides novel insights into surgeons' and governance representatives' views on information provision in the context of innovative surgical procedures and represents a significant contribution to the limited empirical literature. Results highlight inconsistencies in how NHS trusts govern how patients should be informed about innovative surgical procedures. Furthermore, interviews with surgeons suggest a lack of consistency in amount and quality of the provision of verbal and written information prior to delivery of innovative surgical treatment. Data indicate a heavy reliance on surgeons to decide what and how information is communicated to patients within some trusts. This conflicts with the recommendations published by the RCSEng, which suggest oversight committees should discuss information provision and consent with surgeons prior to approvals being granted (9). Collectively, our results stress the need for better oversight and regulation of information provision in the context of innovative surgery. Without improved standards of information provision, patients' informed consent is potentially compromised. There is, however, particular complexity in understanding what innovation is, and when it might occur, that makes establishing these standards difficult.

Consistent with previous research, our findings suggest that surgeons may struggle to clearly define surgical innovation(50), and this may impact upon their approach to patient information provision and consent. It can represent a continuum from standard practice through to true innovation. Rogers et al (14) demonstrated that surgeons held no uniform view of surgical innovation, and showed the lack of agreement on the distinction between innovation and research. Our study highlights the implications of ambiguity – surgeons may use their own judgement as to what is innovative and therefore not discuss novel components of an operation with patients. In addition,

For peer review only - http://bmjopen.bmj.com/site/about/guidelines.xhtml

procedures that were deemed innovative were sometimes introduced as part of routine clinical practice. This meant that patient information provided was at the discretion of the surgeon, rather than an ethics review board and no peer review of the process. Birchley et al have suggested that instead of focusing on whether something is 'innovative' or not, the potential risks and the ethical appropriateness of modifying surgical practice should be considered to ensure the safe translation of surgical innovation into clinical practice(29). Work is ongoing to better conceptualise what innovation is and determine when it is happening (51) - although in the meantime we rely on surgeons' views on what is innovative and how that influences their practice of information provision.

Prior research has shown patients do not always feel fully informed about the different treatment options available to them(52, 53). It is likely that the added complexity associated with innovative treatments (e.g. unknown unknown risks and benefits, lack of long-term data, limited surgeon experience of this technique) makes discussing treatments options particularly challenging for surgeons. This has an impact on the process of information provision within the context of gaining informed consent to undergo the procedure. As many innovations are undertaken within the context of clinical practice and not research, then the information provision is at the discretion of the surgeon. Study participants recognised the importance of communicating the risks and benefits, treatment alternatives, and novel status of a procedure to patients. However, it was less common for interviewees to identify prior training, experience with undertaking the procedure, and the evidence base supporting the innovation as important components of consent in information provision. These elements of information provision are recognised as key to gain informed consent (2, 9). The variation observed in this study suggests there is a lack of clarity in guidance for surgeons on what should be disclosed during the content of informed consent discussions in the context of innovative surgery, meaning that some patients are likely to be better informed than others when making treatment decisions. This is a concern given that information provision within the context of informed consent has been linked to patient satisfaction and rates of litigation following surgery(54-56).

Aligning with previous research, our data suggests surgeons may not disclose their personal experience of performing a procedure to patients and this is important to patients(57). In this study few surgeons identified, or reported discussing, surgical experience and/or training as a component of information provision. While we cannot conclude from the current data, the disclosure of this information could lead to conversations regarding the surgical learning curve. Such discussions could help emphasise to patients that a procedure is novel and there are potential uncertainties in terms

For peer review only - http://bmjopen.bmj.com/site/about/guidelines.xhtml
For peer review only

of risks and benefits. Training programmes have been developed to enhance informed consent in RCTs(58, 59), although none have focused on innovative surgical procedures. Our study suggests that surgeons may require support and training to raise awareness of the challenges of discussing novel procedures and provide support to overcome these.

To the best of our knowledge, this study is the first to explore healthcare professionals' views of what information should be shared with patients during informed consent consultations in the context of innovative surgery. An important strength of the study is the sample of both surgeons and governance representatives, recruited from various surgical specialities, trust types and geographical locations. This allowed us to explore information provisioned consent from two different perspectives, and provided important insight into the role trust governance plays in patient information provision and consent. Nonetheless, there are potential limitations to the study that should be acknowledged. We cannot conclude exactly what information is communicated to patients during consultations from interview data alone. Future research should capture what actually happens in these interactions by audio recording clinician-patient consultations. Such methods have been used successfully in RCTs to improve the quality of informed consent and recruitment rates(60-62). Furthermore, studies exploring patients' views, particularly those who are offered or have received innovative surgery, are critical if we are to understand the type of information patients draw upon when making decisions about their care. Such work is currently being conducted by our research group as part of a larger research study exploring innovation in surgery(51). It is hoped that this research will aid the development of 'core information sets' (minimum sets of information that should be discussed with patients), which will ultimately support surgeons to discuss innovation and help catalyse the discussion of issues that are important to individual patients(63).

In conclusion, the study suggests that information provision for innovative surgery presents a challenge for surgeons. Our findings also highlight variation in how NHS trusts govern information provision and consent practice for innovative surgery, and further inconsistencies between the views of surgeons and governance representatives. When innovations are introduced under the auspices of research, there are governance structures (e.g. research ethics committees) in place to help ensure patient information is appropriate. However, innovation and modifications to surgery often takes place without formal research approval, which raises the question of how well informed patients are prior to surgery. While the recent RCSEng guidelines for introducing new techniques and technologies represents an important step towards improving informed consent, our study suggests a need to develop support and training programmes to ensure that surgeons consistently deliver appropriate and comprehensive information to patients in the context of innovative surgery.

For peer review only - http://bmjopen.bmj.com/site/about/guidelines.xhtml

Authors' contributions

JMB conceptualised the idea to study informed consent and information provision in the context of surgical innovation. DE developed the idea for the current study with support from JB, JZ, SP, SC, NB, BM, AM and KA. NB, JM, AM and BM identified potential participants. JZ and DE conducted the interviews and analysed the data, with regular discussion of data interpretation with SP and JB. The NIHR Bristol Biomedical Research Centre Surgical Innovation theme is directed by JMB (theme lead) and RH (deputy theme lead). The manuscript was written by JZ, and DE critically reviewed the manuscript and responded to reviewers' comments. All authors commented on the article.

For peer review only

Disclaimer

The views expressed in this publication are those of the authors and not necessarily those of the NHS, the National Institute for Health Research, the MRC or the Department of Health and Social Care.

Data availability

All data relevant to the study are included in the article or uploaded as supplementary information

Competing interests

None declared

References

1. Committee BvFHM. All ER 118,[1957] 1 WLR 582.
2. Surgeons RCo. Consent: Supported Decision-Making-a good practice guide. RCS London; 2016.
3. Coulter A, Hopkins A, Moulton B. Montgomery v Lanarkshire Health Board: transforming informed consent. The Bulletin of the Royal College of Surgeons of England. 2017;99(1):36-8.
4. Faden RR, Beauchamp TL. A history and theory of informed consent: Oxford University Press; 1986.
5. Chan SW, Tulloch E, Cooper ES, Smith A, Wojcik W, Norman JEJB. Montgomery and informed consent: where are we now? 2017;357:j2224.
6. Chan SW, Tulloch E, Cooper ES, Smith A, Wojcik W, Norman JE. Montgomery and informed consent: where are we now? 2017;357:j2224.
7. Royal College of Surgeons. Learning from invited reviews. 2019.
8. Dyer C. Robot assisted surgery is blamed for heart patient's death. 2018;363:k4791.
9. Royal College of Surgeons. Surgical innovation, new techniques and technologies 2019.
10. Bracken-Roche D, Bell E, Karpowicz L, Racine E. Disclosure, Consent, and the Exercise of Patient Autonomy in Surgical Innovation: A Systematic Content Analysis of the Conceptual Literature. Accountability in Research. 2014;21(6):331-52.
11. Di Paolo M, Boggi U, Turillazzi E. Bioethical approach to robot-assisted surgery. 2019.
12. Reitsma A, Moreno J. J Am Coll Surg. 2002;194(null):792.
13. Char SJL, Hills NK, Lo B, Kirkwood KS. Informed consent for innovative surgery: A survey of patients and surgeons. Surgery. 2013;153(4):473-80.
14. Rogers WA, Lotz M, Hutchison K, Pourmoslemi A, Eyers A. Identifying surgical innovation: a qualitative study of surgeons' views. Ann Surg. 2014;259(2):273-8.
15. Apramian T, Watling C, Lingard L, Cristancho S. Adaptation and innovation: a grounded theory study of procedural variation in the academic surgical workplace. J Eval Clin Pract. 2015;21(5):911-8.
16. Danjoux NM, Martin DK, Lehoux PN, Harnish JL, Shaul RZ, Bernstein M, et al. Adoption of an innovation to repair aortic aneurysms at a Canadian hospital: a qualitative case study and evaluation. BMC Health Serv Res. 2007;7(1):182.
17. Sharma B, Danjoux NM, Harnish JL, Urbach DR. How are decisions to introduce new surgical technologies made? Advanced laparoscopic surgery at a Canadian community hospital: A qualitative case study and evaluation. Surg Innov. 2006;13(4):250-6.
18. Johnson J, Hutchison K. They Know How to Work It, That's Their Focus in Life: The Complex Role of Industry Representatives in Surgical Innovation. Journal of Empirical Research on Human Research Ethics. 2018;13(5):461-74.

For peer review only

19. Cousins S, Richards H, Zahra J, Elliott D, Avery K, Robertson HF, et al. Introduction and adoption of innovative invasive procedures and devices in the NHS: an in-depth analysis of written policies and qualitative interviews (the INTRODUCE study protocol). 2019;9(8):e029963.
20. Health Do. Health Service Circular (HSC2003/011) – The Interventional Procedures Programme 2003 [Available from: https://webarchive.nationalarchives.gov.uk/20120503190439/http://www.dh.gov.uk/prod_consum_dh/groups/dh_digitalassets/@dh/@en/documents/digitalasset/dh_4064925.pdf.
21. Willig C. Applied discourse analysis: Social and psychological interventions: Open University Press; 1999.
22. Burman E. Minding the Gap: Positivism, Psychology, and the Politics of Qualitative Methods. 1997;53(4):785-801.
23. Dempster M. A Research Guide for Health and Clinical Psychology: Palgrave Macmillan; 2011.
24. O'Brien BC, Harris IB, Beckman TJ, Reed DA, Cook DA. Standards for reporting qualitative research: a synthesis of recommendations. Academic medicine : journal of the Association of American Medical Colleges. 2014;89(9):1245-51.
25. Patton MQJQsw. Two decades of developments in qualitative inquiry: A personal, experiential perspective. 2002;1(3):261-83.
26. Reitsma AM, Moreno JD. The American Journal of Bioethics. 2003;3(null):49.
27. Johnson J, Rogers W. J Med Ethics. 2011;38(null):9.
28. Hutchison K, Rogers W, Eyers A, Lotz M. Getting Clearer about surgical innovation: a new definition and a new tool to support responsible practice. Ann Surg. 2015;262(6):949-54.
29. Birchley G, Huxtable R, Ives J, Blazeby J. Have We Made Progress in Identifying (Surgical) Innovation? The American Journal of Bioethics. 2019;19(6):25-7.
30. Fassinger REJJocp. Paradigms, praxis, problems, and promise: Grounded theory in counseling psychology research. 2005;52(2):156.
31. Sandelowski MJRin, health. Sample size in qualitative research. 1995;18(2):179-83.
32. Braun V, Clarke V. Successful qualitative research: A practical guide for beginners: sage; 2013.
33. Bowen GA. Naturalistic inquiry and the saturation concept: a research note. 2008;8(1):137-52.
34. Smith JAJRmip. Semi-structured interviewing and qualitative analysis. 1995.
35. Britten NJB. Qualitative research: qualitative interviews in medical research. 1995;311(6999):251-3.
36. Rubin HJ, Rubin IS. Qualitative interviewing: The art of hearing data: sage; 2011.
37. Hammersley P, Atkinson M. Ethnography: principles and practice. London: Routledge; 1983 1983//.
38. Brinkmann S, Kvale S. Interviews: Learning the craft of qualitative research interviewing: Sage Thousand Oaks, CA; 2015.
39. Fielding N, Thomas H. Qualitative interviewing. 2008.
40. Charmaz K, Belgrave LJTShoirTcotc. Qualitative interviewing and grounded theory analysis. 2012;2:347-65.
41. Glaser BG, Strauss AL. The Discovery of Grounded Theory. Aldine Transaction: USA; 1967.
42. Seale C, Silverman DJTEJoPH. Ensuring rigour in qualitative research. 1997;7(4):379-84.
43. Krippendorff KJHcr. Reliability in content analysis: Some common misconceptions and recommendations. 2004;30(3):411-33.
44. Miles MB, Huberman AM, Huberman MA, Huberman M. Qualitative data analysis: An expanded sourcebook: sage; 1994.
45. Pope C, Ziebland S, Mays N. Analysing qualitative data. BMJ. 2000;320(7227):114-6.
46. Braun V, Clarke V. Using thematic analysis in psychology. Qualitative Research in Psychology. 2006;3(2):77-101.

For peer review only - http://bmjopen.bmj.com/site/about/guidelines.xhtml

47. Charmaz K. Constructing grounded theory: A practical guide through qualitative analysis: sage; 2006.
48. Barbour RSJB. Checklists for improving rigour in qualitative research: a case of the tail wagging the dog? 2001;322(7294):1115-7.
49. Dey I. Qualitative data analysis: A user friendly guide for social scientists: Routledge; 2003.
50. Rogers WA, Lotz M, Hutchison K, Pourmoslemi A, Eyers A. Identifying surgical innovation: a qualitative study of surgeons' views. Ann Surg. 2014;259(2):273-8.
51. McNair AGK, Main BJ, Elliott D. Comment on: Bioethical approach to robot-assisted surgery. 2020;107(1):150-.
52. Mitchell S, Gass J, Hanna M. How Well Informed Do Patients Feel about Their Breast Cancer Surgery Options? Findings from a Nationwide Survey of Women after Lumpectomy and/or Mastectomy. Journal of the American College of Surgeons. 2018;226(2):134-46.e3.
53. Kinnersley P, Phillips K, Savage K, Kelly MJ, Farrell E, Morgan B, et al. Interventions to promote informed consent for patients undergoing surgical and other invasive healthcare procedures. 2013(7).
54. Ford KE, Cooper LRL. Learning from lawsuits: Ten-years of NHS litigation authority claims against 11 surgical specialities in England. The Surgeon. 2018;16(1):27-35.
55. Veerman MM, van der Woude LA, Tellier MA, Legemaate J, Scheltinga MR, Stassen LPS, et al. A decade of litigation regarding surgical informed consent in the Netherlands. Patient Education and Counseling. 2019;102(2):340-5.
56. Hallock JL, Rios R, Handa VL. Patient satisfaction and informed consent for surgery. American journal of obstetrics and gynecology. 2017;217(2):181.e1-.e7.
57. Lee Char SJ, Hills NK, Lo B, Kirkwood KS. Informed consent for innovative surgery: a survey of patients and surgeons. Surgery. 2013;153(4):473-80.
58. Townsend D, Mills N, Savović J, Donovan JL. A systematic review of training programmes for recruiters to randomised controlled trials. Trials. 2015;16:432.
59. Elliott D, Husbands S, Hamdy FC, Holmberg L, Donovan JL. Understanding and Improving Recruitment to Randomised Controlled Trials: Qualitative Research Approaches. Eur Urol. 2017;72(5):789-98.
60. Elliott D, Husbands S, Hamdy FC, Holmberg L, Donovan JL. Understanding and Improving Recruitment to Randomised Controlled Trials: Qualitative Research Approaches. European urology. 2017;72(5):789-98.
61. Rooshenas L, Paramasivan S, Jepson M, Donovan JLJQhr. Intensive Triangulation of Qualitative Research and Quantitative Data to Improve Recruitment to Randomized Trials: The Quintet Approach. 2019;29(5):672-9.
62. Donovan JL, Rooshenas L, Jepson M, Elliott D, Wade J, Avery K, et al. Optimising recruitment and informed consent in randomised controlled trials: the development and implementation of the Quintet Recruitment Intervention (QRI). Trials. 2016;17(1):283.
63. Main BG, McNair AGK, Huxtable R, Donovan JL, Thomas SJ, Kinnersley P, et al. Core information sets for informed consent to surgical interventions: baseline information of importance to patients and clinicians. BMC medical ethics. 2017;18(1):29.

For peer review only - http://bmjopen.bmj.com/site/about/guidelines.xhtml

Reporting checklist for qualitative study.

Based on the SRQR guidelines.

Instructions to authors

Complete this checklist by entering the page numbers from your manuscript where readers will find each of the items listed below.

Your article may not currently address all the items on the checklist. Please modify your text to include the missing information. If you are certain that an item does not apply, please write "n/a" and provide a short explanation.

Upload your completed checklist as an extra file when you submit to a journal.

In your methods section, say that you used the SRQRreporting guidelines, and cite them as:

O'Brien BC, Harris IB, Beckman TJ, Reed DA, Cook DA. Standards for reporting qualitative research: a synthesis of recommendations. Acad Med. 2014;89(9):1245-1251.

		Reporting Item	Page Number
**Title**			
	#1	Concise description of the nature and topic of the study identifying the study as qualitative or indicating the approach (e.g. ethnography, grounded theory) or data collection methods (e.g. interview, focus group) is recommended	1
**Abstract**			
	#2	Summary of the key elements of the study using the abstract format of the intended publication; typically includes background, purpose, methods, results and conclusions	2
**Introduction**			
Problem formulation	#3	Description and signifcance of the problem / phenomenon studied: review of relevant theory and empirical work; problem statement	4

For peer review only

Purpose or research question	#4	Purpose of the study and specific objectives or questions	5
**Methods**			
Qualitative approach and research paradigm	#5	Qualitative approach (e.g. ethnography, grounded theory, case study, phenomenolgy, narrative research) and guiding theory if appropriate; identifying the research paradigm (e.g. postpositivist, constructivist / interpretivist) is also recommended; rationale. The rationale should briefly discuss the justification for choosing that theory, approach, method or technique rather than other options available; the assumptions and limitations implicit in those choices and how those choices influence study conclusions and transferability. As appropriate the rationale for several items might be discussed together.	7
Researcher characteristics and reflexivity	#6	Researchers' characteristics that may influence the research, including personal attributes, qualifications / experience, relationship with participants, assumptions and / or presuppositions; potential or actual interaction between researchers' characteristics and the research questions, approach, methods, results and / or transferability	6; 8
Context	#7	Setting / site and salient contextual factors; rationale	6
Sampling strategy	#8	How and why research participants, documents, or events were selected; criteria for deciding when no further sampling was necessary (e.g. sampling saturation); rationale	5-6
Ethical issues pertaining to human subjects	#9	Documentation of approval by an appropriate ethics review board and participant consent, or explanation for lack thereof; other confidentiality and data security issues	5
Data collection methods	#10	Types of data collected; details of data collection procedures including (as appropriate) start and stop	5-7

For peer review only

		dates of data collection and analysis, iterative process, triangulation of sources / methods, and modification of procedures in response to evolving study findings; rationale	
Data collection instruments and technologies	#11	Description of instruments (e.g. interview guides, questionnaires) and devices (e.g. audio recorders) used for data collection; if / how the instruments(s) changed over the course of the study	6-7
Units of study	#12	Number and relevant characteristics of participants, documents, or events included in the study; level of participation (could be reported in results)	8
Data processing	#13	Methods for processing data prior to and during analysis, including transcription, data entry, data management and security, verification of data integrity, data coding, and anonymisation / deidentification of excerpts	7-8
Data analysis	#14	Process by which inferences, themes, etc. were identified and developed, including the researchers involved in data analysis; usually references a specific paradigm or approach; rationale	7-8
Techniques to enhance trustworthiness	#15	Techniques to enhance trustworthiness and credibility of data analysis (e.g. member checking, audit trail, triangulation); rationale	7-8
**Results/findings**			
Syntheses and interpretation	#16	Main findings (e.g. interpretations, inferences, and themes); might include development of a theory or model, or integration with prior research or theory	8-14
Links to empirical data	#17	Evidence (e.g. quotes, field notes, text excerpts, photographs) to substantiate analytic findings	8-14
**Discussion**			
Intergration with prior work, implications,	#18	Short summary of main findings; explanation of how findings and conclusions connect to, support, elaborate on, or challenge conclusions of earlier	15-17

For peer review only

transferability and contribution(s) to the field		scholarship; discussion of scope of application / generalizability; identification of unique contributions(s) to scholarship in a discipline or field	
Limitations	#19	Trustworthiness and limitations of findings	16
**Other**			
Conflicts of interest	#20	Potential sources of influence of perceived influence on study conduct and conclusions; how these were managed	N/A
Funding	#21	Sources of funding and other support; role of funders in data collection, interpretation and reporting	17

The SRQR checklist is distributed with permission of Wolters Kluwer © 2014 by the Association of American Medical Colleges. This checklist was completed on 12. February 2020 using https://www.goodreports.org/, a tool made by the EQUATOR Network in collaboration with Penelope.ai

For peer review only

Topic guide for interviews with data committee members

Introduction:

- Thank for taking time
- Not medical professional, want to understand process of implementing new procedures and devices
- No right or wrong answers, can stop the interview at any time
- Take verbal consent

Can you tell me about yourself?

- What is your current position and role?
- Particular areas of interest/expertise?
- How did you become involved in [committee]? When? How found it? What you expected?
- Can you tell me about the committee? What is the role of the committee?
    - Who involved? How often meet?

As you know, we are looking at innovation in surgery. Can you talk me through how you would define innovation? *(Newness; degree of change, level of risk, impact?)*

Can you talk me through how applications are processed, from submissions to decision-making?

- What is the process for introducing surgical innovation in [site]?
- Do you think there are similarities/difference between innovation and research? In what ways?
- How many applications do you receive each year?
- What information is provided? *(What is compulsory?)*
- How are they considered?
    - Potential benefit? Safety (and how quantified?) Evidence? Cost? Patient acceptability?
- Why would an application be declined? Example? Agreement between committee members?
- What happens to those procedures that are declined? Is there an option to appeal?

What's your understanding of how these procedures are integrated into practice?

- What happens once a technique/device is approved by the committee?
- If approved, is there any monitoring? How? When?
- What if there is a problem with a novel procedure?
- Is there any guidance for communicating about novel procedures?
- What do you think patients are told about the novel component? What should they be told - What aspects important to communicate?
- What is included on patient info sheets provided

Your views on the committee

- How do you feel about being involved in the decision-making process?
- Are there any particularly challenging aspects?
- What are the strengths of the committee?
- Do you think it could be improved in anyway?

Ending the interview

- Is there anything else that I haven't mentioned?
- Anyone else you recommend I could contact?

For peer review only

- Before we finish (if not covered) – role (registrar/consultant), years in your profession, highest qualification

For peer review only

Topic guide for interviews stakeholders

Introduction:

- Thank you for taking time
- Not medical professional, want to understand perspectives of innovation
- No right or wrong answers, can stop the interview at any time
- Take verbal consent

Can you tell me about yourself?

- What is your current position and role?
- Can you describe the range /type(s) of surgical procedures you undertake?
- Particular areas of interest/expertise?

As you know, we are looking at innovation in surgery. Can you talk me through how you would define innovation? *(Newness; degree of change, level of risk, impact?)*

Have you conceptualised a new procedure/device/modification of a procedure?

- How did it come about? When?
- Would you regard this as an innovation or a modification?
- Why do you feel it's needed?
- Who is suitable for the procedure? *(How determined?)*
- What treatment options are currently available to them? What happens if patients don't get the procedure? (Are they disadvantaged in any way?)
- What do you hope will be the outcomes from the new procedure? How estimated?

Do you think patients should be informed about new procedures/devices?

- What does 'informed consent' mean to you?
- Is there any guidance for communicating novel procedure?
- What should they be told? Extent that procedure is novel? Uncertainty? Risks? Harms?
- Is informing patients about a new procedure challenging in anyway?
- How do you think patients will respond to the procedure?
- How much of a modification would you need to do to tell a patient something was new?
- When do you stop telling them it is new?

Looking ahead

- Do you think any subgroups of patients would be more suited to the procedure? Are there any that you would feel less comfortable with having the procedure?
- Do you have any concerns about the procedure? Risks to patients? Costs?
- Do you have any plans to disseminate the results? How? All results (good and bad)? Where? When? Who's responsible for this?

Ending the interview

- Is there anything else that I haven't mentioned?
- Anyone else you recommend I could contact?
- Thank for time, summarise key points.

For peer review only

BMJ Open

Discussing surgical innovation with patients: A qualitative study of surgeons' and governance representatives' views

Journal:	*BMJ Open*
Manuscript ID	bmjopen-2019-035251.R2
Article Type:	Original research
Date Submitted by the Author:	20-May-2020

Complete List of Authors:	Zahra, Jesmond; University of Bristol, National Institute for Health Research Bristol Biomedical Research Centre Surgical Innovation Theme, Bristol Centre for Surgical Research, Bristol Medical School: Population Health Sciences, University of Bristol<br>Paramasivan, Sangeetha ; University of Bristol, National Institute for Health Research Bristol Biomedical Research Centre Surgical Innovation Theme, Bristol Centre for Surgical Research, Bristol Medical School: Population Health Sciences, University of Bristol<br>Blencowe, Natalie; University of Bristol, National Institute for Health Research Bristol Biomedical Research Centre Surgical Innovation Theme, Bristol Centre for Surgical Research, Bristol Medical School: Population Health Sciences, University of Bristol; University Hospitals Bristol NHS Foundation Trust, Division of Surgery<br>Cousins, Sian; University of Bristol, National Institute for Health Research Bristol Biomedical Research Centre Surgical Innovation Theme, Bristol Centre for Surgical Research, Bristol Medical School: Population Health Sciences, University of Bristol<br>Avery, Kerry; University of Bristol, National Institute for Health Research Bristol Biomedical Research Centre Surgical Innovation Theme, Bristol Centre for Surgical Research, Bristol Medical School: Population Health Sciences, University of Bristol<br>Mathews, Johnny; University of Bristol, National Institute for Health Research Bristol Biomedical Research Centre Surgical Innovation Theme, Bristol Centre for Surgical Research, Bristol Medical School: Population Health Sciences, University of Bristol<br>Main, Barry; University of Bristol, National Institute for Health Research Bristol Biomedical Research Centre Surgical Innovation Theme, Bristol Centre for Surgical Research, Bristol Medical School: Population Health Sciences, University of Bristol; University Hospitals Bristol NHS Foundation Trust<br>McNair, Angus; University of Bristol, National Institute for Health Research Bristol Biomedical Research Centre Surgical Innovation Theme, Bristol Centre for Surgical Research, Bristol Medical School: Population Health Sciences, University of Bristol; North Bristol NHS Trust, GI Surgery<br>Hinchliffe, Robert; University of Bristol, National Institute for Health Research Bristol Biomedical Research Centre Surgical Innovation Theme, Bristol Centre for Surgical Research, Bristol Medical School: Population Health Sciences, University of Bristol; North Bristol NHS Trust, Vascular Services<br>Blazeby, Jane; University of Bristol, National Institute for Health

Research Bristol Biomedical Research Centre Surgical Innovation Theme, Bristol Centre for Surgical Research, Bristol Medical School: Population Health Sciences, University of Bristol; University Hospitals Bristol NHS Foundation Trust
Elliott, Daisy; University of Bristol, National Institute for Health Research Bristol Biomedical Research Centre Surgical Innovation Theme, Bristol Centre for Surgical Research, Bristol Medical School: Population Health Sciences, University of Bristol

**Primary Subject Heading**:	Surgery
Secondary Subject Heading:	Communication
Keywords:	SURGERY, Health policy < HEALTH SERVICES ADMINISTRATION & MANAGEMENT, QUALITATIVE RESEARCH

SCHOLARONE™ Manuscripts

I, the Submitting Author has the right to grant and does grant on behalf of all authors of the Work (as defined in the below author licence), an exclusive licence and/or a non-exclusive licence for contributions from authors who are: i) UK Crown employees; ii) where BMJ has agreed a CC-BY licence shall apply, and/or iii) in accordance with the terms applicable for US Federal Government officers or employees acting as part of their official duties; on a worldwide, perpetual, irrevocable, royalty-free basis to BMJ Publishing Group Ltd ("BMJ") its licensees and where the relevant Journal is co-owned by BMJ to the co-owners of the Journal, to publish the Work in this journal and any other BMJ products and to exploit all rights, as set out in our licence.

The Submitting Author accepts and understands that any supply made under these terms is made by BMJ to the Submitting Author unless you are acting as an employee on behalf of your employer or a postgraduate student of an affiliated institution which is paying any applicable article publishing charge ("APC") for Open Access articles. Where the Submitting Author wishes to make the Work available on an Open Access basis (and intends to pay the relevant APC), the terms of reuse of such Open Access shall be governed by a Creative Commons licence – details of these licences and which Creative Commons licence will apply to this Work are set out in our licence referred to above.

Other than as permitted in any relevant BMJ Author's Self Archiving Policies, I confirm this Work has not been accepted for publication elsewhere, is not being considered for publication elsewhere and does not duplicate material already published. I confirm all authors consent to publication of this Work and authorise the granting of this licence.

For peer review only

Discussing surgical innovation with patients: A qualitative study of surgeons' and governance representatives' views

Submission category: Original article

J Zahra[1], S Paramasivan[1], N.S Blencowe[1,3], S Cousins[1], K Avery[1], J Mathews[1,2], B Main [1,3], A McNair [1,2], R Hinchliffe[1,2], J.M Blazeby[1,3], D Elliott[1]

[1]National Institute for Health Research Bristol Biomedical Research Centre Surgical Innovation Theme, Centre for Surgical Research, Population Health Sciences, Bristol Medical School, University of Bristol, 39 Whatley Road, Clifton, Bristol, UK. BS8 2PS;

[2]North Bristol NHS Trust, Bristol, UK. BS10 5NB.

[3]University Hospitals Bristol NHS Foundation Trust, Bristol, UK. BS2 8HW

Running title: Discussing surgical innovation with patients

Keywords: consent, surgery, information provision, innovation, qualitative,

Corresponding author details: Daisy Elliott [daisy.elliott@bristol.ac.uk]

Funding statement: This study was supported by the NIHR Biomedical Research Centre at University Hospitals Bristol NHS Foundation Trust and the University of Bristol, the MRC ConDuCT-II (Collaboration and innovation for Difficult and Complex randomised controlled Trials In Invasive procedures) Hub for Trials Methodology Research (MR/K025643/1). The views expressed in this publication are those of the author(s) and not necessarily those of the NHS, the National Institute for Health Research or the Department of Health and Social Care. BM is a NIHR Clinical Lecturer, AM is funded by a Clinician Scientist Fellowship (NIHR-CS-2017-17-010) from the National Institute for Health Research. NB is funded by an MRC Clinician Scientist Award.

Word count: 4380

For peer review only - http://bmjopen.bmj.com/site/about/guidelines.xhtml

Abstract

Objectives

Little is known about how innovative surgical procedures are introduced and discussed with patients. This qualitative study aimed to explore perspectives on information provision and consent prior to innovative surgical procedures.

Design

Qualitative study involving semi-structured interviews. Interviews were audio recorded, transcribed and analysed thematically.

Participants

42 interviews were conducted (26 surgeons and 16 governance representatives).

Setting

Surgeons and governance representatives recruited from various surgical specialities and National Health Service Trusts across England, United Kingdom.

Results

Participants stated that if a procedure was innovative, patients should be provided with additional information extending beyond that given during routine surgical consultations. However, difficulty defining innovation had implications for whether patients were informed about novel components of surgery and how the procedure was introduced (i.e. as part of a research study, trust approval or in routine clinical practice). Furthermore, data suggests surgeons found it difficult to establish what information is essential and how much detail is sufficient, and governance surrounding written and verbal information provision differed between NHS trusts. Generally, surgeons believed patients held a view that 'new' was best and reported that managing these expectations could be difficult, particularly if patient views aligned with their own.

Conclusions

This study highlights the challenges of information provision and obtaining informed consent in the context of innovative surgery, including establishing if and how a procedure is truly innovative, determining the key information to discuss with patients, ensuring information provision is objective and balanced, and managing patient expectations and preferences. This suggests that surgeons may require support and training to discussing novel procedures with patients. Further work should

For peer review only - http://bmjopen.bmj.com/site/about/guidelines.xhtml

capture consultations where new procedures are discussed with patients and patients' views of these information exchanges.

For peer review only

For peer review only - http://bmjopen.bmj.com/site/about/guidelines.xhtml

Strengths and limitations

- This is the first qualitative study to conduct in-depth, semi structured interviews to understand current practice for information provision and informed consent for innovative surgical procedures in the United Kingdom.
- Interviews with governance representatives enabled us to understand what policies were in place for the introduction of new procedures, whilst surgeons provided insights into how they introduced new procedures and discussed these with patients.
- Purposeful sampling ensured that participants were recruited from a range of geographical locations and trust types, and surgeons were from different specialities with varying experiences of innovation.
- Audio-recording appointments where clinicians discuss innovative surgeries and patients decide whether or not to undergo the procedure would strengthen our understanding of interactions between clinicians and patients.
- Further work should capture patients' views on information provision in this context, so that recommendations can be made to improve transparency and communication.

For peer review only

Introduction

Informed consent is fundamental to patient autonomy and represents a cornerstone of modern healthcare provision. Until recently, the Bolam ruling meant that a doctor's duty to inform patients prior to surgery was judged based on whether they had acted in line with the view of a responsible body of medical opinion [1 2]. The 2015 Montgomery ruling[3] redefined the standard of informed consent in the United Kingdom (UK) and represented a shift from a paternalistic model of consent to a more patient-centred approach[4 5] – meaning that a patient should be told whatever they want to know, not what the doctor thinks they should be told[6].

Recently, important questions have been raised surrounding informed consent for innovative surgical procedures[7 8]. An inquest into the death of the first patient in the UK to undergo robotically assisted heart surgery found the patient had not been fully informed about the comparative risk of robotic versus conventional open surgery[8]. In addition, a summary of invited reviews published by the Royal College of Surgeons of England (RCSEng) raised further concerns[7]. The review highlighted issues surrounding surgeons' ability to accurately quantify risk as reported in published literature, whether surgical experience is being sufficiently disclosed and effectively communicated to patients, and the overall quality of patient-clinician discussions[7]. The RESEng subsequently published guidelines for the development, implementation and dissemination of surgical innovation, which contains guidance on informed consent [9]. This suggests, based on findings of a review by Broekman et al [9], that patients should be informed of the innovative nature of the procedure, surgeons' experience with the procedure and the learning curve, the risk and benefits – including unknown or unforeseeable risks or outcomes, the evidence or lack thereof, and alternatives to the innovative procedure.

Reviews and commentaries have highlighted potential issues around informed consent for innovative surgical procedures, including patients' and surgeons' beliefs that new treatments constitute improved treatment options or result in better outcomes [10 11]. However, little is known about current practice. Surveys that have been conducted in the United States suggest that information varies considerably in content and quality. Reitsma and Moreno (2002) reported that of twenty-one surgeons, 75% verbally informed patients about the innovative nature of the procedure but only 33% provided written information [12]. Char and colleagues (2013), in a scenario based survey carried out with 85 surgeons and 383 patients, found differences between what surgeons thought patients wanted to know about innovative procedures and what patients reported they wanted to know. For instance, compared with surgeons, patients placed more importance on nearly all types of information, particularly volumes and outcomes [13].

For peer review only - http://bmjopen.bmj.com/site/about/guidelines.xhtml

Whilst a handful of qualitative studies have been conducted exploring how innovative procedures have been introduced [14 15 16 17], none have specifically looked at information provision and informed consent. Moreover, these studies were conducted in Australia and Canada and findings may not be generalisable to other countries and different healthcare systems[18]. New invasive procedures may be introduced in the context of formal research studies or via local hospital policies [19]. The National Institute for Health and Care Excellence (NICE) recommends that local NHS organisations have appropriate governance structures in place to review, approve and monitor the introduction of new invasive procedures [20]. The current study sought to explore surgeons' and governance representatives' views of information provision and informed consent for the introduction of new invasive procedures in the United Kingdom (UK).

Methods

Patient and public involvement

Public and patient involvement was not conducted as part of the current study. However, the current findings have been discussed with a recently established group, which has led to further work which specifically aims to explore patient views on surgical innovation.

Design

This qualitative study consisted of semi-structured interviews and used a grounded theory methodology. Governance representatives (defined as those involved in regulating the introduction of new/modified procedures and/or devices at trust or national levels) were included to understand what processes were in place for the introduction of new procedures [17]. Surgeons were recruited to explore experiences of introducing new/modified procedures or devices into clinical practice. As there is no standardised definition for innovation and it is often a continuum from standard practice through to true innovation [14 21 22], and because surgical innovation is unlikely to involve a single discrete development [23 24], participants were asked to reflect what the term meant to them and describe procedures that they deemed to be innovative. Ethical approval was granted by the University of Bristol Faculty of Health Sciences Research Ethics Committee. The study is reported in line with the Standards for Reporting Qualitative Research (SRQR; Additional File 1) [25].

Sampling and recruitment

Participants were recruited between November 2017 and October 2018 using a combination of purposive [26]and snowball sampling techniques [27]. Governance representatives were identified

For peer review only - http://bmjopen.bmj.com/site/about/guidelines.xhtml

through websites (e.g. National Health Service (NHS) Trusts websites) and NHS policy documents (e.g. new invasive procedure/devices policies). An initial sample of surgeons were identified by academic surgeons working within the Centre for Surgical Research, University of Bristol, UK. Subsequent surgeons were identified via snowball sampling[27], in which interviewees recommended others who may be willing to take part. Sampling was regularly reviewed to ensure surgeons from different specialities and geographical locations, with varying experiences of innovation (e.g. minor modifications vs first in human procedures), were recruited to capture a diversity of perspectives [28 29]. For governance representatives, maximum variation was sought in relation to different roles (e.g. new procedures committee members, medical directors), geographical locations, trust types, and different surgical specialities.

Potential participants were approached via email and up to three invitation emails were sent before contact attempts ceased. In total, 83 potential participants were invited to take part (38 surgeons and 45 governance representatives). Study recruitment ceased when the qualitative team agreed that theoretical saturation had been achieved (where no new themes were forthcoming from three consecutive interviews)[30 31].

Data collection

All participants received a study information leaflet and provided written consent before taking part. Separate topic guides were developed for governance representatives and surgeons. All interview schedules contained core topics of interest (e.g. 'How would you define innovation?', 'What does informed consent mean to you?')[32 33], although these were flexible to allow participants to shape the direction of conversations[34 35]. Governance representatives were asked how applications for new invasive procedures were processed and how these procedures then integrated into practice, whereas surgeons were asked about their experiences of introducing procedures and what patients had been told. An example of each interview schedule is provided in the supplementary files (Additional files 2 and 3).

Interviews were considered to be the most appropriate method for this study because they provided the opportunity to encourage participants to think carefully about their own experiences [36], enable the interviewer to respond and follow up on issues raised by the interviewee [37], and that some participants may feel intimidated at the prospect of discussing their experiences within a focus group setting[27]. The interviewer began the discussion with more general questions to build rapport [38] such as the participants' background and general views of defining innovation. The interviewee's comments were restated and incorporated into further questions to check with the interviewer that they had understood correctly[34]. In order to gain more detailed and comprehensive understanding

For peer review only - http://bmjopen.bmj.com/site/about/guidelines.xhtml

of the descriptions given by participants, they were asked to elaborate their comments with explanations and examples (such as, 'Why do you feel this was?')[34], or fillers (such as 'mmm' or 'yeah') were used by the interviewer to show the participant this was something they were interested in and to encourage them to continue [39]. Towards the end of the interview, participants were given an opportunity to raise issues that were important to them that had not already been covered [38].

Topic guides were developed iteratively during the study period, in consideration of emerging insights from interviews and better ways of phrasing questions[32 40]. For instance, interviewers (non-clinical academics) used more prompts to encourage the surgeons to describe procedures in a non-technical manner [34]. Topic guides were initially very long so were reduced to include more 'mapping' questions that addressed the core components of interest (such as, 'I understand you've conceptualised a new procedure. Can you talk me through this?'). Additional prompts were added from emerging findings (i.e. colleagues' reactions to procedures and written information). With permission, interviews were audio recorded using an encrypted digital dictaphone.

All interviews were audio recorded expect one where the interviewee requested not to be recorded and therefore written notes were taken. All interviews were conducted by one of two trained and experienced qualitative researchers (JZ or DE). JZ is a male Senior Research Associate with a background in public health research. DE is a female Research Fellow with experience working on surgical trials. She is also a member of the QuinteT research group, which uses qualitative research methods to optimise recruitment and informed consent to randomised controlled trials (RCTs). JZ and DE have several years of experience conducting qualitative research, and each has a degree in Psychology and a PhD in health-related research.

Data analysis

Interview recordings were transcribed and de-identified so that names and identifiable information were removed. Thematic analysis was undertaken using the constant comparison technique of grounded theory [41 42], which involved using the inductive identification of codes from the data to generate new hypotheses about phenomena that are derived or grounded in the data [43]. Its central principle is of constant comparison, where new findings are systematically compared with existing data so that similarities and differences can be identified and emerging theories refined through the ongoing assimilation of data [41 44].

For peer review only - http://bmjopen.bmj.com/site/about/guidelines.xhtml

Data were imported into a data management program (NVivo version 11) to organise the data systematically, allow for quick searching and refining of codes and categories, increase the transparency of the analysis process and enable team comparisons of coding [30 45]. First, analysts (JZ, DE & SP) read and re-read transcripts before independently coding a sample of five transcripts to begin familiarising themselves with the data [30 46]. Interesting features were coded, whereby a segment of data was assigned a code [40], and on-going potential ideas were noted at every stage of analysis[47]. Data coding was compared to ensure consistency and rigour in the findings[48], whereby differences were discussed thoroughly until a consensus was met and an initial coding frame was developed. The coding frame was then independently applied to three transcripts by JZ, DE and SP. High consistency between analysts indicated the coding frame appropriately captured the data. JZ then applied the coding frame to the remaining transcripts, adding additional codes if important data were missed by the original coding frame. To form preliminary themes and subthemes, codes were collated into hierarchical groups[49]. Emerging findings were regularly discussed with JMB and NB (both academic surgeons), with reference to the raw transcripts, to ensure they fully encapsulated the meaning of the data[48]. Finally, a descriptive account of the themes and subthemes, which included representative quotes, was developed.

Results

Participants

Of the 83 participants that were approached, 42 participants were recruited (those who did not participate were unable to find time for an interview or did not respond to study invites). The final sample included 26 surgeons and 16 governance representatives from across 23 NHS Trusts. Trusts varied in geographical location, type/size (i.e. teaching, specialist, large, multi-centre) and foundation status. Governance representatives' roles within trusts and NICE varied (e.g. medical director, director for quality improvement, head of governance), however, all played a role in regulating the introduction of new surgical procedures and/or devices. At the time of interview, all but one (retired) surgeon worked as a consultant within the NHS. Surgical specialities included cardiothoracic, gastrointestinal, breast, ophthalmology, neurosurgery, urology and orthopaedics. Participants were mostly male (87%). Interviews were conducted over the phone (55%) or face-to-face at participants' place of work or at a university. Interviews lasted an average of 43 minutes (range 22 – 112 minutes, standard deviation 17 minutes).

Analysis

For peer review only - http://bmjopen.bmj.com/site/about/guidelines.xhtml

Findings related to informed consent are presented under four main themes: *difficulty defining innovation*, d*iffering views on what – and how - patients should be told, the challenges of discussing innovation,* and *managing patient expectations*. All quotations are followed by participants' unique identification number, gender, surgical speciality and trust identification number.

Difficulty defining innovation

Surgeons and governance representatives found it challenging to define surgical innovation and considered the term 'innovation' to encompass a spectrum of changes to surgical healthcare. Examples ranged from changing patient pathways through to first in human procedures. Consequently many felt innovation should be viewed as a continuum, rather than a binary concept:

> *"Well there's a spectrum isn't there, I mean it's difficult to define. So something that is innovative to me is something that is new, it's a new way of looking at it, it's a slightly different paradigm, a different way of thinking. And an adaptation is just a sort of a natural evolution of something that's already pre-existing, I suppose that's how I would define them, but clearly it's a spectrum isn't it?" (Surgeon 18, General surgery, Male, Trust 3)*

There was variation in how the procedure had been introduced in participants' hospitals. Within this, surgeons' own experiences included requesting approval from their trust's new procedure committee, conducting the procedure in the context of a research study or deeming that approval was not necessary as the procedure was a variation of an existing one (from either judgement of the surgeon or confirmation from a committee). Table 1 highlights that the challenges of defining innovation, alongside the required technical knowledge of the procedure, could cause uncertainty as to what governance was required.

Table 1: Participants' experiences of governance for new procedures

	**Example quote**
**Procedure considered and approved by trust's new procedure committee**	*"There's one surgeon in [European country] who described the procedure, and the surgeon in [Asian country] who had done some of these procedures, but not really clear on how they'd done it. I contacted the [European] surgeon. He gave me some pointers, so I discussed it with my colleagues, and of course put it through the governance processes as a novel and a new procedure. And then we did it." (Surgeon 31, Colorectal, Male, Trust 8)*
**Approval from trust's new procedure committee deemed unnecessary as felt to be a variation of established procedure**	*"We asked about it to the new procedures committee, but they said well it's basically [variation of established procedure] isn't it. The technicalities of it are lost on non-thoracic surgeons really, which is good." (Surgeon 31, General surgery, Male, Trust 8)*

For peer review only - http://bmjopen.bmj.com/site/about/guidelines.xhtml

For peer review only

	"There's a new technique that's probably done by no more than 9 or 10 surgeons in the country. Again, it's a new technique, it's only been reported in the last 3 or 4 years. […] Because it was under the umbrella of [broader procedure], I didn't have to go through any, so within the hospital there's a board about new techniques, you have to sort of, clinical effectiveness group, you have to have that approved. But, because this was a variation, we didn't need that approval." (Surgeon 22, Male, Orthopedics, Trust 7)
**Procedure evaluated in the context of a research study**	*"We were looking [procedure], and so that was in the guise of a randomised double-blind trial that we put through Ethics. So that would be innovative. We've got an Ethics Committee, and as part of that if you were going to be doing something really brand new that would have to be conducted as part of a trial, so, but when there's a blur between doing something slightly different then no I don't think it's the place for that […] I can't think of anything where they've been involved in, you know, anything that we're doing." (Surgeon 46, Male, Orthopedics, Trust 22)*

Although participants initially stated that patients should be informed about new procedures, some drew upon experiences that contradicted this as they reflected upon the complexity of defining innovation:

"I think absolutely they should be informed, I think that's a tricky one actually… when would I inform a patient about a new instrument I was using? So we don't- if- if you're not trialling the instrument to see if it's safe or more effective or less effective, then I don't think they need to be informed." (Surgeon 4, Male, General and upper gastrointestinal, Trust 12)

"We kind of created our own version of how you do that operation […] So, it's not particularly been described before by surgeons and it's not the world's most different, but it's got a few little innovative techniques. So, that was an operation we designed that was new […] I don't even explain how that's different to [standard procedure] because I think it's far too technically nuanced for a patient to understand… We view it as just a variation of [standard procedure]." (Surgeon 22, Thoracic, Male, Trust 7)

Several governance representatives also described instances where they had become aware of patients not being fully informed about new procedures and emphasised the need for improved information provision:

"So we then audit the first case to make sure that the clinician has been up front with the individual about the fact that this is the first time that this particular procedure has been undertaken…if they haven't, there's been a couple of

For peer review only - http://bmjopen.bmj.com/site/about/guidelines.xhtml

occasions when they haven't, we tell them off and say do better next time and we audit the second case or whatever, so we're confident they've got it right." (Governance representative 29, Director of research, Male, Trust 5)

"We have had a couple of near misses … one was a surgeon inviting another surgeon from another hospital to come and show him how to do a procedure, that person did not have an honorary contract with the Trust therefore shouldn't have been operating and nobody knew that that was occurring. And we have had a rep [industry representative] coming into theatres to bring some samples of a new piece of equipment and most certainly patients weren't consented that it was going to be used on them … So I think there is increasing awareness now, certainly when business cases are presented, they do need to follow this process if that's what's going to happen. However there is also, can I put my hand on my heart and say actually the surgeon's not going to invite a rep into theatre tomorrow? I probably couldn't, but I do know that the theatre staff feel more empowered to be able challenge that behaviour, so actually if I knew that somebody was going to use a piece of equipment they've never used before I would probably be asking the question as to why." (Surgeon 9, Upper gastrointestinal, Male, Trust 3)

Differing views on what – and how - patients should be told

There was consensus from all participants that if a procedure was deemed innovative, patients should be provided with additional information extending beyond that given during routine surgical consultations. Within this, the potential risks and benefits, alternative treatment options, and the novel status of the procedure were universally regarded as essential information that should be discussed.

"We tell them that it's a new procedure and previously we've told them how many we've done prior to this" (Surgeon 5, Thoracic, Male, Trust 5)

"You have to give them options, you have to let them know what the evidence base is." (Surgeon 46, Trauma & Orthopaedic, Male, Trust 22)

There was variation in views as to whether other information - such as the evidence base (or lack thereof), operative experience, training, safety precautions implemented in theatre and national guidance - should be disclosed. Taken together, there appeared to be uncertainty at precisely how much to tell patients:

"Sometimes I tell them too much or tell them... I mean, the latest, er, ruling of Montgomery as I understand it is that they need to be told what they would want to know. Bloody hell. But then you've got to judge what they want to know and you've

For peer review only - http://bmjopen.bmj.com/site/about/guidelines.xhtml

got to have some kind of communication with them and reasonably understand what it is they want to know." (Surgeon 31, Colorectal, Male, Trust 8)

"There is a difficult balance I think, and I'm not sure we've got it [consent] right yet actually." (P7)

Many interviewees felt strongly that patients should be provided with verbal (i.e. discussions during consultation) *and* written information (i.e. via a patient information leaflet (PIL)) when being offered innovative surgery. In line with this, most governance representatives reported that innovative procedures could only be introduced if an appropriate PIL had been developed (although there was variation as to whether this was reviewed by the committee).

"We will insist that there is a patient guide…an information sheet for the patient…It [PIL] will talk about the relative inexperience of the team, saying that this is new to us, but that we're doing it under conditions where we've got an expert coming to help us, we've been to appropriate training, it's been approved by the organisation as something that we think will be safe, but that it is a new way of doing things." (Governance representative 24, Director for Quality Improvement, Male, Trust 9)

However, other trusts had no mandatory requirements for written information or did not review written documentation as part of the application to introduce a new procedure:

"Obviously in situations in which the technique might be new then that's (PIL) not something I've specifically addressed but probably should do." (Governance Representative 48, Clinical Effectiveness Lead, Male, Trust 24)

"I definitely think they should know it's a new procedure. The way we probably tell (patients) is only in the consent process verbally, I don't recall seeing that in any of our written leaflets." (Governance representative 47, Head of governance, Female, Trust 18)

The challenges of discussing innovation

As previously discussed, surgeons felt a need to deliver additional information during consultations for innovative surgery. However, data suggest that some surgeons may find this challenging:

"it's just trying to get across not only this fact that this is what it is, but the fact that it's newer than something else which as a standard we use. That's quite difficult trying to get that across, and some patients will understand better than others." (Surgeon 42, Neurosurgery, Male, Trust 13)

For peer review only - http://bmjopen.bmj.com/site/about/guidelines.xhtml

At a practical level, surgeons reported finding it difficult to establish what information is essential and how much detail is enough. These issues may be further compounded when faced with time constraints, which limit surgeons' ability to have long and/or multiple discussions with patients:

"We don't have much time …I'm finding that I'm over talking far too much information, but I need to make them aware that they're essentially guinea pigs." (Surgeon 23, Colorectal, Female, Trust 2)

Delivering information to a variety of patients who may differ in their ability to understand complex - and potentially very technical - information was a further challenge. To help overcome this, some surgeons felt it was important to simplify technical information and, when possible, provide alterative mediums of information to aid decision making:

"It's [surgical procedure] such a complex thing to be explaining to people so you have to try and bring it down to simplistic terms but at the same time give it in a way that actually they can process the salient points of the information." (Surgeon 46, Trauma & Orthopaedic, Male, Trust 22)

"I think one of the most important things about when you're making this change is that you give people different forms of access, and of course now it's easy. There are YouTube videos of stuff." (Surgeon 43, Orthopaedic, Male, Trust 2)

Surgeons acknowledged that innovative surgery was associated with uncertainties, including a lack of knowledge surrounding risks, benefits and long-term outcomes. Some felt that discussing uncertainties may conflict with the professional norms within surgery:

"This is what surgeons find hard. You have to say, "I don't know," and we hate that. But actually, we should say it more often. We should say it more often." (Surgeon 4, General and upper gastrointestinal, Male, Trust 12)

As a result, when faced with uncertainty surrounding an innovative surgery, surgeons sometimes appeared to disclose their personal opinions during patient consultations:

"They were told that it was a new technique, that we weren't sure what the long-term outcomes were going to be but, we thought that it was better than traditional surgery." (Surgeon 16, Urology, Male, Trust 2)

On the other hand, some expressed a desire to remain impartial and stressed the importance of providing balanced information. Several interviewees described using strategies to help ensure they remained as objective as possible during consultations. Examples discussed included adhering to scripted information and only discussing innovative surgery if a patient specifically requested an alternative to conventional approaches:

For peer review only - http://bmjopen.bmj.com/site/about/guidelines.xhtml

"You can word a new procedure, this is amazing, fantastic, amazing, it's phenomenal and there's no risks to it, and all the patients will say sure, yeah sign me up. Whereas, this is what we're trying to look for, this is the benefits, these are the potential risks, so on and so forth. Then it becomes much more objective." (Surgeon 25, Thoracic, Male, Trust 14)

Of note however, the language used to describe conventional surgical approaches during interviews highlights the potential for surgeons – albeit unintentionally – to influence treatment choice. For instance, terms such as 'old fashioned' and 'traditional' were used by some when describing how they approached patient information provision:

"I'll tell them, "We're going to try and do it like this. Is that okay? Do you have any objection? The alternative is we're going to do it the old-fashioned way, which is make a big cut in your- on your stomach wall" (Surgeon 4, General & Upper Gastrointestinal Surgeon, Male Trust 12)

Managing patient expectations

Surgeons felt that patients often had preferences for new treatments and expected these to deliver better outcomes. For instance those with experience of introducing robotically assisted surgery commonly reported that patients conveyed a strong preference for this approach. Consistent with this, one interviewee felt that patient demand for robotically assisted surgery was a driving factor for hospitals to invest in robotics.

"Patients took to it [robotically assisted surgery] very well. I thought they might be a bit nervous about it, you know the robot taking over or going crazy…patients like the idea of technology. They equate the robot to being an advanced, superior treatment." (Surgeon 31, Colorectal, Male, Trust 8)

"Patients wanted to have robotic surgery, and if you didn't have a robot, the patients would just go to a neighbouring hospital that did. So, it's really patient driven, so if you wanted to be a centre you have to have a robot." (Surgeon 16, Urology, Male, Trust 2)

In general, interviewees felt patients held a misconception that 'new' surgical procedures and technologies are better than those normally provided. Some surgeons reported that patients had actively sought them out in order to receive innovative surgery:

"They tend to have sought you out, because they want something different than the standard. So they're kind of are willing to accept uncertainty and risk because they've already sought out that uncertainty and risk, if you know what I mean." (Surgeon 9, Upper gastrointestinal, Male, Trust 3)

For peer review only - http://bmjopen.bmj.com/site/about/guidelines.xhtml

Several surgeons were concerned that patients did not understand that innovative procedures may carry uncertainties in terms of risk and benefit. Managing patients' preferences, particularly if they align with the personal preferences of the surgeon, appeared challenging for some:

> *"When does a patient turn around and say, 'Well I'll have the old one'? And the answer is never, and that's difficult, because they are immediately signing themselves up for an experiment. And how you describe that to patients is really difficult, because you've got all of your intrinsic biases yourself. Okay, go on then." (Surgeon 43, Orthopaedic, Male, Trust 2)*

> *"I don't think I've had anyone say, 'no, thank you'. I think most of them have gone, 'I don't mind being a guinea pig'. They always use that phrase (laughter). Makes me feel a little bit uncomfortable." (Surgeon 36, Breast, Female, Trust 4)*

Discussion

This study provides novel insights into surgeons' and governance representatives' views on information provision in the context of innovative surgical procedures and represents a significant contribution to the limited empirical literature. Results highlight inconsistencies in how NHS trusts govern how patients should be informed about innovative surgical procedures. Furthermore, interviews with surgeons suggest a lack of consistency in amount and quality of the provision of verbal and written information prior to delivery of innovative surgical treatment. Data indicate a heavy reliance on surgeons to decide what and how information is communicated to patients within some trusts. This conflicts with the recommendations published by the RCSEng, which suggest oversight committees should discuss information provision and consent with surgeons prior to approvals being granted [9]. Collectively, our results stress the need for better oversight and regulation of information provision in the context of innovative surgery. Without improved standards of information provision, patients' informed consent is potentially compromised. There is, however, particular complexity in understanding what innovation is, and when it might occur, that makes establishing these standards difficult.

Consistent with previous research, our findings suggest that surgeons may struggle to clearly define surgical innovation[50], and this may impact upon their approach to patient information provision and consent. It can represent a continuum from standard practice through to true innovation. Rogers et al [14] demonstrated that surgeons held no uniform view of surgical innovation, and showed the lack of agreement on the distinction between innovation and research. Our study highlights the implications of ambiguity – surgeons may use their own judgement as to what is innovative and therefore not discuss novel components of an operation with patients. In addition, procedures that

For peer review only - http://bmjopen.bmj.com/site/about/guidelines.xhtml

were deemed innovative were sometimes introduced as part of routine clinical practice. This meant that information provided to patients was at the discretion of the surgeon, rather than an ethics review board and no peer review of the process. Birchley et al have suggested that instead of focusing on whether something is 'innovative' or not, the potential risks and the ethical appropriateness of modifying surgical practice should be considered to ensure the safe translation of surgical innovation into clinical practice[24]. Work is ongoing to better conceptualise what innovation is and determine when it is happening [51] - although in the meantime we rely on surgeons' views on what is innovative and how that influences their practice of information provision.

Prior research has shown patients do not always feel fully informed about the different treatment options available to them[52 53]. It is likely that the added complexity associated with innovative treatments (e.g. unknown unknown risks and benefits, lack of long-term data, limited surgeon experience of this technique) makes discussing treatments options particularly challenging for surgeons. This has an impact on the process of information provision within the context of gaining informed consent to undergo the procedure. As many innovations are undertaken within the context of clinical practice and not research, then the information provision is at the discretion of the surgeon. Study participants recognised the importance of communicating the risks and benefits, treatment alternatives, and novel status of a procedure to patients. However, it was less common for interviewees to identify prior training, experience with undertaking the procedure, and the evidence base supporting the innovation as important components of consent in information provision. These elements of information provision are recognised as key to gain informed consent [2 9]. The variation observed in this study suggests there is a lack of clarity in guidance for surgeons on what should be disclosed during the content of informed consent discussions in the context of innovative surgery, meaning that some patients are likely to be better informed than others when making treatment decisions. This is a concern given that information provision within the context of informed consent has been linked to patient satisfaction and rates of litigation following surgery[54-56].

Aligning with previous research, our data suggests surgeons may not disclose their personal experience of performing a procedure to patients and this is important to patients[57]. In this study few surgeons identified, or reported discussing, surgical experience and/or training as a component of information provision. While we cannot conclude from the current data, the disclosure of this information could lead to conversations regarding the surgical learning curve. Such discussions could help emphasise to patients that a procedure is novel and there are potential uncertainties in terms of risks and benefits. Training programmes have been developed to enhance informed consent in RCTs[58 59], although none have focused on innovative surgical procedures. Our study suggests that

For peer review only - http://bmjopen.bmj.com/site/about/guidelines.xhtml

surgeons may require support and training to raise awareness of the challenges of discussing novel procedures and provide support to overcome these.

To the best of our knowledge, this study is the first to explore healthcare professionals' views of what information should be shared with patients during informed consent consultations in the context of innovative surgery. An important strength of the study is the sample of both surgeons and governance representatives, recruited from various surgical specialities, trust types and geographical locations. This allowed us to explore information provisioned consent from two different perspectives, and provided important insight into the role trust governance plays in patient information provision and consent. Nonetheless, there are potential limitations to the study that should be acknowledged. We cannot conclude exactly what information is communicated to patients during consultations from interview data alone. Future research should capture what actually happens in these interactions by audio recording clinician-patient consultations. Such methods have been used successfully in RCTs to improve the quality of informed consent and recruitment rates[60-62]. Furthermore, studies exploring patients' views, particularly those who are offered or have received innovative surgery, are critical if we are to understand the type of information patients draw upon when making decisions about their care. Such work is currently being conducted by our research group as part of a larger research study exploring innovation in surgery[51]. It is hoped that this research will aid the development of 'core information sets' (minimum sets of information that should be discussed with patients), which will ultimately support surgeons to discuss innovation and help catalyse the discussion of issues that are important to individual patients[63].

In conclusion, the study suggests that information provision for innovative surgery presents a challenge for surgeons. Our findings also highlight variation in how NHS trusts govern information provision and consent practice for innovative surgery, and further inconsistencies between the views of surgeons and governance representatives. When innovations are introduced under the auspices of research, there are governance structures (e.g. research ethics committees) in place to help ensure patient information is appropriate. However, innovation and modifications to surgery often takes place without formal research approval, which raises the question of how well informed patients are prior to surgery. While the recent RCSEng guidelines for introducing new techniques and technologies represents an important step towards improving informed consent, our study suggests a need to develop support and training programmes to ensure that surgeons consistently deliver appropriate and comprehensive information to patients in the context of innovative surgery.

<u>Authors' contributions</u>

For peer review only - http://bmjopen.bmj.com/site/about/guidelines.xhtml

JMB conceptualised the idea to study informed consent and information provision in the context of surgical innovation. DE developed the idea for the current study with support from JMB, JZ, SP, SC, NB, BM, AM and KA. NB, JM, AM and BM identified potential participants. JZ and DE conducted the interviews and analysed the data, with regular discussion of data interpretation with SP and JMB. The NIHR Bristol Biomedical Research Centre Surgical Innovation theme is directed by JMB (theme lead) and RH (deputy theme lead). The manuscript was written by JZ, and DE critically reviewed the manuscript and responded to reviewers' comments. All authors commented on the article.

For peer review only

For peer review only - http://bmjopen.bmj.com/site/about/guidelines.xhtml

Disclaimer

The views expressed in this publication are those of the authors and not necessarily those of the NHS, the National Institute for Health Research, the MRC or the Department of Health and Social Care.

Competing interests

None declared

Data availability statement:

All data relevant to the study are included in the article or uploaded as supplementary information

References

1. Committee BvFHM. All ER 118,[1957] 1 WLR 582.
2. Surgeons RCo. Consent: Supported Decision-Making-a good practice guide: RCS London, 2016.
3. Coulter A, Hopkins A, Moulton B. Montgomery v Lanarkshire Health Board: transforming informed consent. *The Bulletin of the Royal College of Surgeons of England* 2017;99(1):36-38.
4. Faden RR, Beauchamp TL. A history and theory of informed consent: Oxford University Press 1986.
5. Chan SW, Tulloch E, Cooper ES, et al. Montgomery and informed consent: where are we now? 2017;357:j2224.
6. Chan SW, Tulloch E, Cooper ES, et al. Montgomery and informed consent: where are we now? 2017;357:j2224. doi: 10.1136/bmj.j2224 %J BMJ
7. Royal College of Surgeons. Learning from invited reviews. 2019
8. Dyer C. Robot assisted surgery is blamed for heart patient's death. 2018;363:k4791. doi: 10.1136/bmj.k4791 %J BMJ
9. Royal College of Surgeons. Surgical innovation, new techniques and technologies 2019.
10. Bracken-Roche D, Bell E, Karpowicz L, et al. Disclosure, Consent, and the Exercise of Patient Autonomy in Surgical Innovation: A Systematic Content Analysis of the Conceptual Literature. *Accountability in Research* 2014;21(6):331-52. doi: 10.1080/08989621.2013.866045
11. Di Paolo M, Boggi U, Turillazzi E. Bioethical approach to robot-assisted surgery. 2019
12. Reitsma A, Moreno J. *J Am Coll Surg* 2002;194(null):792.
13. Char SJL, Hills NK, Lo B, et al. Informed consent for innovative surgery: A survey of patients and surgeons. *Surgery* 2013;153(4):473-80. doi: https://doi.org/10.1016/j.surg.2012.08.068
14. Rogers WA, Lotz M, Hutchison K, et al. Identifying surgical innovation: a qualitative study of surgeons' views. *Ann Surg* 2014;259(2):273-78.
15. Apramian T, Watling C, Lingard L, et al. Adaptation and innovation: a grounded theory study of procedural variation in the academic surgical workplace. *J Eval Clin Pract* 2015;21(5):911-18. doi: 10.1111/jep.12398
16. Danjoux NM, Martin DK, Lehoux PN, et al. Adoption of an innovation to repair aortic aneurysms at a Canadian hospital: a qualitative case study and evaluation. *BMC Health Serv Res* 2007;7(1):182. doi: 10.1186/1472-6963-7-182
17. Sharma B, Danjoux NM, Harnish JL, et al. How are decisions to introduce new surgical technologies made? Advanced laparoscopic surgery at a Canadian community hospital: A qualitative case study and evaluation. *Surg Innov* 2006;13(4):250-6. doi: 10.1177/1553350606296341 [published Online First: 2007/01/18]

For peer review only - http://bmjopen.bmj.com/site/about/guidelines.xhtml

18. Johnson J, Hutchison K. They Know How to Work It, That's Their Focus in Life: The Complex Role of Industry Representatives in Surgical Innovation. *Journal of Empirical Research on Human Research Ethics* 2018;13(5):461-74. doi: 10.1177/1556264618785037
19. Cousins S, Richards H, Zahra J, et al. Introduction and adoption of innovative invasive procedures and devices in the NHS: an in-depth analysis of written policies and qualitative interviews (the INTRODUCE study protocol). 2019;9(8):e029963. doi: 10.1136/bmjopen-2019-029963 %J BMJ Open
20. Health Do. Health Service Circular (HSC2003/011) – The Interventional Procedures Programme 2003 [Available from: https://webarchive.nationalarchives.gov.uk/20120503190439/http://www.dh.gov.uk/prod_consum_dh/groups/dh_digitalassets/@dh/@en/documents/digitalasset/dh_4064925.pdf accessed 12/02/2018.
21. Reitsma AM, Moreno JD. *The American Journal of Bioethics* 2003;3(null):49.
22. Johnson J, Rogers W. *J Med Ethics* 2011;38(null):9.
23. Hutchison K, Rogers W, Eyers A, et al. Getting Clearer about surgical innovation: a new definition and a new tool to support responsible practice. *Ann Surg* 2015;262(6):949-54.
24. Birchley G, Huxtable R, Ives J, et al. Have We Made Progress in Identifying (Surgical) Innovation? *The American Journal of Bioethics* 2019;19(6):25-27. doi: 10.1080/15265161.2019.1602181
25. O'Brien BC, Harris IB, Beckman TJ, et al. Standards for reporting qualitative research: a synthesis of recommendations. *Academic medicine : journal of the Association of American Medical Colleges* 2014;89(9):1245-51. doi: 10.1097/acm.0000000000000388 [published Online First: 2014/07/01]
26. Patton MQJQsw. Two decades of developments in qualitative inquiry: A personal, experiential perspective. 2002;1(3):261-83.
27. Dempster M. A Research Guide for Health and Clinical Psychology: Palgrave Macmillan 2011.
28. Fassinger REJJocp. Paradigms, praxis, problems, and promise: Grounded theory in counseling psychology research. 2005;52(2):156.
29. Sandelowski MJRin, health. Sample size in qualitative research. 1995;18(2):179-83.
30. Braun V, Clarke V. Successful qualitative research: A practical guide for beginners: sage 2013.
31. Bowen GA. Naturalistic inquiry and the saturation concept: a research note. 2008;8(1):137-52. doi: 10.1177/1468794107085301
32. Smith JAJRmip. Semi-structured interviewing and qualitative analysis. 1995
33. Britten NJB. Qualitative research: qualitative interviews in medical research. 1995;311(6999):251-53.
34. Rubin HJ, Rubin IS. Qualitative interviewing: The art of hearing data: sage 2011.
35. Hammersley P, Atkinson M. Ethnography: principles and practice. London: Routledge 1983.
36. Willig C. Applied discourse analysis: Social and psychological interventions: Open University Press 1999.
37. Burman E. Minding the Gap: Positivism, Psychology, and the Politics of Qualitative Methods. 1997;53(4):785-801. doi: 10.1111/j.1540-4560.1997.tb02461.x
38. Brinkmann S, Kvale S. Interviews: Learning the craft of qualitative research interviewing: Sage Thousand Oaks, CA 2015.
39. Fielding N, Thomas H. Qualitative interviewing. 2008
40. Charmaz K, Belgrave LJTShoirTcotc. Qualitative interviewing and grounded theory analysis. 2012;2:347-65.
41. Glaser BG, Strauss AL. The Discovery of Grounded Theory. Aldine Transaction: USA 1967.
42. Seale C, Silverman DJTEJoPH. Ensuring rigour in qualitative research. 1997;7(4):379-84.
43. Krippendorff KJHcr. Reliability in content analysis: Some common misconceptions and recommendations. 2004;30(3):411-33.
44. Miles MB, Huberman AM, Huberman MA, et al. Qualitative data analysis: An expanded sourcebook: sage 1994.

For peer review only - http://bmjopen.bmj.com/site/about/guidelines.xhtml

45. Pope C, Ziebland S, Mays N. Analysing qualitative data. *BMJ* 2000;320(7227):114-16. doi: 10.1136/bmj.320.7227.114
46. Braun V, Clarke V. Using thematic analysis in psychology. *Qualitative Research in Psychology* 2006;3(2):77-101. doi: 10.1191/1478088706qp063oa
47. Charmaz K. Constructing grounded theory: A practical guide through qualitative analysis: sage 2006.
48. Barbour RSJB. Checklists for improving rigour in qualitative research: a case of the tail wagging the dog? 2001;322(7294):1115-17.
49. Dey I. Qualitative data analysis: A user friendly guide for social scientists: Routledge 2003.
50. Rogers WA, Lotz M, Hutchison K, et al. Identifying surgical innovation: a qualitative study of surgeons' views. *Ann Surg* 2014;259(2):273-8. doi: 10.1097/SLA.0b013e31829ccc5f [published Online First: 2013/06/22]
51. McNair AGK, Main BJ, Elliott D. Comment on: Bioethical approach to robot-assisted surgery. 2020;107(1):150-50. doi: 10.1002/bjs.11413
52. Mitchell S, Gass J, Hanna M. How Well Informed Do Patients Feel about Their Breast Cancer Surgery Options? Findings from a Nationwide Survey of Women after Lumpectomy and/or Mastectomy. *Journal of the American College of Surgeons* 2018;226(2):134-46.e3. doi: 10.1016/j.jamcollsurg.2017.10.022 [published Online First: 2017/12/17]
53. Kinnersley P, Phillips K, Savage K, et al. Interventions to promote informed consent for patients undergoing surgical and other invasive healthcare procedures. 2013(7)
54. Ford KE, Cooper LRL. Learning from lawsuits: Ten-years of NHS litigation authority claims against 11 surgical specialities in England. *The Surgeon* 2018;16(1):27-35. doi: https://doi.org/10.1016/j.surge.2016.03.009
55. Veerman MM, van der Woude LA, Tellier MA, et al. A decade of litigation regarding surgical informed consent in the Netherlands. *Patient Education and Counseling* 2019;102(2):340-45. doi: https://doi.org/10.1016/j.pec.2018.08.031
56. Hallock JL, Rios R, Handa VL. Patient satisfaction and informed consent for surgery. *American journal of obstetrics and gynecology* 2017;217(2):181.e1-81.e7. doi: 10.1016/j.ajog.2017.03.020 [published Online First: 2017/04/02]
57. Lee Char SJ, Hills NK, Lo B, et al. Informed consent for innovative surgery: a survey of patients and surgeons. *Surgery* 2013;153(4):473-80. doi: 10.1016/j.surg.2012.08.068 [published Online First: 2012/12/04]
58. Townsend D, Mills N, Savović J, et al. A systematic review of training programmes for recruiters to randomised controlled trials. *Trials* 2015;16:432. doi: 10.1186/s13063-015-0908-6
59. Elliott D, Husbands S, Hamdy FC, et al. Understanding and Improving Recruitment to Randomised Controlled Trials: Qualitative Research Approaches. *Eur Urol* 2017;72(5):789-98. doi: https://doi.org/10.1016/j.eururo.2017.04.036
60. Elliott D, Husbands S, Hamdy FC, et al. Understanding and Improving Recruitment to Randomised Controlled Trials: Qualitative Research Approaches. *European urology* 2017;72(5):789-98. doi: 10.1016/j.eururo.2017.04.036 [published Online First: 2017/06/06]
61. Rooshenas L, Paramasivan S, Jepson M, et al. Intensive Triangulation of Qualitative Research and Quantitative Data to Improve Recruitment to Randomized Trials: The Quintet Approach. 2019;29(5):672-79.
62. Donovan JL, Rooshenas L, Jepson M, et al. Optimising recruitment and informed consent in randomised controlled trials: the development and implementation of the Quintet Recruitment Intervention (QRI). *Trials* 2016;17(1):283. doi: 10.1186/s13063-016-1391-4
63. Main BG, McNair AGK, Huxtable R, et al. Core information sets for informed consent to surgical interventions: baseline information of importance to patients and clinicians. *BMC medical ethics* 2017;18(1):29. doi: 10.1186/s12910-017-0188-7

For peer review only - http://bmjopen.bmj.com/site/about/guidelines.xhtml

Reporting checklist for qualitative study.

Based on the SRQR guidelines.

Instructions to authors

Complete this checklist by entering the page numbers from your manuscript where readers will find each of the items listed below.

Your article may not currently address all the items on the checklist. Please modify your text to include the missing information. If you are certain that an item does not apply, please write "n/a" and provide a short explanation.

Upload your completed checklist as an extra file when you submit to a journal.

In your methods section, say that you used the SRQRreporting guidelines, and cite them as:

O'Brien BC, Harris IB, Beckman TJ, Reed DA, Cook DA. Standards for reporting qualitative research: a synthesis of recommendations. Acad Med. 2014;89(9):1245-1251.

		Reporting Item	Page Number
**Title**			
	#1	Concise description of the nature and topic of the study identifying the study as qualitative or indicating the approach (e.g. ethnography, grounded theory) or data collection methods (e.g. interview, focus group) is recommended	1
**Abstract**			
	#2	Summary of the key elements of the study using the abstract format of the intended publication; typically includes background, purpose, methods, results and conclusions	2
**Introduction**			
Problem formulation	#3	Description and signifcance of the problem / phenomenon studied: review of relevant theory and empirical work; problem statement	4

For peer review only

Purpose or research question	#4	Purpose of the study and specific objectives or questions	5
**Methods**			
Qualitative approach and research paradigm	#5	Qualitative approach (e.g. ethnography, grounded theory, case study, phenomenolgy, narrative research) and guiding theory if appropriate; identifying the research paradigm (e.g. postpositivist, constructivist / interpretivist) is also recommended; rationale. The rationale should briefly discuss the justification for choosing that theory, approach, method or technique rather than other options available; the assumptions and limitations implicit in those choices and how those choices influence study conclusions and transferability. As appropriate the rationale for several items might be discussed together.	7
Researcher characteristics and reflexivity	#6	Researchers' characteristics that may influence the research, including personal attributes, qualifications / experience, relationship with participants, assumptions and / or presuppositions; potential or actual interaction between researchers' characteristics and the research questions, approach, methods, results and / or transferability	6; 8
Context	#7	Setting / site and salient contextual factors; rationale	6
Sampling strategy	#8	How and why research participants, documents, or events were selected; criteria for deciding when no further sampling was necessary (e.g. sampling saturation); rationale	5-6
Ethical issues pertaining to human subjects	#9	Documentation of approval by an appropriate ethics review board and participant consent, or explanation for lack thereof; other confidentiality and data security issues	5
Data collection methods	#10	Types of data collected; details of data collection procedures including (as appropriate) start and stop	5-7

For peer review only

		dates of data collection and analysis, iterative process, triangulation of sources / methods, and modification of procedures in response to evolving study findings; rationale	
Data collection instruments and technologies	#11	Description of instruments (e.g. interview guides, questionnaires) and devices (e.g. audio recorders) used for data collection; if / how the instruments(s) changed over the course of the study	6-7
Units of study	#12	Number and relevant characteristics of participants, documents, or events included in the study; level of participation (could be reported in results)	8
Data processing	#13	Methods for processing data prior to and during analysis, including transcription, data entry, data management and security, verification of data integrity, data coding, and anonymisation / deidentification of excerpts	7-8
Data analysis	#14	Process by which inferences, themes, etc. were identified and developed, including the researchers involved in data analysis; usually references a specific paradigm or approach; rationale	7-8
Techniques to enhance trustworthiness	#15	Techniques to enhance trustworthiness and credibility of data analysis (e.g. member checking, audit trail, triangulation); rationale	7-8
**Results/findings**			
Syntheses and interpretation	#16	Main findings (e.g. interpretations, inferences, and themes); might include development of a theory or model, or integration with prior research or theory	8-14
Links to empirical data	#17	Evidence (e.g. quotes, field notes, text excerpts, photographs) to substantiate analytic findings	8-14
**Discussion**			
Intergration with prior work, implications,	#18	Short summary of main findings; explanation of how findings and conclusions connect to, support, elaborate on, or challenge conclusions of earlier	15-17

For peer review only

transferability and contribution(s) to the field		scholarship; discussion of scope of application / generalizability; identification of unique contributions(s) to scholarship in a discipline or field	
Limitations	#19	Trustworthiness and limitations of findings	16
**Other**			
Conflicts of interest	#20	Potential sources of influence of perceived influence on study conduct and conclusions; how these were managed	N/A
Funding	#21	Sources of funding and other support; role of funders in data collection, interpretation and reporting	17

The SRQR checklist is distributed with permission of Wolters Kluwer © 2014 by the Association of American Medical Colleges. This checklist was completed on 12. February 2020 using https://www.goodreports.org/, a tool made by the EQUATOR Network in collaboration with Penelope.ai

For peer review only

Topic guide for interviews with governance representatives

Introduction:

- Thank for taking time
- Not medical professional, want to understand process of implementing new procedures and devices
- No right or wrong answers, can stop the interview at any time
- Take verbal consent

Can you tell me about yourself?

- What is your current position and role?
- Particular areas of interest/expertise?
- How did you become involved in [committee]? When? How found it? What you expected?
- Can you tell me about the committee? What is the role of the committee?
    - Who involved? How often meet?

As you know, we are looking at innovation in surgery. Can you talk me through how you would define innovation? *(Newness; degree of change, level of risk, impact?)*

Can you talk me through how applications are processed, from submissions to decision-making?

- What is the process for introducing surgical innovation in [site]?
- Do you think there are similarities/difference between innovation and research? In what ways?
- How many applications do you receive each year?
- What information is provided? *(What is compulsory?)*
- How are they considered?
    - Potential benefit? Safety (and how quantified?) Evidence? Cost? Patient acceptability?
- Why would an application be declined? Example? Agreement between committee members?
- What happens to those procedures that are declined? Is there an option to appeal?

What's your understanding of how these procedures are integrated into practice?

- What happens once a technique/device is approved by the committee?
- If approved, is there any monitoring? How? When?
- What if there is a problem with a novel procedure?
- Is there any guidance for communicating about novel procedures?
- What do you think patients are told about the novel component? What should they be told - What aspects important to communicate?
- What is included on patient info sheets provided

Your views on the committee

- How do you feel about being involved in the decision-making process?
- Are there any particularly challenging aspects?
- What are the strengths of the committee?
- Do you think it could be improved in anyway?

Ending the interview

- Is there anything else that I haven't mentioned?
- Anyone else you recommend I could contact?

For peer review only

- Before we finish (if not covered) – role (registrar/consultant), years in your profession, highest qualification

For peer review only

Topic guide for interviews with surgeons

Introduction:

- Thank you for taking time
- Not medical professional, want to understand perspectives of innovation
- No right or wrong answers, can stop the interview at any time
- Take verbal consent

Can you tell me about yourself?

- What is your current position and role?
- Can you describe the range /type(s) of surgical procedures you undertake?
- Particular areas of interest/expertise?

As you know, we are looking at innovation in surgery. Can you talk me through how you would define innovation? *(Newness; degree of change, level of risk, impact?)*

Have you conceptualised a new procedure/device/modification of a procedure?

- How did it come about? When?
- Would you regard this as an innovation or a modification?
- Why do you feel it's needed?
- Who is suitable for the procedure? *(How determined?)*
- What treatment options are currently available to them? What happens if patients don't get the procedure? (Are they disadvantaged in any way?)
- What do you hope will be the outcomes from the new procedure? How estimated?

Do you think patients should be informed about new procedures/devices?

- What does 'informed consent' mean to you?
- Is there any guidance for communicating novel procedure?
- What should they be told? Extent that procedure is novel? Uncertainty? Risks? Harms?
- Is informing patients about a new procedure challenging in anyway?
- How do you think patients will respond to the procedure?
- How much of a modification would you need to do to tell a patient something was new?
- When do you stop telling them it is new?

Looking ahead

- Do you think any subgroups of patients would be more suited to the procedure? Are there any that you would feel less comfortable with having the procedure?
- Do you have any concerns about the procedure? Risks to patients? Costs?
- Do you have any plans to disseminate the results? How? All results (good and bad)? Where? When? Who's responsible for this?

Ending the interview

- Is there anything else that I haven't mentioned?
- Anyone else you recommend I could contact?
- Thank for time, summarise key points.

For peer review only

BMJ Open

Discussing surgical innovation with patients: A qualitative study of surgeons' and governance representatives' views

Journal:	*BMJ Open*
Manuscript ID	bmjopen-2019-035251.R3
Article Type:	Original research
Date Submitted by the Author:	18-Aug-2020
Complete List of Authors:	Zahra, Jesmond; University of Bristol, National Institute for Health Research Bristol Biomedical Research Centre Surgical Innovation Theme, Bristol Centre for Surgical Research, Bristol Medical School: Population Health Sciences, University of Bristol<br>Paramasivan, Sangeetha ; University of Bristol, National Institute for Health Research Bristol Biomedical Research Centre Surgical Innovation Theme, Bristol Centre for Surgical Research, Bristol Medical School: Population Health Sciences, University of Bristol<br>Blencowe, Natalie; University of Bristol, National Institute for Health Research Bristol Biomedical Research Centre Surgical Innovation Theme, Bristol Centre for Surgical Research, Bristol Medical School: Population Health Sciences, University of Bristol; University Hospitals Bristol NHS Foundation Trust, Division of Surgery<br>Cousins, Sian; University of Bristol, National Institute for Health Research Bristol Biomedical Research Centre Surgical Innovation Theme, Bristol Centre for Surgical Research, Bristol Medical School: Population Health Sciences, University of Bristol<br>Avery, Kerry; University of Bristol, National Institute for Health Research Bristol Biomedical Research Centre Surgical Innovation Theme, Bristol Centre for Surgical Research, Bristol Medical School: Population Health Sciences, University of Bristol<br>Mathews, Johnny; University of Bristol, National Institute for Health Research Bristol Biomedical Research Centre Surgical Innovation Theme, Bristol Centre for Surgical Research, Bristol Medical School: Population Health Sciences, University of Bristol<br>Main, Barry; University of Bristol, National Institute for Health Research Bristol Biomedical Research Centre Surgical Innovation Theme, Bristol Centre for Surgical Research, Bristol Medical School: Population Health Sciences, University of Bristol; University Hospitals Bristol NHS Foundation Trust<br>McNair, Angus; University of Bristol, National Institute for Health Research Bristol Biomedical Research Centre Surgical Innovation Theme, Bristol Centre for Surgical Research, Bristol Medical School: Population Health Sciences, University of Bristol; North Bristol NHS Trust, GI Surgery<br>Hinchliffe, Robert; University of Bristol, National Institute for Health Research Bristol Biomedical Research Centre Surgical Innovation Theme, Bristol Centre for Surgical Research, Bristol Medical School: Population Health Sciences, University of Bristol; North Bristol NHS Trust, Vascular Services<br>Blazeby, Jane; University of Bristol, National Institute for Health

	Research Bristol Biomedical Research Centre Surgical Innovation Theme, Bristol Centre for Surgical Research, Bristol Medical School: Population Health Sciences, University of Bristol; University Hospitals Bristol NHS Foundation Trust<br>Elliott, Daisy; University of Bristol, National Institute for Health Research Bristol Biomedical Research Centre Surgical Innovation Theme, Bristol Centre for Surgical Research, Bristol Medical School: Population Health Sciences, University of Bristol
**Primary Subject Heading**:	Surgery
Secondary Subject Heading:	Communication
Keywords:	SURGERY, Health policy < HEALTH SERVICES ADMINISTRATION & MANAGEMENT, QUALITATIVE RESEARCH

SCHOLARONE™
Manuscripts

I, the Submitting Author has the right to grant and does grant on behalf of all authors of the Work (as defined in the below author licence), an exclusive licence and/or a non-exclusive licence for contributions from authors who are: i) UK Crown employees; ii) where BMJ has agreed a CC-BY licence shall apply, and/or iii) in accordance with the terms applicable for US Federal Government officers or employees acting as part of their official duties; on a worldwide, perpetual, irrevocable, royalty-free basis to BMJ Publishing Group Ltd ("BMJ") its licensees and where the relevant Journal is co-owned by BMJ to the co-owners of the Journal, to publish the Work in this journal and any other BMJ products and to exploit all rights, as set out in our licence.

The Submitting Author accepts and understands that any supply made under these terms is made by BMJ to the Submitting Author unless you are acting as an employee on behalf of your employer or a postgraduate student of an affiliated institution which is paying any applicable article publishing charge ("APC") for Open Access articles. Where the Submitting Author wishes to make the Work available on an Open Access basis (and intends to pay the relevant APC), the terms of reuse of such Open Access shall be governed by a Creative Commons licence – details of these licences and which Creative Commons licence will apply to this Work are set out in our licence referred to above.

Other than as permitted in any relevant BMJ Author's Self Archiving Policies, I confirm this Work has not been accepted for publication elsewhere, is not being considered for publication elsewhere and does not duplicate material already published. I confirm all authors consent to publication of this Work and authorise the granting of this licence.

For peer review only

Discussing surgical innovation with patients: A qualitative study of surgeons' and governance representatives' views

Submission category: Original article

J Zahra[1], S Paramasivan[1], N.S Blencowe[1,3], S Cousins[1], K Avery[1], J Mathews[1,2], B Main [1,3], A McNair [1,2], R Hinchliffe[1,2], J.M Blazeby[1,3], D Elliott[1]

[1]National Institute for Health Research Bristol Biomedical Research Centre Surgical Innovation Theme, Centre for Surgical Research, Population Health Sciences, Bristol Medical School, University of Bristol, 39 Whatley Road, Clifton, Bristol, UK. BS8 2PS;

[2]North Bristol NHS Trust, Bristol, UK. BS10 5NB.

[3]University Hospitals Bristol NHS Foundation Trust, Bristol, UK. BS2 8HW

Running title: Discussing surgical innovation with patients

Keywords: consent, surgery, information provision, innovation, qualitative,

Corresponding author details: Daisy Elliott [daisy.elliott@bristol.ac.uk]

Funding statement: This study was funded by the NIHR Biomedical Research Centre at University Hospitals Bristol and Weston NHS Foundation Trust and the University of Bristol, with support from the MRC ConDuCT-II (Collaboration and innovation for Difficult and Complex randomised controlled Trials In Invasive procedures) Hub for Trials Methodology Research (MR/K025643/1). The views expressed are those of the author(s) and not necessarily those of the NIHR or the MRC. BM is a NIHR Clinical Lecturer, AM is funded by a Clinician Scientist Fellowship (NIHR-CS-2017-17-010) from the National Institute for Health Research, and NB is funded by an MRC Clinician Scientist Award.

Word count: 4380

For peer review only

Abstract

Objectives

Little is known about how innovative surgical procedures are introduced and discussed with patients. This qualitative study aimed to explore perspectives on information provision and consent prior to innovative surgical procedures.

Design

Qualitative study involving semi-structured interviews. Interviews were audio recorded, transcribed and analysed thematically.

Participants

42 interviews were conducted (26 surgeons and 16 governance representatives).

Setting

Surgeons and governance representatives recruited from various surgical specialities and National Health Service Trusts across England, United Kingdom.

Results

Participants stated that if a procedure was innovative, patients should be provided with additional information extending beyond that given during routine surgical consultations. However, difficulty defining innovation had implications for whether patients were informed about novel components of surgery and how the procedure was introduced (i.e. as part of a research study, trust approval or in routine clinical practice). Furthermore, data suggests surgeons found it difficult to establish what information is essential and how much detail is sufficient, and governance surrounding written and verbal information provision differed between NHS trusts. Generally, surgeons believed patients held a view that 'new' was best and reported that managing these expectations could be difficult, particularly if patient views aligned with their own.

Conclusions

This study highlights the challenges of information provision and obtaining informed consent in the context of innovative surgery, including establishing if and how a procedure is truly innovative, determining the key information to discuss with patients, ensuring information provision is objective and balanced, and managing patient expectations and preferences. This suggests that surgeons may require support and training to discussing novel procedures with patients. Further work should

For peer review only - http://bmjopen.bmj.com/site/about/guidelines.xhtml

capture consultations where new procedures are discussed with patients and patients' views of these information exchanges.

For peer review only

For peer review only - http://bmjopen.bmj.com/site/about/guidelines.xhtml

Summary box – strengths and limitations

- This is the first qualitative study to conduct in-depth, semi structured interviews to understand current practice for information provision and informed consent for innovative surgical procedures in the United Kingdom.
- Interviews with governance representatives enabled us to understand what policies were in place for the introduction of new procedures, whilst surgeons provided insights into how they introduced new procedures and discussed these with patients.
- Purposeful sampling ensured that participants were recruited from a range of geographical locations and trust types, and surgeons were from different specialities with varying experiences of innovation.
- Audio-recording appointments where clinicians discuss innovative surgeries and patients decide whether or not to undergo the procedure would strengthen our understanding of interactions between clinicians and patients.
- Further work should capture patients' views on information provision in this context, so that recommendations can be made to improve transparency and communication.

For peer review only

For peer review only - http://bmjopen.bmj.com/site/about/guidelines.xhtml

Introduction

Informed consent is fundamental to patient autonomy and represents a cornerstone of modern healthcare provision. Until recently, the Bolam ruling meant that a doctor's duty to inform patients prior to surgery was judged based on whether they had acted in line with the view of a responsible body of medical opinion [1 2]. The 2015 Montgomery ruling[3] redefined the standard of informed consent in the United Kingdom (UK) and represented a shift from a paternalistic model of consent to a more patient-centred approach[4 5] – meaning that a patient should be told whatever they want to know, not what the doctor thinks they should be told[6].

Recently, important questions have been raised surrounding informed consent for innovative surgical procedures[7 8]. An inquest into the death of the first patient in the UK to undergo robotically assisted heart surgery found the patient had not been fully informed about the comparative risk of robotic versus conventional open surgery[8]. In addition, a summary of invited reviews published by the Royal College of Surgeons of England (RCSEng) raised further concerns[7]. The review highlighted issues surrounding surgeons' ability to accurately quantify risk as reported in published literature, whether surgical experience is being sufficiently disclosed and effectively communicated to patients, and the overall quality of patient-clinician discussions[7]. The RESEng subsequently published guidelines for the development, implementation and dissemination of surgical innovation, which contains guidance on informed consent [9]. This suggests, based on findings of a review by Broekman et al [9], that patients should be informed of the innovative nature of the procedure, surgeons' experience with the procedure and the learning curve, the risk and benefits – including unknown or unforeseeable risks or outcomes, the evidence or lack thereof, and alternatives to the innovative procedure.

Reviews and commentaries have highlighted potential issues around informed consent for innovative surgical procedures, including patients' and surgeons' beliefs that new treatments constitute improved treatment options or result in better outcomes [10 11]. However, little is known about current practice. Surveys that have been conducted in the United States suggest that information varies considerably in content and quality. Reitsma and Moreno (2002) reported that of twenty-one surgeons, 75% verbally informed patients about the innovative nature of the procedure but only 33% provided written information [12]. Char and colleagues (2013), in a scenario based survey carried out with 85 surgeons and 383 patients, found differences between what surgeons thought patients wanted to know about innovative procedures and what patients reported they wanted to know. For instance, compared with surgeons, patients placed more importance on nearly all types of information, particularly volumes and outcomes [13].

For peer review only - http://bmjopen.bmj.com/site/about/guidelines.xhtml

Whilst a handful of qualitative studies have been conducted exploring how innovative procedures have been introduced [14 15 16 17], none have specifically looked at information provision and informed consent. Moreover, these studies were conducted in Australia and Canada and findings may not be generalisable to other countries and different healthcare systems[18]. New invasive procedures may be introduced in the context of formal research studies or via local hospital policies [19]. The National Institute for Health and Care Excellence (NICE) recommends that local NHS organisations have appropriate governance structures in place to review, approve and monitor the introduction of new invasive procedures [20]. The current study sought to explore surgeons' and governance representatives' views of information provision and informed consent for the introduction of new invasive procedures in the United Kingdom (UK).

Methods

Patient and public involvement

Public and patient involvement was not conducted as part of the current study. However, the current findings have been discussed with a recently established group, which has led to further work which specifically aims to explore patient views on surgical innovation.

Design

This qualitative study employed a grounded theory methodology [21] because it enabled the inductive identification of codes from the data to generate new hypotheses about phenomena that are derived or grounded in the data [22]. Its central principle is of constant comparison, where new findings are systematically compared with existing data so that similarities and differences can be identified and emerging theories refined through the ongoing assimilation of data [21 23]. Consistent with grounded theory methodology, semi-structured interviews were conducted with relevant stakeholders.

Ethical approval was granted by the University of Bristol Faculty of Health Sciences Research Ethics Committee. The study is reported in line with the Standards for Reporting Qualitative Research (SRQR; Additional File 1) [24].

Sampling and recruitment

Participants were recruited between November 2017 and October 2018 using a combination of purposive [25]and snowball sampling techniques [26]. Governance representatives (defined as those involved in regulating the introduction of new/modified procedures and/or devices at trust or national levels) were included to understand what processes were in place for the introduction of

For peer review only - http://bmjopen.bmj.com/site/about/guidelines.xhtml

new procedures [17]. Surgeons were recruited to explore experiences of introducing new/modified procedures or devices into clinical practice. As there is no standardised definition for innovation and it is often a continuum from standard practice through to true innovation [14 27 28], and because surgical innovation is unlikely to involve a single discrete development [29 30], participants were asked to reflect what the term meant to them and describe procedures that they deemed to be innovative.

Governance representatives were identified through websites (e.g. National Health Service (NHS) Trusts websites) and NHS policy documents (e.g. new invasive procedure/devices policies). An initial sample of surgeons were identified by academic surgeons working within the Centre for Surgical Research, University of Bristol, UK. Subsequent surgeons were identified via snowball sampling[26], in which interviewees recommended others who may be willing to take part. Sampling was regularly reviewed to ensure surgeons from different specialities and geographical locations, with varying experiences of innovation (e.g. minor modifications vs first in human procedures), were recruited to capture a diversity of perspectives [31 32]. For governance representatives, maximum variation was sought in relation to different roles (e.g. new procedures committee members, medical directors), geographical locations, trust types, and different surgical specialities.

Potential participants were approached via email and up to three invitation emails were sent before contact attempts ceased. In total, 83 potential participants were invited to take part (38 surgeons and 45 governance representatives). Study recruitment ceased when the qualitative team agreed that theoretical saturation had been achieved (where no new themes were forthcoming from three consecutive interviews)[33 34].

Data collection

Interviews were considered to be the most appropriate method for this study because they provided the opportunity to encourage participants to think carefully about their own experiences [35], enable the interviewer to respond and follow up on issues raised by the interviewee [36]. Interviews were chosen instead of focus groups as it was felt that some individuals may feel intimidated at the prospect of discussing their experiences within a focus group setting [26].

All participants received a study information leaflet and provided written consent before taking part. Separate topic guides were developed for governance representatives and surgeons. All interview schedules contained core topics of interest (e.g. 'How would you define innovation?', 'What does informed consent mean to you?')[37 38], although these were flexible to allow participants to shape the direction of conversations[39 40]. Governance representatives were asked how applications for new invasive procedures were processed and how these procedures then integrated into practice,

For peer review only - http://bmjopen.bmj.com/site/about/guidelines.xhtml

whereas surgeons were asked about their experiences of introducing procedures and what patients had been told. An example of each interview schedule is provided in the supplementary files (Additional files 2 and 3).

The interviewer began the discussion with more general questions to build rapport [41] such as the participants' background and general views of defining innovation. The interviewee's comments were restated and incorporated into further questions to check with the interviewer that they had understood correctly[39]. In order to gain more detailed and comprehensive understanding of the descriptions given by participants, they were asked to elaborate their comments with explanations and examples (such as, 'Why do you feel this was?')[39], or fillers (such as 'mmm' or 'yeah') were used by the interviewer to show the participant this was something they were interested in and to encourage them to continue [42]. Towards the end of the interview, participants were given an opportunity to raise issues that were important to them that had not already been covered [41].

Topic guides were developed iteratively during the study period, in consideration of emerging insights from interviews and better ways of phrasing questions[37 43]. For instance, interviewers (non-clinical academics) used more prompts to encourage the surgeons to describe procedures in a non-technical manner [39]. Topic guides were initially very long so were reduced to include more 'mapping' questions that addressed the core components of interest (such as, 'I understand you've conceptualised a new procedure. Can you talk me through this?'). Additional prompts were added from emerging findings (i.e. colleagues' reactions to procedures and written information). With permission, interviews were audio recorded using an encrypted digital dictaphone.

All interviews were audio recorded except one where the interviewee requested not to be recorded and therefore written notes were taken. All interviews were conducted by one of two trained and experienced qualitative researchers (JZ or DE). JZ is a male Senior Research Associate with a background in public health research. DE is a female Research Fellow with experience working on surgical trials. She is also a member of the QuinteT research group, which uses qualitative research methods to optimise recruitment and informed consent to randomised controlled trials (RCTs). JZ and DE have several years of experience conducting qualitative research, and each have a PhDs in health-related research.

Data analysis

Interview recordings were transcribed and de-identified so that names and identifiable information were removed. Data were imported into a data management program (NVivo version 11) to

For peer review only - http://bmjopen.bmj.com/site/about/guidelines.xhtml

organise the data systematically, allow for quick searching and refining of codes and categories, increase the transparency of the analysis process and enable team comparisons of coding [33 44]. Thematic analysis was undertaken using the constant comparison technique of grounded theory [21 45]. First, analysts (JZ, DE & SP) read and re-read transcripts before independently coding a sample of five transcripts to begin familiarising themselves with the data [33 46]. Interesting features were coded, whereby a segment of data was assigned a code [43], and on-going potential ideas were noted at every stage of analysis[47]. Data coding was compared to ensure consistency and rigour in the findings[48], whereby differences were discussed thoroughly until a consensus was met and an initial coding frame was developed. The coding frame was then independently applied to three transcripts by JZ, DE and SP. High consistency between analysts indicated the coding frame appropriately captured the data. JZ then applied the coding frame to the remaining transcripts, adding additional codes if important data were missed by the original coding frame. To form preliminary themes and subthemes, codes were collated into hierarchical groups[49]. Emerging findings were regularly discussed with JMB and NB (both academic surgeons), with reference to the raw transcripts, to ensure they fully encapsulated the meaning of the data[48]. Finally, a descriptive account of the themes and subthemes, which included representative quotes, was developed.

<u>Results</u>

Participants

Of the 83 participants that were approached, 42 participants were recruited (those who did not participate were unable to find time for an interview or did not respond to study invites). The final sample included 26 surgeons and 16 governance representatives from across 23 NHS Trusts. Trusts varied in geographical location, type/size (i.e. teaching, specialist, large, multi-centre) and foundation status. Governance representatives' roles within trusts and NICE varied (e.g. medical director, director for quality improvement, head of governance), however, all played a role in regulating the introduction of new surgical procedures and/or devices. At the time of interview, all but one (retired) surgeon worked as a consultant within the NHS. Surgical specialities included cardiothoracic, gastrointestinal, breast, ophthalmology, neurosurgery, urology and orthopaedics. Participants were mostly male (87%). Interviews were conducted over the phone (55%) or face-to-face at participants' place of work or at a university. Interviews lasted an average of 43 minutes (range 22 – 112 minutes, standard deviation 17 minutes).

Analysis

For peer review only

For peer review only - http://bmjopen.bmj.com/site/about/guidelines.xhtml

Findings related to informed consent are presented under four main themes: *difficulty defining innovation*, d*iffering views on what – and how - patients should be told, the challenges of discussing innovation,* and *managing patient expectations*. All quotations are followed by participants' unique identification number, gender, surgical speciality and trust identification number.

Difficulty defining innovation

Surgeons and governance representatives found it challenging to define surgical innovation and considered the term 'innovation' to encompass a spectrum of changes to surgical healthcare. Examples ranged from changing patient pathways through to first in human procedures. Consequently many felt innovation should be viewed as a continuum, rather than a binary concept:

> *"Well there's a spectrum isn't there, I mean it's difficult to define. So something that is innovative to me is something that is new, it's a new way of looking at it, it's a slightly different paradigm, a different way of thinking. And an adaptation is just a sort of a natural evolution of something that's already pre-existing, I suppose that's how I would define them, but clearly it's a spectrum isn't it?" (Surgeon 18, General surgery, Male, Trust 3)*

There was variation in how the procedure had been introduced in participants' hospitals. Within this, surgeons' own experiences included requesting approval from their trust's new procedure committee, conducting the procedure in the context of a research study or deeming that approval was not necessary as the procedure was a variation of an existing one (from either judgement of the surgeon or confirmation from a committee). Table 1 highlights that the challenges of defining innovation, alongside the required technical knowledge of the procedure, could cause uncertainty as to what governance was required.

Table 1: Participants' experiences of governance for new procedures

	**Example quote**
**Procedure considered and approved by trust's new procedure committee**	*"There's one surgeon in [European country] who described the procedure, and the surgeon in [Asian country] who had done some of these procedures, but not really clear on how they'd done it. I contacted the [European] surgeon. He gave me some pointers, so I discussed it with my colleagues, and of course put it through the governance processes as a novel and a new procedure. And then we did it." (Surgeon 31, Colorectal, Male, Trust 8)*
**Approval from trust's new procedure committee deemed unnecessary as felt to be a variation of established procedure**	*"We asked about it to the new procedures committee, but they said well it's basically [variation of established procedure] isn't it. The technicalities of it are lost on non-thoracic surgeons really, which is good." (Surgeon 31, General surgery, Male, Trust 8)*

For peer review only - http://bmjopen.bmj.com/site/about/guidelines.xhtml

	"There's a new technique that's probably done by no more than 9 or 10 surgeons in the country. Again, it's a new technique, it's only been reported in the last 3 or 4 years. [...] Because it was under the umbrella of [broader procedure], I didn't have to go through any, so within the hospital there's a board about new techniques, you have to sort of, clinical effectiveness group, you have to have that approved. But, because this was a variation, we didn't need that approval." (Surgeon 22, Male, Orthopedics, Trust 7)
**Procedure evaluated in the context of a research study**	*"We were looking [procedure], and so that was in the guise of a randomised double-blind trial that we put through Ethics. So that would be innovative. We've got an Ethics Committee, and as part of that if you were going to be doing something really brand new that would have to be conducted as part of a trial, so, but when there's a blur between doing something slightly different then no I don't think it's the place for that [...] I can't think of anything where they've been involved in, you know, anything that we're doing." (Surgeon 46, Male, Orthopedics, Trust 22)*

Although participants initially stated that patients should be informed about new procedures, some drew upon experiences that contradicted this as they reflected upon the complexity of defining innovation:

"I think absolutely they should be informed, I think that's a tricky one actually… when would I inform a patient about a new instrument I was using? So we don't- if- if you're not trialling the instrument to see if it's safe or more effective or less effective, then I don't think they need to be informed." (Surgeon 4, Male, General and upper gastrointestinal, Trust 12)

"We kind of created our own version of how you do that operation […] So, it's not particularly been described before by surgeons and it's not the world's most different, but it's got a few little innovative techniques. So, that was an operation we designed that was new […] I don't even explain how that's different to [standard procedure] because I think it's far too technically nuanced for a patient to understand… We view it as just a variation of [standard procedure]." (Surgeon 22, Thoracic, Male, Trust 7)

Several governance representatives also described instances where they had become aware of patients not being fully informed about new procedures and emphasised the need for improved information provision:

"So we then audit the first case to make sure that the clinician has been up front with the individual about the fact that this is the first time that this particular procedure has been undertaken…if they haven't, there's been a couple of

For peer review only - http://bmjopen.bmj.com/site/about/guidelines.xhtml

occasions when they haven't, we tell them off and say do better next time and we audit the second case or whatever, so we're confident they've got it right." (Governance representative 29, Male, Trust 5)

"We have had a couple of near misses … one was a surgeon inviting another surgeon from another hospital to come and show him how to do a procedure, that person did not have an honorary contract with the Trust therefore shouldn't have been operating and nobody knew that that was occurring. And we have had a rep [industry representative] coming into theatres to bring some samples of a new piece of equipment and most certainly patients weren't consented that it was going to be used on them … So I think there is increasing awareness now, certainly when business cases are presented, they do need to follow this process if that's what's going to happen. However there is also, can I put my hand on my heart and say actually the surgeon's not going to invite a rep into theatre tomorrow? I probably couldn't, but I do know that the theatre staff feel more empowered to be able challenge that behaviour, so actually if I knew that somebody was going to use a piece of equipment they've never used before I would probably be asking the question as to why." (Surgeon 9, Upper gastrointestinal, Male, Trust 3)

Differing views on what – and how - patients should be told

There was consensus from all participants that if a procedure was deemed innovative, patients should be provided with additional information extending beyond that given during routine surgical consultations. Within this, the potential risks and benefits, alternative treatment options, and the novel status of the procedure were universally regarded as essential information that should be discussed.

"We tell them that it's a new procedure and previously we've told them how many we've done prior to this" (Surgeon 5, Thoracic, Male, Trust 5)

"You have to give them options, you have to let them know what the evidence base is." (Surgeon 46, Trauma & Orthopaedic, Male, Trust 22)

There was variation in views as to whether other information - such as the evidence base (or lack thereof), operative experience, training, safety precautions implemented in theatre and national guidance - should be disclosed. Taken together, there appeared to be uncertainty at precisely how much to tell patients:

"Sometimes I tell them too much or tell them... I mean, the latest, er, ruling of Montgomery as I understand it is that they need to be told what they would want to know. Bloody hell. But then you've got to judge what they want to know and you've

For peer review only - http://bmjopen.bmj.com/site/about/guidelines.xhtml

got to have some kind of communication with them and reasonably understand what it is they want to know." (Surgeon 31, Colorectal, Male, Trust 8)

"There is a difficult balance I think, and I'm not sure we've got it [consent] right yet actually." (Governance representative 7, Male)

Many interviewees felt strongly that patients should be provided with verbal (i.e. discussions during consultation) *and* written information (i.e. via a patient information leaflet (PIL)) when being offered innovative surgery. In line with this, most governance representatives reported that innovative procedures could only be introduced if an appropriate PIL had been developed (although there was variation as to whether this was reviewed by the committee).

"We will insist that there is a patient guide…an information sheet for the patient…It [PIL] will talk about the relative inexperience of the team, saying that this is new to us, but that we're doing it under conditions where we've got an expert coming to help us, we've been to appropriate training, it's been approved by the organisation as something that we think will be safe, but that it is a new way of doing things." (Governance representative 24, Male, Trust 9)

However, other trusts had no mandatory requirements for written information or did not review written documentation as part of the application to introduce a new procedure:

"Obviously in situations in which the technique might be new then that's (PIL) not something I've specifically addressed but probably should do." (Governance Representative 48, Male, Trust 24)

"I definitely think they should know it's a new procedure. The way we probably tell (patients) is only in the consent process verbally, I don't recall seeing that in any of our written leaflets." (Governance representative 47, Female, Trust 18)

The challenges of discussing innovation

As previously discussed, surgeons felt a need to deliver additional information during consultations for innovative surgery. However, data suggest that some surgeons may find this challenging:

"it's just trying to get across not only this fact that this is what it is, but the fact that it's newer than something else which as a standard we use. That's quite difficult trying to get that across, and some patients will understand better than others." (Surgeon 42, Neurosurgery, Male, Trust 13)

At a practical level, surgeons reported finding it difficult to establish what information is essential and how much detail is enough. These issues may be further compounded when faced with time constraints, which limit surgeons' ability to have long and/or multiple discussions with patients:

For peer review only - http://bmjopen.bmj.com/site/about/guidelines.xhtml

"We don't have much time …I'm finding that I'm over talking far too much information, but I need to make them aware that they're essentially guinea pigs." (Surgeon 23, Colorectal, Female, Trust 2)

Delivering information to a variety of patients who may differ in their ability to understand complex - and potentially very technical - information was a further challenge. To help overcome this, some surgeons felt it was important to simplify technical information and, when possible, provide alterative mediums of information to aid decision making:

"It's [surgical procedure] such a complex thing to be explaining to people so you have to try and bring it down to simplistic terms but at the same time give it in a way that actually they can process the salient points of the information." (Surgeon 46, Trauma & Orthopaedic, Male, Trust 22)

"I think one of the most important things about when you're making this change is that you give people different forms of access, and of course now it's easy. There are YouTube videos of stuff." (Surgeon 43, Orthopaedic, Male, Trust 2)

Surgeons acknowledged that innovative surgery was associated with uncertainties, including a lack of knowledge surrounding risks, benefits and long-term outcomes. Some felt that discussing uncertainties may conflict with the professional norms within surgery:

"This is what surgeons find hard. You have to say, "I don't know," and we hate that. But actually, we should say it more often. We should say it more often." (Surgeon 4, General and upper gastrointestinal, Male, Trust 12)

As a result, when faced with uncertainty surrounding an innovative surgery, surgeons sometimes appeared to disclose their personal opinions during patient consultations:

"They were told that it was a new technique, that we weren't sure what the long-term outcomes were going to be but, we thought that it was better than traditional surgery." (Surgeon 16, Urology, Male, Trust 2)

On the other hand, some expressed a desire to remain impartial and stressed the importance of providing balanced information. Several interviewees described using strategies to help ensure they remained as objective as possible during consultations. Examples discussed included adhering to scripted information and only discussing innovative surgery if a patient specifically requested an alternative to conventional approaches:

"You can word a new procedure, this is amazing, fantastic, amazing, it's phenomenal and there's no risks to it, and all the patients will say sure, yeah sign me up. Whereas, this is what we're trying to look for, this is the benefits, these are

For peer review only - http://bmjopen.bmj.com/site/about/guidelines.xhtml

the potential risks, so on and so forth. Then it becomes much more objective." (Surgeon 25, Thoracic, Male, Trust 14)

Of note however, the language used to describe conventional surgical approaches during interviews highlights the potential for surgeons – albeit unintentionally – to influence treatment choice. For instance, terms such as 'old fashioned' and 'traditional' were used by some when describing how they approached patient information provision:

"I'll tell them, "We're going to try and do it like this. Is that okay? Do you have any objection? The alternative is we're going to do it the old-fashioned way, which is make a big cut in your- on your stomach wall" (Surgeon 4, General & Upper Gastrointestinal Surgeon, Male Trust 12)

Managing patient expectations

Surgeons felt that patients often had preferences for new treatments and expected these to deliver better outcomes. For instance those with experience of introducing robotically assisted surgery commonly reported that patients conveyed a strong preference for this approach. Consistent with this, one interviewee felt that patient demand for robotically assisted surgery was a driving factor for hospitals to invest in robotics.

"Patients took to it [robotically assisted surgery] very well. I thought they might be a bit nervous about it, you know the robot taking over or going crazy…patients like the idea of technology. They equate the robot to being an advanced, superior treatment." (Surgeon 31, Colorectal, Male, Trust 8)

"Patients wanted to have robotic surgery, and if you didn't have a robot, the patients would just go to a neighbouring hospital that did. So, it's really patient driven, so if you wanted to be a centre you have to have a robot." (Surgeon 16, Urology, Male, Trust 2)

In general, interviewees felt patients held a misconception that 'new' surgical procedures and technologies are better than those normally provided. Some surgeons reported that patients had actively sought them out in order to receive innovative surgery:

"They tend to have sought you out, because they want something different than the standard. So they're kind of are willing to accept uncertainty and risk because they've already sought out that uncertainty and risk, if you know what I mean." (Surgeon 9, Upper gastrointestinal, Male, Trust 3)

For peer review only - http://bmjopen.bmj.com/site/about/guidelines.xhtml

Several surgeons were concerned that patients did not understand that innovative procedures may carry uncertainties in terms of risk and benefit. Managing patients' preferences, particularly if they align with the personal preferences of the surgeon, appeared challenging for some:

> *"When does a patient turn around and say, 'Well I'll have the old one'? And the answer is never, and that's difficult, because they are immediately signing themselves up for an experiment. And how you describe that to patients is really difficult, because you've got all of your intrinsic biases yourself. Okay, go on then." (Surgeon 43, Orthopaedic, Male, Trust 2)*

> *"I don't think I've had anyone say, 'no, thank you'. I think most of them have gone, 'I don't mind being a guinea pig'. They always use that phrase (laughter). Makes me feel a little bit uncomfortable." (Surgeon 36, Breast, Female, Trust 4)*

Discussion

This study provides novel insights into surgeons' and governance representatives' views on information provision in the context of innovative surgical procedures and represents a significant contribution to the limited empirical literature. Results highlight inconsistencies in how NHS trusts govern how patients should be informed about innovative surgical procedures. Furthermore, interviews with surgeons suggest a lack of consistency in amount and quality of the provision of verbal and written information prior to delivery of innovative surgical treatment. Data indicate a heavy reliance on surgeons to decide what and how information is communicated to patients within some trusts. This conflicts with the recommendations published by the RCSEng, which suggest oversight committees should discuss information provision and consent with surgeons prior to approvals being granted [9]. Collectively, our results stress the need for better oversight and regulation of information provision in the context of innovative surgery. Without improved standards of information provision, patients' informed consent is potentially compromised. There is, however, particular complexity in understanding what innovation is, and when it might occur, that makes establishing these standards difficult.

Consistent with previous research, our findings suggest that surgeons may struggle to clearly define surgical innovation[50], and this may impact upon their approach to patient information provision and consent. It can represent a continuum from standard practice through to true innovation. Rogers et al [14] demonstrated that surgeons held no uniform view of surgical innovation, and showed the lack of agreement on the distinction between innovation and research. Our study highlights the implications of ambiguity – surgeons may use their own judgement as to what is innovative and therefore not discuss novel components of an operation with patients. In addition, procedures that

For peer review only - http://bmjopen.bmj.com/site/about/guidelines.xhtml

were deemed innovative were sometimes introduced as part of routine clinical practice. This meant that information provided to patients was at the discretion of the surgeon, rather than an ethics review board and no peer review of the process. Birchley et al have suggested that instead of focusing on whether something is 'innovative' or not, the potential risks and the ethical appropriateness of modifying surgical practice should be considered to ensure the safe translation of surgical innovation into clinical practice[30]. Work is ongoing to better conceptualise what innovation is and determine when it is happening [51] - although in the meantime we rely on surgeons' views on what is innovative and how that influences their practice of information provision.

Prior research has shown patients do not always feel fully informed about the different treatment options available to them[52 53]. It is likely that the added complexity associated with innovative treatments (e.g. unknown unknown risks and benefits, lack of long-term data, limited surgeon experience of this technique) makes discussing treatments options particularly challenging for surgeons. This has an impact on the process of information provision within the context of gaining informed consent to undergo the procedure. As many innovations are undertaken within the context of clinical practice and not research, then the information provision is at the discretion of the surgeon. Study participants recognised the importance of communicating the risks and benefits, treatment alternatives, and novel status of a procedure to patients. However, it was less common for interviewees to identify prior training, experience with undertaking the procedure, and the evidence base supporting the innovation as important components of consent in information provision. These elements of information provision are recognised as key to gain informed consent [2 9]. The variation observed in this study suggests there is a lack of clarity in guidance for surgeons on what should be disclosed during the content of informed consent discussions in the context of innovative surgery, meaning that some patients are likely to be better informed than others when making treatment decisions. This is a concern given that information provision within the context of informed consent has been linked to patient satisfaction and rates of litigation following surgery[54-56].

Aligning with previous research, our data suggests surgeons may not disclose their personal experience of performing a procedure to patients and this is important to patients[57]. In this study few surgeons identified, or reported discussing, surgical experience and/or training as a component of information provision. While we cannot conclude from the current data, the disclosure of this information could lead to conversations regarding the surgical learning curve. Such discussions could help emphasise to patients that a procedure is novel and there are potential uncertainties in terms of risks and benefits. Training programmes have been developed to enhance informed consent in RCTs[58 59], although none have focused on innovative surgical procedures. Our study suggests that

For peer review only - http://bmjopen.bmj.com/site/about/guidelines.xhtml

For peer review only

surgeons may require support and training to raise awareness of the challenges of discussing novel procedures and provide support to overcome these.

To the best of our knowledge, this study is the first to explore healthcare professionals' views of what information should be shared with patients during informed consent consultations in the context of innovative surgery. An important strength of the study is the sample of both surgeons and governance representatives, recruited from various surgical specialities, trust types and geographical locations. This allowed us to explore information provisioned consent from different perspectives, and provided important insight into the role trust governance plays in patient information provision and consent. Nonetheless, there are potential limitations to the study that should be acknowledged. We cannot conclude exactly what information is communicated to patients during consultations from interview data alone. Future research should capture what actually happens in these interactions by audio recording clinician-patient consultations. Such methods have been used successfully in RCTs to improve the quality of informed consent and recruitment rates[60-62]. Furthermore, studies exploring patients' views, particularly those who are offered or have received innovative surgery, are critical if we are to understand the type of information patients draw upon when making decisions about their care. This work is currently being conducted by our research group as part of a larger research study exploring innovation in surgery[51]. It is hoped that this research will aid the development of 'core information sets' (minimum sets of information that should be discussed with patients), which will ultimately support surgeons to discuss innovation and help catalyse the discussion of issues that are important to individual patients[63].

In conclusion, the study suggests that information provision for innovative surgery presents a challenge for surgeons. Our findings also highlight variation in how NHS trusts govern information provision and consent practice for innovative surgery, and further inconsistencies between the views of surgeons and governance representatives. When innovations are introduced under the auspices of research, there are governance structures (e.g. research ethics committees) in place to help ensure patient information is appropriate. However, innovation and modifications to surgery often takes place without formal research approval, which raises the question of how well informed patients are prior to surgery. While the recent RCSEng guidelines for introducing new techniques and technologies represents an important step towards improving informed consent, our study suggests a need to develop support and training programmes to ensure that surgeons consistently deliver appropriate and comprehensive information to patients in the context of innovative surgery.

Authors' contributions

For peer review only - http://bmjopen.bmj.com/site/about/guidelines.xhtml

JMB conceptualised the idea to study informed consent and information provision in the context of surgical innovation. DE developed the idea for the current study with support from JMB, JZ, SP, SC, NB, BM, AM and KA. NB, JM, AM and BM identified potential participants. JZ and DE conducted the interviews and analysed the data, with regular discussion of data interpretation with SP and JMB. The NIHR Bristol Biomedical Research Centre Surgical Innovation theme is directed by JMB (theme lead) and RH (deputy theme lead). The manuscript was written by JZ, and DE critically reviewed the manuscript and responded to reviewers' comments. All authors commented on the article.

Data availability statement:

All data relevant to the study are included in the article or uploaded as supplementary information.

For peer review only

For peer review only - http://bmjopen.bmj.com/site/about/guidelines.xhtml

Competing interests

None declared

References

1. Committee BvFHM. All ER 118,[1957] 1 WLR 582.
2. Surgeons RCo. Consent: Supported Decision-Making-a good practice guide: RCS London, 2016.
3. Coulter A, Hopkins A, Moulton B. Montgomery v Lanarkshire Health Board: transforming informed consent. *The Bulletin of the Royal College of Surgeons of England* 2017;99(1):36-38.
4. Faden RR, Beauchamp TL. A history and theory of informed consent: Oxford University Press 1986.
5. Chan SW, Tulloch E, Cooper ES, et al. Montgomery and informed consent: where are we now? 2017;357:j2224.
6. Chan SW, Tulloch E, Cooper ES, et al. Montgomery and informed consent: where are we now? 2017;357:j2224. doi: 10.1136/bmj.j2224 %J BMJ
7. Royal College of Surgeons. Learning from invited reviews. 2019
8. Dyer C. Robot assisted surgery is blamed for heart patient's death. 2018;363:k4791. doi: 10.1136/bmj.k4791 %J BMJ
9. Royal College of Surgeons. Surgical innovation, new techniques and technologies 2019.
10. Bracken-Roche D, Bell E, Karpowicz L, et al. Disclosure, Consent, and the Exercise of Patient Autonomy in Surgical Innovation: A Systematic Content Analysis of the Conceptual Literature. *Accountability in Research* 2014;21(6):331-52. doi: 10.1080/08989621.2013.866045
11. Di Paolo M, Boggi U, Turillazzi E. Bioethical approach to robot-assisted surgery. 2019
12. Reitsma A, Moreno J. *J Am Coll Surg* 2002;194(null):792.
13. Char SJL, Hills NK, Lo B, et al. Informed consent for innovative surgery: A survey of patients and surgeons. *Surgery* 2013;153(4):473-80. doi: https://doi.org/10.1016/j.surg.2012.08.068
14. Rogers WA, Lotz M, Hutchison K, et al. Identifying surgical innovation: a qualitative study of surgeons' views. *Ann Surg* 2014;259(2):273-78.
15. Apramian T, Watling C, Lingard L, et al. Adaptation and innovation: a grounded theory study of procedural variation in the academic surgical workplace. *J Eval Clin Pract* 2015;21(5):911-18. doi: 10.1111/jep.12398
16. Danjoux NM, Martin DK, Lehoux PN, et al. Adoption of an innovation to repair aortic aneurysms at a Canadian hospital: a qualitative case study and evaluation. *BMC Health Serv Res* 2007;7(1):182. doi: 10.1186/1472-6963-7-182
17. Sharma B, Danjoux NM, Harnish JL, et al. How are decisions to introduce new surgical technologies made? Advanced laparoscopic surgery at a Canadian community hospital: A qualitative case study and evaluation. *Surg Innov* 2006;13(4):250-6. doi: 10.1177/1553350606296341 [published Online First: 2007/01/18]
18. Johnson J, Hutchison K. They Know How to Work It, That's Their Focus in Life: The Complex Role of Industry Representatives in Surgical Innovation. *Journal of Empirical Research on Human Research Ethics* 2018;13(5):461-74. doi: 10.1177/1556264618785037
19. Cousins S, Richards H, Zahra J, et al. Introduction and adoption of innovative invasive procedures and devices in the NHS: an in-depth analysis of written policies and qualitative interviews (the INTRODUCE study protocol). 2019;9(8):e029963. doi: 10.1136/bmjopen-2019-029963 %J BMJ Open
20. Health Do. Health Service Circular (HSC2003/011) – The Interventional Procedures Programme 2003 [Available from: https://webarchive.nationalarchives.gov.uk/20120503190439/http://www.dh.gov.uk/prod_consum_dh/groups/dh_digitalassets/@dh/@en/documents/digitalasset/dh_4064925.pdf accessed 12/02/2018.
21. Glaser BG, Strauss AL. The Discovery of Grounded Theory. Aldine Transaction: USA 1967.

For peer review only - http://bmjopen.bmj.com/site/about/guidelines.xhtml

22. Krippendorff KJHcr. Reliability in content analysis: Some common misconceptions and recommendations. 2004;30(3):411-33.
23. Miles MB, Huberman AM, Huberman MA, et al. Qualitative data analysis: An expanded sourcebook: sage 1994.
24. O'Brien BC, Harris IB, Beckman TJ, et al. Standards for reporting qualitative research: a synthesis of recommendations. *Academic medicine : journal of the Association of American Medical Colleges* 2014;89(9):1245-51. doi: 10.1097/acm.0000000000000388 [published Online First: 2014/07/01]
25. Patton MQJQsw. Two decades of developments in qualitative inquiry: A personal, experiential perspective. 2002;1(3):261-83.
26. Dempster M. A Research Guide for Health and Clinical Psychology: Palgrave Macmillan 2011.
27. Reitsma AM, Moreno JD. *The American Journal of Bioethics* 2003;3(null):49.
28. Johnson J, Rogers W. *J Med Ethics* 2011;38(null):9.
29. Hutchison K, Rogers W, Eyers A, et al. Getting Clearer about surgical innovation: a new definition and a new tool to support responsible practice. *Ann Surg* 2015;262(6):949-54.
30. Birchley G, Huxtable R, Ives J, et al. Have We Made Progress in Identifying (Surgical) Innovation? *The American Journal of Bioethics* 2019;19(6):25-27. doi: 10.1080/15265161.2019.1602181
31. Fassinger REJJocp. Paradigms, praxis, problems, and promise: Grounded theory in counseling psychology research. 2005;52(2):156.
32. Sandelowski MJRin, health. Sample size in qualitative research. 1995;18(2):179-83.
33. Braun V, Clarke V. Successful qualitative research: A practical guide for beginners: sage 2013.
34. Bowen GA. Naturalistic inquiry and the saturation concept: a research note. 2008;8(1):137-52. doi: 10.1177/1468794107085301
35. Willig C. Applied discourse analysis: Social and psychological interventions: Open University Press 1999.
36. Burman E. Minding the Gap: Positivism, Psychology, and the Politics of Qualitative Methods. 1997;53(4):785-801. doi: 10.1111/j.1540-4560.1997.tb02461.x
37. Smith JAJRmip. Semi-structured interviewing and qualitative analysis. 1995
38. Britten NJB. Qualitative research: qualitative interviews in medical research. 1995;311(6999):251-53.
39. Rubin HJ, Rubin IS. Qualitative interviewing: The art of hearing data: sage 2011.
40. Hammersley P, Atkinson M. Ethnography: principles and practice. London: Routledge 1983.
41. Brinkmann S, Kvale S. Interviews: Learning the craft of qualitative research interviewing: Sage Thousand Oaks, CA 2015.
42. Fielding N, Thomas H. Qualitative interviewing. 2008
43. Charmaz K, Belgrave LJTShoirTcotc. Qualitative interviewing and grounded theory analysis. 2012;2:347-65.
44. Pope C, Ziebland S, Mays N. Analysing qualitative data. *BMJ* 2000;320(7227):114-16. doi: 10.1136/bmj.320.7227.114
45. Seale C, Silverman DJTEJoPH. Ensuring rigour in qualitative research. 1997;7(4):379-84.
46. Braun V, Clarke V. Using thematic analysis in psychology. *Qualitative Research in Psychology* 2006;3(2):77-101. doi: 10.1191/1478088706qp063oa
47. Charmaz K. Constructing grounded theory: A practical guide through qualitative analysis: sage 2006.
48. Barbour RSJB. Checklists for improving rigour in qualitative research: a case of the tail wagging the dog? 2001;322(7294):1115-17.
49. Dey I. Qualitative data analysis: A user friendly guide for social scientists: Routledge 2003.
50. Rogers WA, Lotz M, Hutchison K, et al. Identifying surgical innovation: a qualitative study of surgeons' views. *Ann Surg* 2014;259(2):273-8. doi: 10.1097/SLA.0b013e31829ccc5f [published Online First: 2013/06/22]

For peer review only - http://bmjopen.bmj.com/site/about/guidelines.xhtml

51. McNair AGK, Main BJ, Elliott D. Comment on: Bioethical approach to robot-assisted surgery. 2020;107(1):150-50. doi: 10.1002/bjs.11413
52. Mitchell S, Gass J, Hanna M. How Well Informed Do Patients Feel about Their Breast Cancer Surgery Options? Findings from a Nationwide Survey of Women after Lumpectomy and/or Mastectomy. *Journal of the American College of Surgeons* 2018;226(2):134-46.e3. doi: 10.1016/j.jamcollsurg.2017.10.022 [published Online First: 2017/12/17]
53. Kinnersley P, Phillips K, Savage K, et al. Interventions to promote informed consent for patients undergoing surgical and other invasive healthcare procedures. 2013(7)
54. Ford KE, Cooper LRL. Learning from lawsuits: Ten-years of NHS litigation authority claims against 11 surgical specialities in England. *The Surgeon* 2018;16(1):27-35. doi: https://doi.org/10.1016/j.surge.2016.03.009
55. Veerman MM, van der Woude LA, Tellier MA, et al. A decade of litigation regarding surgical informed consent in the Netherlands. *Patient Education and Counseling* 2019;102(2):340-45. doi: https://doi.org/10.1016/j.pec.2018.08.031
56. Hallock JL, Rios R, Handa VL. Patient satisfaction and informed consent for surgery. *American journal of obstetrics and gynecology* 2017;217(2):181.e1-81.e7. doi: 10.1016/j.ajog.2017.03.020 [published Online First: 2017/04/02]
57. Lee Char SJ, Hills NK, Lo B, et al. Informed consent for innovative surgery: a survey of patients and surgeons. *Surgery* 2013;153(4):473-80. doi: 10.1016/j.surg.2012.08.068 [published Online First: 2012/12/04]
58. Townsend D, Mills N, Savović J, et al. A systematic review of training programmes for recruiters to randomised controlled trials. *Trials* 2015;16:432. doi: 10.1186/s13063-015-0908-6
59. Elliott D, Husbands S, Hamdy FC, et al. Understanding and Improving Recruitment to Randomised Controlled Trials: Qualitative Research Approaches. *Eur Urol* 2017;72(5):789-98. doi: https://doi.org/10.1016/j.eururo.2017.04.036
60. Elliott D, Husbands S, Hamdy FC, et al. Understanding and Improving Recruitment to Randomised Controlled Trials: Qualitative Research Approaches. *European urology* 2017;72(5):789-98. doi: 10.1016/j.eururo.2017.04.036 [published Online First: 2017/06/06]
61. Rooshenas L, Paramasivan S, Jepson M, et al. Intensive Triangulation of Qualitative Research and Quantitative Data to Improve Recruitment to Randomized Trials: The Quintet Approach. 2019;29(5):672-79.
62. Donovan JL, Rooshenas L, Jepson M, et al. Optimising recruitment and informed consent in randomised controlled trials: the development and implementation of the Quintet Recruitment Intervention (QRI). *Trials* 2016;17(1):283. doi: 10.1186/s13063-016-1391-4
63. Main BG, McNair AGK, Huxtable R, et al. Core information sets for informed consent to surgical interventions: baseline information of importance to patients and clinicians. *BMC medical ethics* 2017;18(1):29. doi: 10.1186/s12910-017-0188-7

For peer review only

For peer review only - http://bmjopen.bmj.com/site/about/guidelines.xhtml

Reporting checklist for qualitative study.

Based on the SRQR guidelines.

Instructions to authors

Complete this checklist by entering the page numbers from your manuscript where readers will find each of the items listed below.

Your article may not currently address all the items on the checklist. Please modify your text to include the missing information. If you are certain that an item does not apply, please write "n/a" and provide a short explanation.

Upload your completed checklist as an extra file when you submit to a journal.

In your methods section, say that you used the SRQRreporting guidelines, and cite them as:

O'Brien BC, Harris IB, Beckman TJ, Reed DA, Cook DA. Standards for reporting qualitative research: a synthesis of recommendations. Acad Med. 2014;89(9):1245-1251.

		Reporting Item	Page Number
**Title**			
	#1	Concise description of the nature and topic of the study identifying the study as qualitative or indicating the approach (e.g. ethnography, grounded theory) or data collection methods (e.g. interview, focus group) is recommended	1
**Abstract**			
	#2	Summary of the key elements of the study using the abstract format of the intended publication; typically includes background, purpose, methods, results and conclusions	2
**Introduction**			
Problem formulation	#3	Description and signifcance of the problem / phenomenon studied: review of relevant theory and empirical work; problem statement	4

For peer review only

Purpose or research question	#4	Purpose of the study and specific objectives or questions	5
**Methods**			
Qualitative approach and research paradigm	#5	Qualitative approach (e.g. ethnography, grounded theory, case study, phenomenolgy, narrative research) and guiding theory if appropriate; identifying the research paradigm (e.g. postpositivist, constructivist / interpretivist) is also recommended; rationale. The rationale should briefly discuss the justification for choosing that theory, approach, method or technique rather than other options available; the assumptions and limitations implicit in those choices and how those choices influence study conclusions and transferability. As appropriate the rationale for several items might be discussed together.	7
Researcher characteristics and reflexivity	#6	Researchers' characteristics that may influence the research, including personal attributes, qualifications / experience, relationship with participants, assumptions and / or presuppositions; potential or actual interaction between researchers' characteristics and the research questions, approach, methods, results and / or transferability	6; 8
Context	#7	Setting / site and salient contextual factors; rationale	6
Sampling strategy	#8	How and why research participants, documents, or events were selected; criteria for deciding when no further sampling was necessary (e.g. sampling saturation); rationale	5-6
Ethical issues pertaining to human subjects	#9	Documentation of approval by an appropriate ethics review board and participant consent, or explanation for lack thereof; other confidentiality and data security issues	5
Data collection methods	#10	Types of data collected; details of data collection procedures including (as appropriate) start and stop	5-7

For peer review only

		dates of data collection and analysis, iterative process, triangulation of sources / methods, and modification of procedures in response to evolving study findings; rationale	
Data collection instruments and technologies	#11	Description of instruments (e.g. interview guides, questionnaires) and devices (e.g. audio recorders) used for data collection; if / how the instruments(s) changed over the course of the study	6-7
Units of study	#12	Number and relevant characteristics of participants, documents, or events included in the study; level of participation (could be reported in results)	8
Data processing	#13	Methods for processing data prior to and during analysis, including transcription, data entry, data management and security, verification of data integrity, data coding, and anonymisation / deidentification of excerpts	7-8
Data analysis	#14	Process by which inferences, themes, etc. were identified and developed, including the researchers involved in data analysis; usually references a specific paradigm or approach; rationale	7-8
Techniques to enhance trustworthiness	#15	Techniques to enhance trustworthiness and credibility of data analysis (e.g. member checking, audit trail, triangulation); rationale	7-8
**Results/findings**			
Syntheses and interpretation	#16	Main findings (e.g. interpretations, inferences, and themes); might include development of a theory or model, or integration with prior research or theory	8-14
Links to empirical data	#17	Evidence (e.g. quotes, field notes, text excerpts, photographs) to substantiate analytic findings	8-14
**Discussion**			
Intergration with prior work, implications,	#18	Short summary of main findings; explanation of how findings and conclusions connect to, support, elaborate on, or challenge conclusions of earlier	15-17

For peer review only

transferability and contribution(s) to the field		scholarship; discussion of scope of application / generalizability; identification of unique contributions(s) to scholarship in a discipline or field	
Limitations	#19	Trustworthiness and limitations of findings	16
**Other**			
Conflicts of interest	#20	Potential sources of influence of perceived influence on study conduct and conclusions; how these were managed	N/A
Funding	#21	Sources of funding and other support; role of funders in data collection, interpretation and reporting	17

The SRQR checklist is distributed with permission of Wolters Kluwer © 2014 by the Association of American Medical Colleges. This checklist was completed on 12. February 2020 using https://www.goodreports.org/, a tool made by the EQUATOR Network in collaboration with Penelope.ai

For peer review only

Topic guide for interviews with governance representatives

Introduction:

- Thank for taking time
- Not medical professional, want to understand process of implementing new procedures and devices
- No right or wrong answers, can stop the interview at any time
- Take verbal consent

Can you tell me about yourself?

- What is your current position and role?
- Particular areas of interest/expertise?
- How did you become involved in [committee]? When? How found it? What you expected?
- Can you tell me about the committee? What is the role of the committee?
  - Who involved? How often meet?

As you know, we are looking at innovation in surgery. Can you talk me through how you would define innovation? *(Newness; degree of change, level of risk, impact?)*

Can you talk me through how applications are processed, from submissions to decision-making?

- What is the process for introducing surgical innovation in [site]?
- Do you think there are similarities/difference between innovation and research? In what ways?
- How many applications do you receive each year?
- What information is provided? *(What is compulsory?)*
- How are they considered?
  - Potential benefit? Safety (and how quantified?) Evidence? Cost? Patient acceptability?
- Why would an application be declined? Example? Agreement between committee members?
- What happens to those procedures that are declined? Is there an option to appeal?

What's your understanding of how these procedures are integrated into practice?

- What happens once a technique/device is approved by the committee?
- If approved, is there any monitoring? How? When?
- What if there is a problem with a novel procedure?
- Is there any guidance for communicating about novel procedures?
- What do you think patients are told about the novel component? What should they be told - What aspects important to communicate?
- What is included on patient info sheets provided

Your views on the committee

- How do you feel about being involved in the decision-making process?
- Are there any particularly challenging aspects?
- What are the strengths of the committee?
- Do you think it could be improved in anyway?

Ending the interview

- Is there anything else that I haven't mentioned?
- Anyone else you recommend I could contact?

For peer review only

- Before we finish (if not covered) – role (registrar/consultant), years in your profession, highest qualification

For peer review only

Topic guide for interviews with surgeons

Introduction:
- Thank you for taking time
- Not medical professional, want to understand perspectives of innovation
- No right or wrong answers, can stop the interview at any time
- Take verbal consent

Can you tell me about yourself?
- What is your current position and role?
- Can you describe the range /type(s) of surgical procedures you undertake?
- Particular areas of interest/expertise?

As you know, we are looking at innovation in surgery. Can you talk me through how you would define innovation? *(Newness; degree of change, level of risk, impact?)*

Have you conceptualised a new procedure/device/modification of a procedure?
- How did it come about? When?
- Would you regard this as an innovation or a modification?
- Why do you feel it's needed?
- Who is suitable for the procedure? *(How determined?)*
- What treatment options are currently available to them? What happens if patients don't get the procedure? (Are they disadvantaged in any way?)
- What do you hope will be the outcomes from the new procedure? How estimated?

Do you think patients should be informed about new procedures/devices?

- What does 'informed consent' mean to you?
- Is there any guidance for communicating novel procedure?
- What should they be told? Extent that procedure is novel? Uncertainty? Risks? Harms?
- Is informing patients about a new procedure challenging in anyway?
- How do you think patients will respond to the procedure?
- How much of a modification would you need to do to tell a patient something was new?
- When do you stop telling them it is new?

Looking ahead
- Do you think any subgroups of patients would be more suited to the procedure? Are there any that you would feel less comfortable with having the procedure?
- Do you have any concerns about the procedure? Risks to patients? Costs?
- Do you have any plans to disseminate the results? How? All results (good and bad)? Where? When? Who's responsible for this?

Ending the interview
- Is there anything else that I haven't mentioned?
- Anyone else you recommend I could contact?
- Thank for time, summarise key points.

For peer review only
